# Micronutrient content drives elementome variability amongst the Symbiodiniaceae

**DOI:** 10.1186/s12870-022-03512-0

**Published:** 2022-04-09

**Authors:** Emma F. Camp, Matthew R. Nitschke, David Clases, Raquel Gonzalez de Vega, Hannah G. Reich, Samantha Goyen, David J. Suggett

**Affiliations:** 1 Climate Change Cluster (C3), University of Technology Sydney, PO Box 123, Broadway, Ultimo, NSW 2007 Australia; 2 School of Biological Sciences, Victoria University, Wellington, 6012 New Zealand; 3 The Atomic Medicine Initiative, University of Technology Sydney, 15 Broadway, Ultimo, NSW 2007 Australia; 4 Institute of Chemistry, University of Graz, Graz, 8010 Austria; 5 Department of Biological Sciences, University of Rhode Island, 120 Flagg Road, Kingston, RI 02881 USA

**Keywords:** Dinoflagellates, Elemental phenotyping, Elementome, Macronutrients; Micronutrients; Redfield ratio, Symbiodiniaceae

## Abstract

**Background:**

Elements are the basis of life on Earth, whereby organisms are essentially evolved chemical substances that dynamically interact with each other and their environment. Determining species elemental quotas (their elementome) is a key indicator for their success across environments with different resource availabilities. Elementomes remain undescribed for functionally diverse dinoflagellates within the family Symbiodiniaceae that includes coral endosymbionts. We used dry combustion and ICP-MS to assess whether Symbiodiniaceae (ten isolates spanning five genera *Breviolum, Cladocopium, Durusdinium, Effrenium, Symbiodinium*) maintained under long-term nutrient replete conditions have unique elementomes (six key macronutrients and nine micronutrients) that would reflect evolutionarily conserved preferential elemental acquisition. For three isolates we assessed how elevated temperature impacted their elementomes. Further, we tested whether Symbiodiniaceae conform to common stoichiometric hypotheses (e.g., the growth rate hypothesis) documented in other marine algae. This study considers whether Symbiodiniaceae isolates possess unique elementomes reflective of their natural ecologies, evolutionary histories, and resistance to environmental change.

**Results:**

Symbiodiniaceae isolates maintained under long-term luxury uptake conditions, all exhibited highly divergent elementomes from one another, driven primarily by differential content of micronutrients. All N:P and C:P ratios were below the Redfield ratio values, whereas C:N was close to the Redfield value. Elevated temperature resulted in a more homogenised elementome across isolates. The Family-level elementome was (C_19.8_N_2.6_ P_1.0_S_18.8_K_0.7_Ca_0.1_) · 1000 (Fe_55.7_Mn_5.6_Sr_2.3_Zn_0.8_Ni_0.5_Se_0.3_Cu_0.2_Mo_0.1_V_0.04_) mmol Phosphorous^-1^ versus (C_25.4_N_3.1_P_1.0_S_23.1_K_0.9_Ca_0.4_) · 1000 (Fe_66.7_Mn_6.3_Sr_7.2_Zn_0.8_Ni_0.4_Se_0.2_Cu_0.2_Mo_0.2_V_0.05_) mmol Phosphorous ^-1^ at 27.4 ± 0.4 °C and 30.7 ± 0.01 °C, respectively. Symbiodiniaceae isolates tested here conformed to some, but not all, stoichiometric principles.

**Conclusions:**

Elementomes for Symbiodiniaceae diverge from those reported for other marine algae, primarily via lower C:N:P and different micronutrient expressions. Long-term maintenance of Symbiodiniaceae isolates in culture under common nutrient replete conditions suggests isolates have evolutionary conserved preferential uptake for certain elements that allows these unique elementomes to be identified. Micronutrient content (normalised to phosphorous) commonly increased in the Symbiodiniaceae isolates in response to elevated temperature, potentially indicating a common elemental signature to warming.

**Supplementary Information:**

The online version contains supplementary material available at 10.1186/s12870-022-03512-0.

## Background

Symbiodiniaceae are a taxonomically [1] and functionally [2, 3] diverse family of dinoflagellate microalgae considered a model organism to study phylogenetic and phenotypic responses to environmental change [4]. Species within the Symbiodiniaceae thrive across a broad range of marine environments [1] underpinned by their diverse life histories [5–7]. Ubiquitous distribution and varied habitat preferences signifies how Symbiodiniaceae have evolved to diverse abiotic and biotic conditions that ultimately govern their functional diversity [3, 4]. With a few exceptions [8], Symbiodiniaceae are autotrophic, making them important primary producers in coastal ecosystems and essential symbiotic partners [2], particularly to scleractinian corals. Differences in photosynthetic performance have proved central in determining Symbiodiniaceae ecological success [2, 9]. Further, in identifying “ecotypes” adapted to particular factors regulating growth, such as light [2, 10–12], temperature [13–15], and CO_2_ [16]. However, the complex interplay between taxonomically dependent and independent traits [2, 4] challenges identification of the most suitable traits defining Symbiodiniaceae functional diversity.

For higher plants [17–19] and phytoplankton [20–22], decades of research on intercellular elemental quotas (their elementome) has transformed knowledge on both phylogenetic [17] and functional diversity [23, 24]. Elementomes are predicted to be species-specific, reflecting the quantity and stoichiometry of elements required by an organism to survive and grow within any given environment. Elementomes are comprised of both macro- and micro-nutrients, and are determined by traits (unique set of characteristics [25]) of elemental acquisition, storage, and efflux [17, 20, 26, 27]. The ultimate set of structural and functional adaptations required by a species is predicted to result in an optimum elementome (at maximum fitness) – and therefore an “elemental phenotype” [28] governing and defining their biogeochemical niche [26]. It has been postulated that elements which contribute a smaller proportion to cell biomass have greater variability in accumulation potential between species [29] and thus, we hypothesise could be good predictors of species-specific elementomes. In phytoplankton, cell size and elemental stoichiometry often respond in a predictable way to abiotic conditions because of biophysical rules that link growth rates, food web interactions, and biogeochemical cycling [22]. Higher growth rates have been postulated to result in reduced nitrogen-to-phosphorous ratios due to the allocation of phosphorous to RNA synthesis required for protein synthesis (The growth rate hypothesis [30, 31]). Furthermore, the ‘specialised’ photosynthetic apparatus of microalgae, such as reaction centres, and photosynthetic pigment that contributes to functional ecotypes [2], may contribute to unique and predictable elemental content [32, 33]. Elementomes however are not only shaped by ecological function but also by environmental history. Thus, for Symbiodiniaceae maintained long-term in a common culturing environment, it is unknown whether resource requirements are evolutionarily conserved, and identifiable in unique elementomes.

Characterising marine phytoplankton elementomes have led to transformations in the understanding of ocean biogeochemistry [30, 34], as well as species functional responses to stress [35, 36]. However, comparatively little is known of Symbiodiniaceae elementomes and whether elemental phenotypes exist. Isolates within Symbiodiniaceae have immense phylogenetic diversity [1]. They have well described differences in architecture [37] and photosynthesis functioning [38–40], including resource acquisition (e.g., light [2], carbon [37], and iron [41]) that would suggest they likely have evolutionary conserved elemental requirements [17, 26, 30]. Even the most commonly studied stoichiometric relationships, the Redfield ratios (carbon, nitrogen, phosphorus ratios; C:N:P [42, 43]), are rarely reported for Symbiodiniaceae. Only recently has fundamental information on trace metal requirements for Symbiodiniaceae species been reported [41, 44, 45], suggesting that Symbiodiniaceae trace metal requirements vary by ecological guild [41], light [46], and thermal history [45]. Such studies have highlighted how a collective description of cellular elemental quotas can advance our understanding on Symbiodiniaceae physiology and stress responses. The extent of adherence to stoichiometric hypotheses as well as how evolutionarily conserved species requirements are for a broad suite of elements remains untested.

Here, we investigated six key macronutrients and nine micronutrients as part of the elementomes for ten Symbiodiniaceae isolates, spanning eight species. We established whether Symbiodiniaceae isolates have unique elementomes despite long-term maintenance under common nutrient replete conditions. Furthermore, we tested whether Symbiodiniaceae conform to the (i) growth rate hypothesis [31]; (ii) elemental-allometric scaling [30], (iii) photobiological ecotypes having unique elementomes [2], and (iv) whether elements that contribute less to biomass have greater variability between isolates [29] and thus are good predictors of species-specific elementomes. Finally, we assessed how elementomes of three isolates identified as having unique photobiological phenotypes change under a different thermal regime and hence plasticity beyond the fundamental biogeochemical niche.

## Results

### Physiological diversity of Symbiodiniaceae isolates

**Fig. 1:**
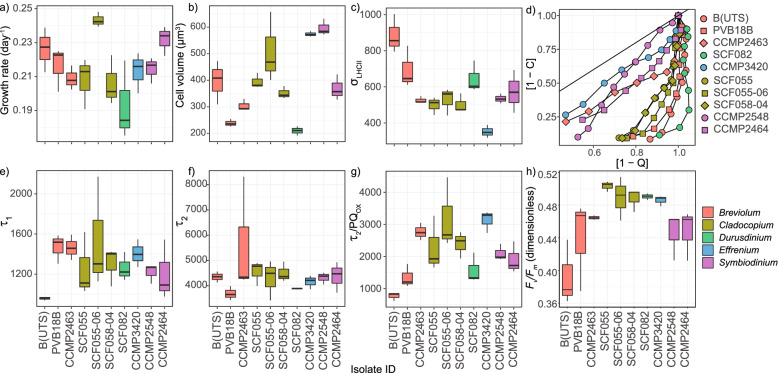
Symbiodiniaceae physiological traits. **a**) Growth rate (μ, d^-1^), **b**) Cell volume (μm^3^), **c**) PSII effective cross-section (σ_LHCII_), **d**) photochemical dissipation (1-C), versus dynamic non-photochemical dissipation (1-Q), **e**) the electron transfer capacity from plastoquinone pigment PQ_A_ to plastocyanin (τ_1_), **f**) the plastoquinone re-oxidation rate (τ_2_), **g**) the plastoquinone re-oxidation rate normalised by the oxidised portion of the plastoquinone pool (τ_2_/PQOX) and **h**) maximum photochemical efficiency (*F*_*v*_/*F*_*m*_) are presented. The calculations for deriving the photobiological parameters are provided in the Methods and Supplementary Information. Lower and upper hinges of each boxplot correspond to the first and third quartiles, and 50% to the median. The upper and lower whiskers of each boxplot extend from the hinge to the largest and smallest value up to 1.5 × the inter-quartile range, respectively

Assessment of division rates, cell volume and photobiological traits confirmed that the 10 Symbiodiniaceae were physiologically diverse. Symbiodiniaceae division rates varied across isolates (*F*_(20)_= 3.669, *p*= 0.007; SI Table 1, Fig. 1a), where SCF082 exhibited the lowest mean (± SE) division rate of 0.19 ± 0.014 μ d^−1^, which differed from the two highest division rates for isolates SCF055-06 (0.24 ± 0.003 μ d^−1^) and CCMP2464 (0.23 ± 0.005 μ d^−1^) (*p*= 0.004, *p*= 0.039 respectively; SI Table 1, Fig. 1a). Division rates were within previously reported ranges for these isolates in nutrient replete medium [2, 37]. Cell volume varied across isolates (*F*_(20)_= 23.272, *p*< 0.001, SI Table 1), and within previously reported ranges [3, 37, 47], ranging from 599 ± 98.9 μm^3^ to 211 ± 38.3 μm^3^ for *Symbiodinium* (CCMP2548) and *Durusdinium* (SCF082) respectively (Fig. 1b). No commonalities were found across genera for the various photobiological traits assessed (Fig. 1c,e-h); however, two putative functional groups emerged when dynamic photochemical quenching (1-C) versus non-photochemical quenching (1-Q) were considered (Fig. 1d). Specifically, isolates CCMP3420, CCMP2548, CCMP2463, and CCMP2464 had different strategies of investing in 1-C versus 1-Q than compared to the other six isolates. Of the remaining photobiological parameters considered (σ_LHCII_, τ_1_, τ_2,_ τ_2_/PQ_OX_), species-specific differences were only observed for τ_2_/PQ_OX_ (Fig. 1g; *F*_(20)_= 7.449, *p*< 0.001; SI Table 1), *F*_*v*_/*F*_*m*_ (Fig. 1h; *F*_(20)_= 4.878, *p*< 0.002; SI Table 1) and σ_LHCII_ (Fig. 1c; *F*_(20)_= 10.631, *p*< 0.001; SI Table 1). Neither of the two electron transfer rates, τ_1_ or τ_2_ (Fig. 1e, f; *p*> 0.050; SI Table 1), differed between isolates.

### The elementome of the ten Symbiodiniaceae isolates

*Absolute Symbiodiniaceae elemental content –* Across all isolates, macronutrient content ranged from 0.09 ± 0.02pg cell^-1^ for Ca to 31.48 ± 5.75pg cell^-1^ for C (SI Fig. 1). Micronutrients were within reported ranges for Symbiodiniaceae [45], with Fe the highest concentration (37.51 ± 6.85 fg cell^-1^), and V the lowest concentration (0.03 ± 0.01 fg cell^-1^) (SI Fig. 1). No notable trends of absolute elemental content were observed at genus level. However, at the isolate level, CCMP2464 exhibited the highest and most variable content for all micronutrients measured except for Cu (which was the highest concentration and most variable for CCMP2463). Thus, no phylogenetic trends were evident for any one element treated in isolation of others. Our results continue in the element normalised to phosphorus ratio format (E:P; see methods) unless otherwise noted, following Redfield ratios, and to allow us to test key stoichiometric hypotheses [21, 22, 27, 41, 44]. Variability in P between isolates (*F*_(19)_= 116.082, *p*< 0.001) can impact E:P, thus, the following results are valid for P-normalised data as per [21, 41]. Across isolates, all elements were in excess in the media relative to the cellular elemental content (SI Fig. 2), demonstrating nutrient replete growth conditions.

**Fig. 2:**
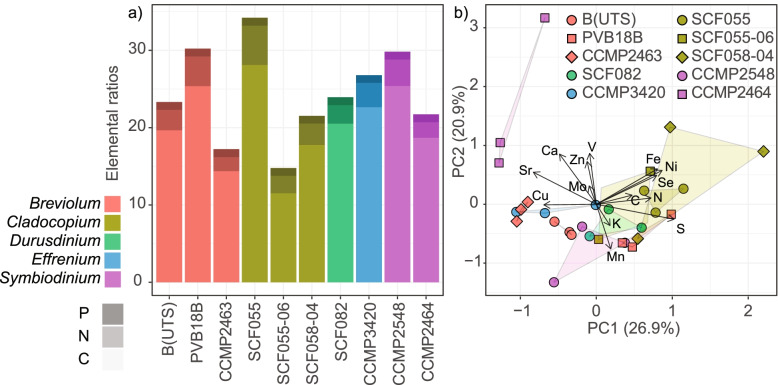
Symbiodiniaceae elementomes at 27.4°C **a** Mean (*n*= 3 per isolate) carbon:nitrogen;phosphorous (C:N:P) ratios for the ten Symbiodiniaceae isolates, with genus denoted by colour, and element by shading (P dark, N medium, C light shading). **b** Principal component analysis (PCA) of the five key macronutrients and nine micronutrients normalised to P, for the ten Symbiodiniaceae isolates. PCA loadings of E:P (visualised as vectors) were scaled to PCA eigenvalues

**Fig. 3:**
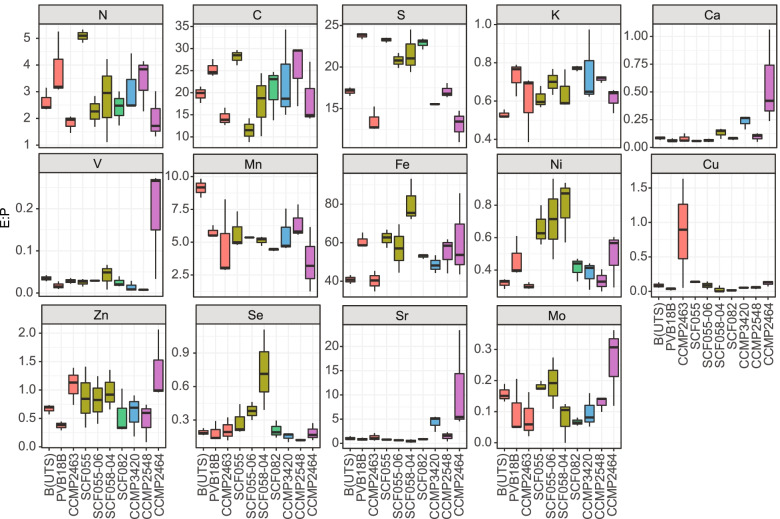
Elemental content plotted as elemental ratios normalised to phosphorus (E:P), of the ten Symbiodiniaceae isolates at 27.4°C. Data is mol:mol for macronutrients (C, N, S, K and Ca) and mmol:mol for micronutrients (V, Mn, Fe, Ni, Cu, Zn, Se, S, Mo). Lower and upper hinges of each boxplot correspond to the first and third quartiles, and 50% to the median. The upper and lower whiskers of each boxplot extend from the hinge to the largest and smallest value up to 1.5 × the inter-quartile range, respectively

*Isolate-specific differences of Symbiodiniaceae elementomes (27.4 °C) –* PCA visualisation of the elementomes (E:P) revealed Symbiodiniaceae isolate-specific differences, but with some overlap predominately from larger variance amongst replicates for SCF058-04 and CCMP2548 (Fig. 2b, SI Tables 2, 3). All three *Cladocopium* isolates clustered together, driven by relatively high Ni:P and Se:P (SI Table 4). Collectively, the first two principal components accounted for 47.8% of the total Symbiodiniaceae elementome variance. The first principal component (PC1) accounted for 26.9% of the total elemental variance, with S:P, Ni:P, Sr:P and Fe:P contributing the largest loadings. ANOVA on the extracted ordination axes for PC1 confirmed differences between isolates (*F*_(19)_= 9.485, *p*< 0.001; SI Table 2). PC2 accounted for 20.9% of the total elementome variance, with V:P, Ca:P, Mn:P, and Zn:P contributing the largest loadings (SI Table 3). ANOVA on the extracted ordination axes for PC2 confirmed differences between isolates (*F*_(19)_= 3.706, *p*= 0.008; SI Table 2), with post-hoc Tukey identifying most differences were due to separation of CCMP2464 to the other isolates (SI Table 2). CCMP2464 exhibited higher Ca:P, Sr:P and V:P than most other isolates (Fig. 3), which explained its separation on the PCA. Collectively, the PCA demonstrates that several isolates can be resolved based on differences in certain E:P ratios; however, some isolates, e.g., SCF058-04, had large elementome variance across replicates that prohibited differentiation from other isolates here.

*Symbiodiniaceae Redfield ratio (27.4 °C) –* All Symbiodiniaceae isolates were characterised by C:N:P all well below the Redfield ratios of 106:16:1 (Fig. 2A). Both the largest (28:5:1; SCF055) and smallest (12:2:1; SCF055-06) C:N:P ratios were observed for isolates of *Cladocopium*. Differences were observed between isolates for N:P (*F*_(19)_ = 2.449, *p*= 0.048) but not C:P (*p*> 0.050: SI Table 4). We also assessed C:N ratios as an elemental signature for cellular storage, where higher C:N suggests elevated storage [48]. C:N was largest for CCMP2464 at 9.43, and lowest for SCF055-06 at 5.12 (Fig. 2A). Overall, C:N:P ratios remained relatively conserved across isolates.

*Expanding beyond Redfield (27.4 °C) –* Other macronutrient E:P ratios across all Symbiodiniaceae isolates, revealed S:P, K:P and Ca:P of 18.8, 0.7 and 0.1, respectively. As for C:N:P, these other macronutrient E:P ratios were highly variable between isolates, with no common trends observed across genera (Fig. 3). S:P and Ca:P were both significantly different across isolates (*F*_(19)_= 26.696, 8.771 respectively, *p*< 0.001; SI Table **4**), but K:P remained relatively conserved (*p*> 0.050; SI Table **4**). Micronutrient-to-P ratios for Ni:P, Se:P, Sr:P, Fe:P, V:P, and Cu:P were all significantly different across isolates (*F* in SI Table 4, *p*< 0.050) and, despite their low concentrations, micronutrient E:P ratios were strong drivers of isolate elementome separation, whereby micronutrient E:P ratios contributed to three of the four highest loadings in both PC1 and PC2 (see SI Table 3). Micronutrient E:P ratios were isolate-specific, but notably all *Cladocopium* isolates exhibited higher Ni:P content than other isolates. Isolate CCMP2464 exhibited higher and the most variable Sr:P, V:P, Mo:P, and Zn:P and CCMP2463 exhibited a higher Cu:P than all other isolates. These observations are consistent with those made on the absolute cellular concentrations of fg cell^-1^. Collective assessment of the elementome of Symbiodiniaceae under nutrient replete conditions, 27.4 ± 0.4 °C, and downwelling irradiance of 150.0 ± 4.05 μmol photons m^-2^s^-1^ defined the average elemental stoichiometry as: (C_19.8_N_2.6_ P_1.0_S_18.8_K_0.7_Ca_0.1_) · 1000 (Fe_55.7_Mn_5.6_Sr_2.3_Zn_0.8_Ni_0.5_Se_0.3_Cu_0.2_Mo_0.1_V_0.04_) mmol P^-1^. Overall, micronutrient E:P are greater contributors to isolate specific differences than macronutrient ratios for these Symbiodiniaceae isolates.

### Symbiodiniaceae conformed to some, but not all stoichiometric hypotheses

(i) *Accumulation capacity for nutrients relative to proportion of cell biomass*. Collectively across isolates, there was less than a seven-fold range in the maximum variability (maximum minimum^-1^) in macronutrient E:P ratios, and the contribution to cell biomass (median E:P) was always greatest for the macronutrients (SI Fig. 3). In general (except for C:P and S:P) maximum minimum^-1^ in E:P across all isolates declined with increasing contribution to cell biomass (median E:P; *R*^*2*^= 0.8, *p* < 0.001; SI Figs. 3 and 4), suggesting that Symbiodiniaceae generally conform to observations from other marine algae of decreasing variability in elemental content with increasing contribution to cell mass [29].
(ii) *The growth rate hypothesis*. N:P ratios have been suggested as a proxy for growth, with lower N:P denoting higher growth rates [30]. However, the lowest and highest N:P was 1.82 and 5.09 for CCMP2463 and SCF055, respectively, and no correlation was observed between N:P ratios and growth rates across isolates (*p*> 0.050; SI Fig. 5).
(iii) *Elemental allometric scaling*. The absolute cellular content of any element did not correlate – and therefore did not allometrically scale – with cell volume (*p*> 0.050; SI Table 5). However, when carbon was plotted it was observed that SCF055-06 was an outlier, and if removed from the analysis, a strong significant positive correlation was observed (*R*^*2*^= 0.71, *p*= 0.005; SI Fig. 6). Variability in cell volume measured for SCF055-06 may explain why it did not correlate with C. With the exceptions of Mo and N that had a positive correlation (*p*< 0.002 and *p*= 0.043 respectively; SI Table 6), intracellular elemental content did not correlate with division rate (*p*> 0.050; SI Table 6). The RDA analysis conducted on E:P data did however identify cell volume as a significant predictor of Symbiodiniaceae elementomes (Table 2).
(iv) *Photobiological ecotypes having unique elementomes.* As discussed above, two putative functional groups of Symbiodiniaceae ecotypes were identified based on strategies of investing in 1-C versus 1-Q. The RDA revealed that investment into photochemical quenching (1-C) was a significant predictor of Symbiodiniaceae elementomes (Table 2). Isolates that had very rapid employment of 1-C over 1-Q (right-hand cluster; Fig.1d) had lower Ca:P and Sr:P and higher S:P, Ni:P and Se:P than the isolates with slower employment of 1-C over 1-Q (left-hand cluster; Fig. 1d, *F* values in SI Table 7, *p*< 0.010).

### Homogenisation of Symbiodiniaceae isolate elementomes under elevated temperature

**Fig. 4:**
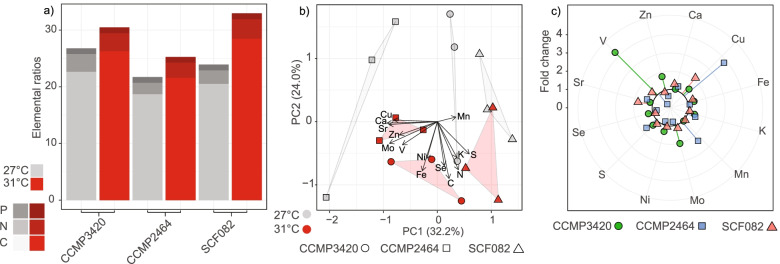
Symbiodiniaceae elementome thermal comparison. **a** Mean (*n*= 3 per isolate) (carbon:nitrogen;phosphorous, C:N:P) ratios for the three Symbiodiniaceae isolates, at 27.4°C (grey colouring) and 30.7°C (red colouring), and element by shading (P dark, N medium, C light shading). **b** Principal component analysis (PCA) of the five key macronutrients and nine micronutrients normalised to P, for the three Symbiodiniaceae isolates at 27.4°C (grey colouring) and 30.7°C (red colouring). PCA loadings of E:P (visualised as vectors) were scaled to PCA eigenvalues. **c** Isolate specific fold change in E:P from 27.4°C to 30.7°C

Three isolates (CCMP3420, SCF082, and CCMP2464) that are taxonomically diverse and demonstrated differences in their photobiological strategies (different strategies of investing in 1-C versus 1-Q; Fig. 1C) were grown under 30.7 °C to further assess how higher growth temperature influenced elementomes. Only SCF082 and CCMP3420 experienced a decline in *F*_*v*_*/F*_*m*_ (*p*< 0.050; SI Table 8) and a slight reduction in division rates (*p*= 0.049; SI Table 9) under the higher temperature. Cell volume was unchanged across all isolates (*p*> 0.050; SI Table 9). At 30.7 °C, C:N:P was not significantly different to that at 27.4 °C (*p*> 0.050; SI Table 10), despite C:P being elevated, across isolates (Fig. 4A). C:N ratios increased across isolates at 30.7 °C (Fig. 4A) indicative of increased cellular storage [48]. CCMP2464 had elevated Ca:P relative to the other two isolates (*p*< 0.05; SI Table 10) and ~3-fold increase in Cu:P at 30.7 °C compared to 27.4 °C (Fig. 4C). PCA revealed changes in elementomes resulting from growth temperature and driven by loadings contributing to PC2 (Fig. 4B), V:P, Ca:P, Mn:P, and Zn:P. Two-way ANOVA on the extracted ordination axes for PC2 confirmed differences across temperatures (*F*_(12)_= 5.854, *p*= 0.032; SI Tables 11, 12) but not isolates (*p*> 0.050). For PC1, there were differences in the extracted ordinations for each isolate (*F*_(12)_= 28.812, *p*< 0.001; SI Table 11), but no observed differences across temperature treatments (*p*> 0.050). PC1 accounted for 32.2% of the total elemental variance, while PC2 accounted for 24.0%. The resulting elemental stoichiometry of Symbiodiniaceae under elevated temperature (30.7 ± 0.4 °C) was: (C_25.4_N_3.1_P_1.0_S_23.1_K_0.9_Ca_0.4_) · 1000 (Fe_66.7_Mn_6.3_Sr_7.2_Zn_0.8_Ni_0.4_Se_0.2_Cu_0.2_Mo_0.2_V_0.05_) mmol P^-1^. Thus overall, under an elevated temperature a convergence (homogenisation) of isolate elementomes was observed (Fig. 4B).

## Discussion

Efforts to resolve the functional diversity of Symbiodiniaceae predominantly focus on their photosynthetic performance [2, 39, 49], thermal responses [15, 50, 51], and master traits of growth rate and cell size [2, 37] – only more recently have efforts begun to consider nutrient acquisition [3, 41, 52]. No study has yet determined the elementome of Symbiodiniaceae, to ascertain whether isolates have unique elemental content that would be an important functional trait of Symbiodiniaceae. In this study, the elementome was defined for Symbiodiniaceae maintained under long-term nutrient replete (luxury uptake) conditions to ascertain whether elementomes are evolutionarily conserved traits. We tested whether, under a different temperature regime “elemental phenotypes” exist for Symbiodiniaceae, and whether Symbiodiniaceae grown in replete medium adhere to postulated stoichiometric principles.

### The isolate-specific elementomes of the Symbiodiniaceae

Elementomes could explain just under 50% of variation between the 10 Symbiodiniaceae isolates, and several isolates (although not all) could be uniquely identified based on their elemental signature. Macronutrient E:P was relatively conserved across isolates (except for S:P), with differences in micronutrient E:P primarily responsible for isolate-specific elementomes (for both E:P and absolute data), a phenomenon documented for other marine algae [21, 22]. There is generally less storage capacity for micronutrients making changes in stoichiometry more identifiable and our observations support recent suggestions that expansion beyond C:N:P is required to resolve a species’ biogeochemical niche [53]. Some isolates, such as CCMP2464 had large variability in elementomes between biological replicates. A recent study advanced single-cell elementomic methods to allow quantification of the C content per cell in a Symbiodiniaceae culture, revealing significant heterogeneity within an isolate [54]. Whether this corresponds to associated bacteria [51], viruses [55] or may even represent elemental phenotypes within a given isolate requires further study.

Despite common long-term (typically exceeding a decade) growth conditions, Symbiodiniaceae isolates have divergent elementomes. This suggests that isolate-specific preferential elemental acquisition is a conserved evolutionary trait [27]. Differences in phylogeny did not resolve elementomes, but there were some commonalities in genera, for example, all *Cladocopium* exhibited elevated Ni:P. We did not observe elementome separation based on ecological guild [41]. Instead, our data suggests that Symbiodiniaceae elementomes result from a complex interplay of phylogeny, host origin, environmental histories, and isolate-species traits. Examining more isolates is required to test this notion comprehensively given the broad ecological and geographic distribution of the Symbiodiniaceae. Further, this study used a common growth temperature for the ten Symbiodiniaceae isolates to allow comparison of their elementomes. However, the selected temperature is unlikely to reflects the optimal growth conditions for all isolates and was the rational to explore whether a different temperature induced a change in the isolate elementomes. Further work is needed to first establish optimal growth conditions for the Symbiodiniaceae isolates and then to assess how this impacts their elemental composition. Exploring how isolate-specific elementomes adjust under different environmental conditions, e.g., different temperatures, light intensities and nutrient conditions will provide knowledge on the plasticity of species elementomes and thus their biogeochemical niche [26].

### Symbiodiniaceae confirm to some, but not all, proposed stoichiometric hypotheses

Some major elemental stoichiometric hypotheses that have been proposed for marine algae were considered for Symbiodiniaceae:

(i) *Symbiodiniaceae possess greater accumulation capacity for nutrients constituting smaller portions of cell biomass*. Fogg & Thake [29] hypothesised greater accumulation capacity for nutrients that contribute a smaller proportion of cell biomass. We observed a significant correlation between the log-median E:P in biomass and the multiplicative range supporting this hypothesis and corroborating results from other marine phytoplankton studies [22, 34, 56]. This particularly held true for the micronutrients, with Fe:P and Mn:P contributing the greatest biomass and having the smallest multiplicative range (SI Fig. 3). For the macronutrients, the two highest E:P ratios (C:P and S:P) fell outside the 95% confidence interval for this correlation, highlighting how other processes besides allocation into cell biomass, including assimilation, and excretion [28], likely influence elemental acquisition.
(ii) *The growth rate hypothesis is not universally applicable to the Symbiodiniaceae*. It predicts a decrease in N:P with increased growth due to allocation of P to RNA synthesis required for protein synthesis [30, 57]. We did not find a decrease in N:P with increasing growth rate, adding to work that refutes the general applicability of the growth rate hypothesis [22], particularly for photosynthetic organisms [58], when P is not a limiting element [57] and can be stored when in excess as compounds such as polyphosphate [59].
(iii) *Cell volumes are consistent predictors of Symbiodiniaceae elementomes*. It is well understood that changes in growth rates impact nutrient requirements [30] and cell size has historically been used as a predictor for nutrient acquisition [60, 61]. Within our study, all but three isolates had similar growth rates, and thus, it is unsurprising that elemental concentrations generally did not (except Mo and N) correlate with growth rates. Cell volume was variable across isolates, but cellular C concentrations correlated with cell volume/size [54]. Larger cells have greater biomass and storage capacity that can be utilised during luxury uptake conditions [62] and can have reduced solute leakage [63]. However, allometric scaling does not always occur because growth depends on other factors such as nutrient source and organism physiology [64]. Furthermore, organisms that are mixotrophic, which has been reported for Symbiodiniaceae [8], can use organic compounds as well as inorganic nutrients that would cause allometric scaling rules to break down [64]. Biological substitution of elements will also cause allometric scaling on an element-by-element basis to break down. Indeed, our results did reveal that when the collective assessment of E:P (the elementome) was considered, cell volume was a good predictor of isolate elemental content. Such findings support the use of elemental ratios in resolving species functional traits since they account for fundamental stoichiometric relationships, and for species-specific bio-elemental substitution [30].
(iv) *Elementomes of the Symbiodiniaceae reflect investment into photochemical (1-C) versus dynamic non-photochemical energy dissipation (1-Q). D*issipation of excitation energy by photochemical quenching (1-C) and cell volume were good predictors of isolate elementomes. These factors have previously been identified [2] as predictive factors of PSII electron transfer rates and in turn growth of Symbiodiniaceae, and thus identification of ecotypes. Our observations support the assertion that larger cells accommodate more photosynthetic machinery for light harvesting and utilisation [65] that allows greater photochemical quenching (1-C) [2] and we postulate that this is further reflected in Symbiodiniaceae nutrient acquisition and ultimate elementomes. Whilst no consistent E:P trace metal ratios were elevated in the isolates that had different strategies of investing into 1-C versus 1-Q, it seems plausible that isolate-specific differences in elemental uptake capacity, preferential elemental substitution [41] and differences in storage capacity [30] explain this result, but requires verification.

### The elementome of Symbiodiniaceae grown under nutrient replete conditions

The elementome for ten Symbiodiniaceae isolates at 27.4°C was (C_19.8_N_2.6_ P_1.0_S_18.8_K_0.7_Ca_0.1_) · 1000 (Fe_55.7_Mn_5.6_Sr_2.3_Zn_0.8_Ni_0.5_Se_0.3_Cu_0.2_Mo_0.1_V_0.04_) mmol P^-1^. Thus, C:N:P is lower, and S:P and Fe:P higher, compared to elementomes for other marine algae [22, 66]. Caution is noted in direct comparisons between elementomes across studies, due to differences in normalisation (e.g. per cell, E:P or E:C), growth medium and abiotic conditions that can impact organism physiology, as well as the oxidation state of elements and their associated complexes. Even so, such comparisons are valuable in identifying major differences or similarities between isolates.

Symbiodiniaceae isolates examined here had C:N:P ratios below that of the Redfield ratios [42, 43], and lower than previous reports for cultured marine phytoplankton [21, 27, 66], including dinoflagellates [52] and *in hospite* Symbiodiniaceae [67]. Values were however within ranges reported for marine algae cultured in nutrient replete media [68]. Our cultures were sampled during exponential growth where there is greater allocation to P-rich assembly machinery and therefore a lower N:P ratio is expected [69]. Our data corroborates research evidencing marine algae commonly exhibit broader C:N:P than the Redfield ratios [52, 56, 69, 70]. Compared to C:P and N:P, C:N (7.3) was similar to the expected Redfield value of 6.6. Stability in C:N versus C:P and N:P has been reported for other marine algae *in situ* [34] and in cultured nutrient replete conditions [56] and likely stems from both C and N comprising major constituents of cellular metabolic processes that account for a large proportion of cellular mass [56]. In contrast, P generally contributes less biomass, is readily substituted [34, 71] and can be highly variable as several species can store excess amounts in large quantities [59, 72]. C:N appears to be well conserved across Symbiodiniaceae [3] and dinoflagellates [52] suggesting consistent essential cellular requirements of C and N.

All Symbiodiniaceae isolates examined here exhibited high S:P (18.8:1) compared to other marine phytoplankton [22, 27, 66]. Sulphur is required by algae for S-containing amino acids [73] and is commonly present in lipid fractions [74]. Symbiodiniaceae also utilise S in forming the compound dimethylsulphoniopropionate in high concentrations compared to other microalgae [75, 76], potentially explaining the high S:P ratios in our study. Fe:P (55.7:1) across our isolates was elevated relative to previous values for Symbiodiniaceae (25-35:1 [44], ~5-25:1 [41]; see SI Fig. 7), as well as other dinoflagellates (~1.8-14:1 [66]). Higher Fe:P in our study likely reflects the high Fe medium content in IMK, with recent work [44] documenting increased intracellular Fe:P with higher Fe medium content. However, Rodriguez & Ho [46] recently demonstrated that the Fe:P of *Fugacium kawagutii* can vary 5-fold depending on the different light regimes at replete conditions (~13-69:1). Therefore, the magnitude of variation between our and previous Fe:P could also be attributed to light conditions (SI Fig. 7). The directionality of differences between the metal:P observed here and among the Symbiodiniaceae varied on an isolate- and metal- basis ([41]; SI Fig. **7**) and there were instances where metal:P measured in our study were less than conspecifics reared in low trace metal conditions. Overall, the within-isolate and within-species variability observed within our study and relative to others reflects the broad ranges of microalgal elemental phenotypes that exist ([41, 66], SI Fig 7).

### Elevated temperature converges the elementome of Symbiodiniaceae isolates

All three isolates continued to grow at the elevated temperature, but in some cases with reduced growth rates and *F*_*v*_*/F*_*m*_, where the elementomes became more similar, primarily due to all isolates having increased uptake of most micronutrients. Reich et al. [45] recently found elevated trace metal content for a more thermally stable Symbiodiniaceae. Under elevated temperature, detoxification of oxygen radicals [15, 77, 78] and possible increased rates of cellular repair and respiration [79] require upregulation of metalloenzymes and thus a greater requirement of trace metals to meet the changing biogeochemical and cellular activities [45]. Interestingly, *Symbiodinium microadriaticum* (CCMP2464) did not exhibit a reduction in growth rate or *F*_*v*_*/F*_*m*_ but did result in higher Ca:P than the other two isolates. Recent work on other microalgae has revealed that increased cytosolic Ca^2+^ alleviated H_2_O_2_-induced oxidative stress by signalling a series of proteins [80] including the heat shock protein calmodulin that has been found to be conserved across Symbiodiniaceae clades [81]. Calcium- and calcium/calmodulin-dependent protein kinases are also important in intercellular signalling [81, 82] that is possibly upregulated during the change in temperature regime and may have facilitated the physiological stability of *S. microadriaticum* at the elevated temperature. Such notions warrant more targeted investigation.

## Conclusions

In summary, we show that evolutionary diverse Symbiodiniaceae retained in culture are characterised by very different elementomes, likely reflective of highly conserved isolate-specific nutrient acquisition and allocation strategies [3, 4, 83]. Further, Symbiodiniaceae elementomes (based on E:P) appear unique compared to other marine microalgae, and only adhere to some of the classical stoichiometric hypotheses derived from diverse microalgal taxa, including those elements (typically micronutrients) contributing least to cell biomass have greatest variability between isolates. Micronutrients appeared key in discriminating these Symbiodiniaceae isolate-specific differences in elemental composition and thus essential to resolving elemental phenotypes and environmental responses (to elevated temperature). Together, these outcomes suggest that quantifying elemental content, in particular micronutrients, likely enables greater understanding of how Symbiodiniaceae functioning is shaped by their environments, thereby potentially providing a powerful – yet unexplored - tool to better understand the impact of future environmental change.

## Methods

### Culturing and growth conditions

Ten Symbiodiniaceae isolates (defined by internal transcribed spacer two (ITS2) designation) were sub-cultured from a long-term laboratory stock (over a decade in nutrient replete medium except for PVB18B which was in culture for ca. 3 years) maintained under nutrient replete conditions at the University of Technology Sydney (UTS). Isolates were from five genera (*Breviolum, Cladocopium, Durusdinium, Effrenium, Symbiodinium*) and eight species originating from a range of geographic locations, host organisms, and lifestyle (see Table 1) [2, 15]. Genotyping (using the ITS2 region) of the Symbiodiniaceae cultures to re-verify identity was conducted as per [ref. 51].

**Table 1:** Information on the Symbiodiniaceae origin (host isolate or free-living and geographic location) and internal transcribed spacer two (ITS2) major type profile. Culture isolate identification is also provided as found in the literature and as labelled internally at the University of Technology of Sydney (UTS)

Genus	Species	ITS2 Major type profile	Culture isolate identity	Internal isolate label	Geographic origin	Host isolate
*Symbiodinium*	*S. microadriaticum*	A1	CCMP2464, rt61	A1-61	Florida (Caribbean Sea)	*Cassiopeia xamachana* (jellyfish)
	*S. natans*	A3	CCMP2548, HA3-5, MBIC10, rt796	A-2548	Hawaii (Pacific)	Free-living
*Breviolum*	*B. minutum*	B1	B (UTS)	B1-UTS-B	S. Taiwan (Indo-Pacific)	*Euphyllia glabrescens* (coral)
	*B. pseudominutum*	B1	CCMP2463, rt12	B1-12	Puerto Rico (Caribbean Sea)	*Aiptasia tagetes* (sea anemone)
	*B. psygmophilum*	B2	PVB18B	PVB18B	Sydney Harbour (Pacific)	*Plesiastrea versipora* (coral)
*Cladocopium*	*C. goreaui*	C1	AIMS-aten-C1-MI-cfu-B2, SCF055	C1-Hetero-M	Magnetic Island (Pacific)	*Acropora tenuis* (coral)
	*C. goreaui*	C1	SCF055-06	SCF124	Magnetic Island (Pacific)	*Acropora tenuis* (coral)
	*C. goreaui*	C1	SCF058-04	SCF123	Magnetic Island (Pacific)	*Acropora millepora* (coral)
*Durusdinium*	*D. trenchii*	D1a	amur-D-MI, UTS D, (UTS_D)	SCF082	Magnetic Island (Pacific)	*Acropora muricata* (coral)
*Effrenium*	*E. voratum*	E	CCMP3420	E-3420	Santa Barbara (California)	Free-living

**Table 2:** Results of the redundancy analysis (RDA) to see which Symbiodiniaceae traits are predictive of the elementome. 9999 permutations were run. Variance inflation factors (VIF) were used to assess for multicollinearity. * denotes a significant p value at the 95% confidence interval. The explanatory factors included in the RDA were: absorption coefficient for the PSII light harvesting complex (σLHCII), electron transfer capacity from plastoquinone pigment PQA to plastocyanin (τ_1_), the plastoquinone re-oxidation rate (τ_2_), the plastoquinone re-oxidation rate normalised by the oxidised portion of the plastoquinone pool (τ_2_/PQ_OX_), photochemical dissipation (1-C), dynamic non-photochemical dissipation (1-Q), growth rate, and cell volume

Explanatory factor	DF	Inertia	*F*	*p*	VIF
σ_LHCII_	1	0.292	0.688	0.686	4.95
τ_1_	1	0.438	1.031	0.405	5.20
τ_2_	1	0.759	1.788	0.120	2.15
τ_2_/PQ_OX_	1	0.359	0.845	0.536	7.94
1-C	1	1.261	2.972	0.004*	2.51
1-Q	1	0.749	1.766	0.091	2.44
Growth rate	1	0.364	0.860	0.552	1.55
Cell volume	1	1.292	3.045	0.004*	2.98
Residual	20	8.486			

Isolates were cultured in sterile 0.2 μm filtered artificial seawater enriched with Daigo’s IMK medium (Nihon Pharmaceutical, Tokyo, Japan) [84] to assess intercellular elemental quota differences across isolates when using a common nutrient replete culturing medium. A literature search using key terms “temperature”, “heat stress”, “thermal stress”, “thermal performance” and “stress response”, of “Symbiodiniaceae”, “*Symbiodinium*” and “Zooxanthellae” between 1/01/1980-to-present (23/05/2020) returned 1,379 papers. Of the studies that looked at *ex situ* culturing of Symbiodiniaceae and reported culturing conditions, 42% used nutrient replete medium (IMK, F2, ASP-8A), demonstrating that Symbiodiniaceae are frequently grown in luxury uptake conditions.

Initial elemental concentrations of the culture medium (filtered artificial seawater enriched with Daigo’s IMK medium) were determined by ICP-MS and are provided in SI Table 13. IMK medium also contained thiamine, biotin, and vitamin B_12_ (753, 6, 1 nM receptively), as well as 33 nM ethylenediaminetetraacetic acid (EDTA) to regulate elemental bioavailability [85, 41]. Culturing was undertaken in a clean bench (class 100 laminar flow) [86], and plasticware washed following the method of [ref. 44]. Cultures were maintained in 300 mL tissue culture flasks (Corning, NY, USA), grown in an incubator under a temperature (mean ± standard error, S.E.) of 27.4 ± 0.4 °C measured using a HOBO Pendant® data logger set to log every 30 min. Light was provided by fluorescent tubes (Philips cool white, 4000 K) with a downwelling irradiance of 150.0 ± 4.1 μmol photons m^-1^s1 on a 12:12h light:dark cycle, with intensity measured using a 4π LI-190SA Quantum Sensor (LI-COR, Lincoln, NE, USA). Four of the cultures (*Symbiodinium microadriaticum* CCMP2464, *Effrenium voratum* CCMP3420, *Durusdinium trenchii* SCF082 and *Cladocopium goreaui* SCF058-04) identified to have different photophysiological traits (see Fig. 1) were also grown under 30.7 ± 0.01 °C to assess the impact of a different temperature regime on their elementome. However, *C. goreaui* (SCF058-04) did not grow at this temperature and was therefore omitted from analysis.

Cultures (triplicates per isolate) were maintained in steady state growth phase through regular dilutions to prevent them from becoming optically thick or nutrient starved [51]. All analyses were undertaken with cells in the exponential growth phase and each culture was grown for at least eight generations before sampling [2].

### Growth and cell size

Growth was determined from cell quantification throughout culture monitoring to define the exponential division rate per day (μ, d^-1^) as per [ref. 11]. Cell quantification was determined using a haemocytometer (Neubauer Haemocytometer, Fisher Scientific, Loughborough, UK) as per [ref. 87]. Average cell volume for each isolate was determined from a minimum of 50 cells from images captured using NIS-Elements AR software (v.4.30.000) and an Eclipse Ni-U optical microscope coupled with a DS-Fi2 colour camera (Nikon, Tokyo, Japan). Images were processed on the NIS-Elements AR software to calculate spherical volume from cell diameter, of all cells present in each image. Elemental measurements were subsequently considered relative to both division rate and volume as both can influence nutrient acquisition [65].

### FRRf measurements

On the days of elemental analyses, a 2 mL aliquot of culture was also collected for Light Induced Fluorescence Transient-Fast Repetition Rate fluorometry (LIFT-FRRf; Soliense Inc.) to obtain a suite of targeted photo-physiological parameters that have previously been identified as potential drivers of Symbiodiniaceae functional performance [2, 11]. To assess photosynthetic performance of the Symbiodiniaceae cultures, LIFT-FRRf parameters were selected that have previously been shown to govern functional differences amongst Symbiodiniaceae [2], including: maximum photochemical efficiency (*F*_*v*_*/F*_*m*_), absorption coefficient for the PSII light harvesting complex (σ_LHCII_), electron transfer capacity from plastoquinone pigment PQ_A_ to plastocyanin (τ_1_), the plastoquinone re-oxidation rate (τ_2_), the plastoquinone re-oxidation rate normalised by the oxidised portion of the plastoquinone pool (τ_2_/PQ_OX_), photochemical dissipation (1-C), and dynamic non-photochemical dissipation (1-Q). All parameters except for 1-C and 1-Q were collected in darkness. Values for 1-C and 1-Q were from 750 μmol photons m^-2^s^-1^. Calculation of all parameters are provided in SI.

### Elemental analysis

Aliquots of 100 mL and 150 mL from each replicate were pelleted for each isolate for trace elemental analysis and for total C and N analysis, respectively. The supernatant was discarded, and the pellet washed three times in TRIS buffer prior to being freeze dried and weighed. ICP-MS analysis on the wash steps (see Method below) verified that three wash steps has cleaned the cells of excess medium and externally sorbed ions, and thus, reported values represent the intercellular metal quotas. At the time of elemental sample collection, 1 mL of culture was collected to determine cell density, and 2 mL for photobiological parameter characterisation (as described above). Samples (approximately 40 mg of pellet) were analysed for total C and N by dry combustion with a Trumac® CN-analyser (Leco® Castle Hill, Australia) following manufacturer methods for soil and plant material with a furnace temperature at 1200 °C and a soil calibration standard.

Remaining elements were analysed via ICP-MS/(MS). Approximate 12 mg of the pellet were digested with a mixture of 100 μL of HNO_3_ (67-69% w/w, Choice Analytical, Australia) and 100 μL of H_2_O_2_ (30-32% w/w, Seastar Chemicals, Canada) and incubated overnight. Samples were subsequently diluted to a final volume of 2 mL with Milli Q water (18.2 MΩ; Merk Millipore) and filtered using 0.2 μm syringe filters (Captiva Econofilters, Agilent Technologies, Australia). Sample containers were polypropylene to avoid adsorption effects. High purity ICP-MS standard calibration solutions for external calibration (Choice Analytical, Australia) were diluted in aqueous solution of 3.3% HNO_3_ and 1.5% H_2_O_2_. Procedural blank samples (TRIS) were also run to check for potential contamination in the methodological process and came back negligible (< 1%). Quantitative analysis of elements was carried out using flow injection analysis (FIA) employing an Agilent 1200-Series HPLC system coupled with an 8900-series ICP-MS/MS instrument (Agilent Technologies, Australia). The interface was equipped with s-lenses and Pt sampler and skimmer cones. The ICP-MS/MS instrument was operated in MS/MS mode using oxygen as cell gas (BOC, 99.995%, grade 4.5, Australia). Performance of the ICP-MS instrument was tuned daily with a solution containing 1μgL^-1^ Li, Y, Tl and Ce to optimise sensitivity. The elements S and P are commonly interfered in ICP-MS and were therefore analysed via mass shifting (^31^P➔^31^P^16^O and ^32^S➔^32^S^16^O). Targeted isotopes are listed in SI Table 14. Limits of detection were calculated according to the 3-sigma criterion.

### Data analysis

Elemental concentrations were correlated against cell volume (arcsine transformed) and growth rate to examine for allometric and growth dependent elemental scaling. All subsequent analyses were conducted on element-to-phosphorous (E:P) ratios, following Redfield ratios [43] and because P is analysed simultaneously with the micronutrients [22, 27, 41, 43]. Macronutrient E:P is in mol:mol P^-1^ while micronutrient E:P is in mmol:mol P^-1^. E:P ratios were transformed to homogenise variance, and herein E:P refers to log-transformed data.

Six data analysis steps were employed: 1) Differences in traits (cell volume, division rate, σ_LHCII_, τ_1_, τ_2_, τ_2_/PQ_OX_, 1-C, 1-Q) between Symbiodiniaceae isolates was assessed by Analysis of Variance (ANOVA) with post-hoc Tukey, or Kruskal-Wallis test with post-hoc Dunn, depending on whether parametric test assumptions were fulfilled. Levene’s test was applied to assess for equal variance, and Shapiro-Wilk for normality combined with manual inspections of QQ-plots of the model residuals. Cell size, division rates, and photobiological traits were transformed to homogenise variance based on the bestNormalise package in R (Transformations in SI Table 15) [88]; 2) Elementomes amongst the 10 Symbiodiniaceae isolates was assessed using a principal component analysis (PCA) on zero-mean standardised E:P values, and the extracted ordination axes (PC1 and PC2) were compared across isolates using ANOVA and post-hoc Tukey. One replicate for SCF055-06 was omitted from the PCA since the C and N sample was lost. PCA loadings of E:P (visualised as vectors) were scaled to PCA eigenvalues; 3) Redundancy analysis (RDA) was next used to assess which Symbiodiniaceae traits are predictive of the elementome. Multicollinearity between traits was assessed through variance inflation scores [89]; 4) We then assessed whether variability in nutrient content decreased proportionately with cell mass [29]. The multiplicative range of log E:P (maximum E:P variability) was determined by calculating the maximum x minimum^-1^ for each E:P over all Symbiodiniaceae isolates and temperature treatments and correlating this to log-median E:P (function of total contribution to cellular biomass) [22]. Zero values were removed from the analysis; 5) Differences in E:P ratios across isolates were examined by ANOVA with post-hoc Tukey with Holm’s sequential correction; and finally, 6) The influence of an elevated temperature regime on the elementome of isolates CCMP2464, CCMP3420, and SCF082 was examined using PCA as described above. Differences in E:P ratios under temperature were compared with a two-way ANOVA with post-hoc Tukey. All data analysis was conducted in R (version 4), and code to reproduce these analyses are available online (https://github.com/nitschkematthew/Elementome_Symbiodiniaceae).

## Supplementary Information

**Additional file 1.**

## Data Availability

Data generated or analysed during this study are included in this published article and its supplementary information files or can be obtained from the corresponding author on reasonable request. All raw sequence data are accessible under NCBI’s BioProject (https://www.ncbi.nlm.nih.gov/bioproject/PRJNA812073).
