# Supplementary material for: Micronutrient content drives elementome variability amongst the Symbiodiniaceae
Source: BMC Plant Biol. 2022 Apr 9;22:184. doi: 10.1186/s12870-022-03512-0 (PMC8994382; doi:10.1186/s12870-022-03512-0)
Supplement: Supplementary file 1 — Additional file 1. [file 12870_2022_3512_MOESM1_ESM.docx]

**Supplementary Information**

**Micronutrient content drives elementome variability amongst the Symbiodiniaceae**

Emma F. Camp^1*^, Matthew R. Nitschke^1,2^, David Clases^3,4^, Raquel Gonzalez de Vega^3,4^, Hannah G. Reich^5^, Samantha Goyen^1^, David J. Suggett^1^

1 Climate Change Cluster, University of Technology Sydney, Broadway 2007, NSW, Australia

2 School of Biological Sciences, Victoria University of Wellington, 6012, New Zealand

3 The Atomic Medicine Initiative, University of Technology Sydney, 15 Broadway, Ultimo NSW 2007, Australia

4 Institute for Chemistry, University of Graz, Austria

5 Department of Biological Sciences, University of Rhode Island, 120 Flagg Road, Kingston RI 02881, USA

*Corresponding Author: emma.camp@uts.edu.au; +61 2 9514 1253

**SI Methods**

*FRRf measurements -* All aliquots were low-light (ca. 5 mmol photons m^-2^ s^-1^) acclimated for 30 min prior to measurement. The aliquot was used to fill the LIFT-FRRf optical chamber, and a steady state Fluorescence Light Curve (FLC) protocol commenced following an initial dark measurement. The light protocol was delivered by a 450 nm LED at steps of increasing irradiance (0, 10, 25, 50, 100, 150, 250, 500, 750 mmol photons m^-2^ s^-1^), with each step lasting 5 min. A series of flashlets that characterise the saturation and relaxation of the primary quinone acceptor of the PSII reaction centre, Q_A_ (Osmond *et al*., 2017, 2019) were measured at regular time intervals during each light step. Measurements were inspected across all isolates to verify the maximum time to reach steady state (see SI Fig. 8), after which, the next 20 acquisitions were averaged (see Suggett *et al*., 2015). Custom-designed software was used for all LIFT-FRRf programming and model fitting (Kolber et al. 1998; Osmond *et al*., 2017, 2019). All measurements were corrected for baseline fluorescence by blank analysis that consisted of the culture supernatant obtained from gentle filtration of the culture through a 0.2 µm syringe filter (Minsart NML, Sartorious, Germany).

Photobiological trait calculations followed the descriptions of Suggett et al. (2015). Definitions and calculations are shown below:

- The primary quinone acceptor of the PSII reaction centre (RCII), Q_A_
- Fluorescence from minimum to maximum (*F*_0_ to *F*_m_, *F*´ to *F*_m_´; in darkness, under actinic light, respectively).
- The PSII effective absorption cross section (σ_PSII_, σ_PSII_´)
- RCII ‘connectivity’ (ρ , ρ´)
- Absorption coefficient for the PSII light harvesting complex, *a*_LHCII_ ([σ_PSII_ · *n*] · [*F*_v_/*F*_m_]^-1^)
- τ_1_ and τ_2_, represent maximum electron transfer capacity from Q_A_ to plastoquinone (PQ) and for PQ pool re-oxidation, respectively.
- Normalising τ_2_ by the oxidised portion of the PQ pool (PQ_OX_) gives τ_2_/PQ_OX_.
- σ_LHCII_ (PSII effective cross section [= σ_PSII_/(*F*_v_/*F*_m_)])
- Photochemical ([1-C]) and dynamic non-photochemical (downregulated, [1-Q]) energy dissipation:

[1-C] = [*F*_m_´ - *F*´]/[*F*_m_´ - *F*_0_´] [1-Q] = ([*F*_m_´ - *F*_0_´]/*F*_m_´)/(*F*_v_/*F*_m_) *F*_0_´ = *F*_0_/[(*F*_v_/*F*_m_) + (*F*_0_/*F*_m_´)]

**Figures**


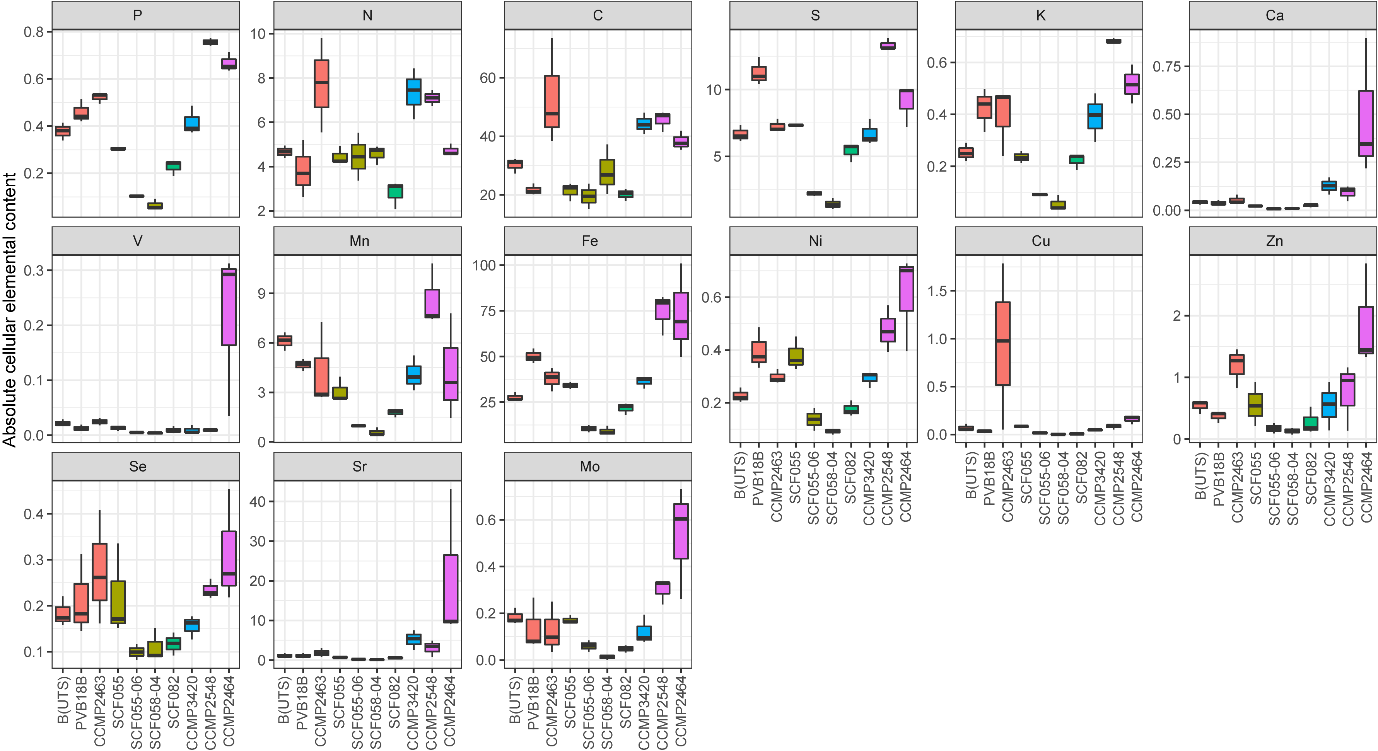


**SI Fig. 1** **Absolute intracellular elemental content of the ten Symbiodiniaceae isolates at 27.4°C**. Data is pg cell^-1^ for macronutrients (C, N, S, K and Ca) and fg cell^-1^ for micronutrients (V, Mn, Fe, Ni, Cu, Zn, Se, S, Mo). Lower and upper hinges of each boxplot correspond to the first and third quartiles, and 50% to the median. The upper and lower whiskers of each boxplot extend from the hinge to the largest and smallest value up to 1.5 × the inter-quartile range, respectively.


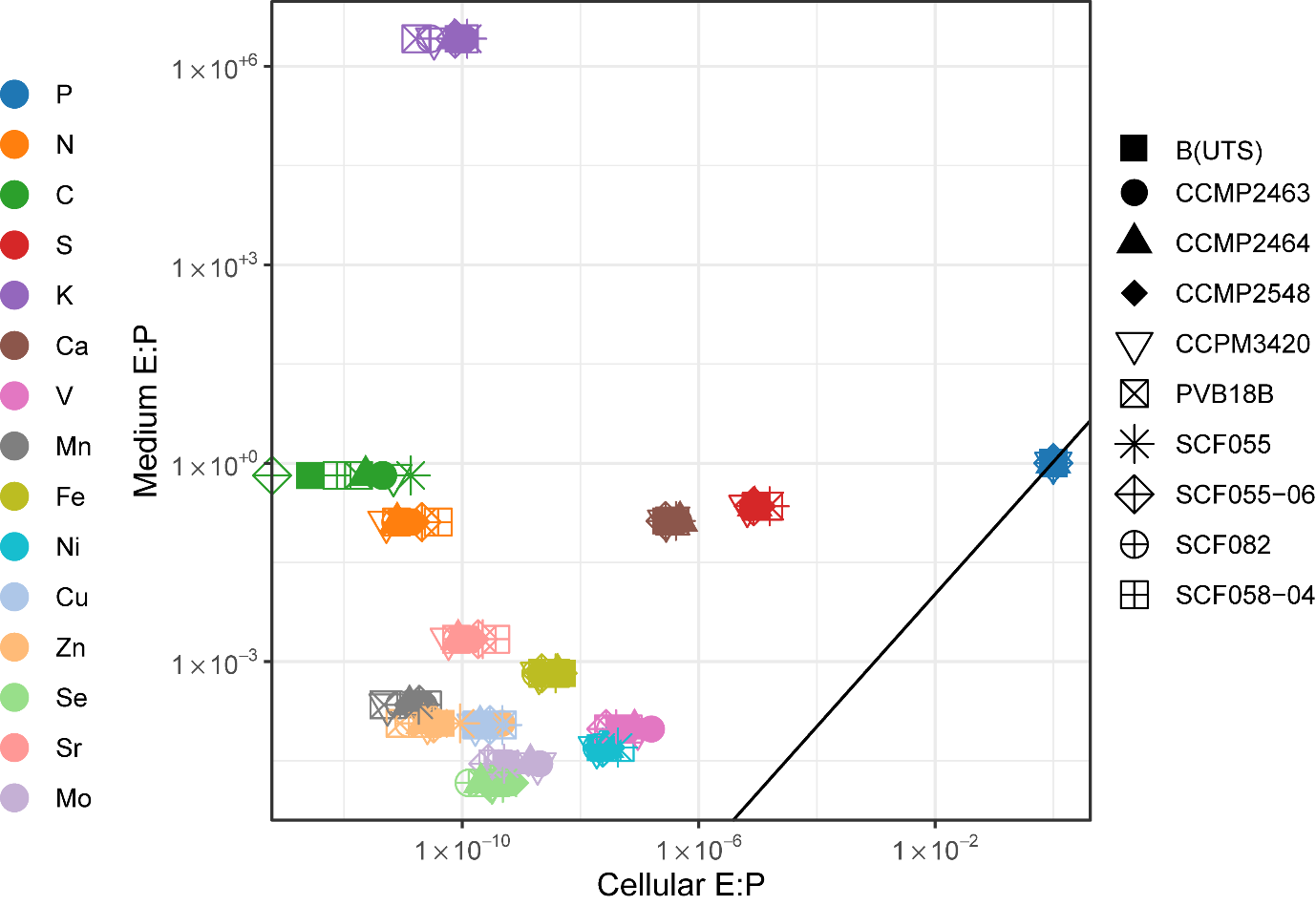


**SI Fig. 2 Comparison between the filtered artificial seawater enriched with Daigo’s IMK medium elemental content and Symbiodiniaceae cellular elemental quotas.** Both medium and cellular content is plotted as elemental ratios normalised to phosphorus (E:P). The black line represents a 1:1 relationship. Shapes denote the ten Symbiodiniaceae isolates, and colour represents the elements. Mean values are plotted (*n*= 3 per isolate) for 27.4°C.


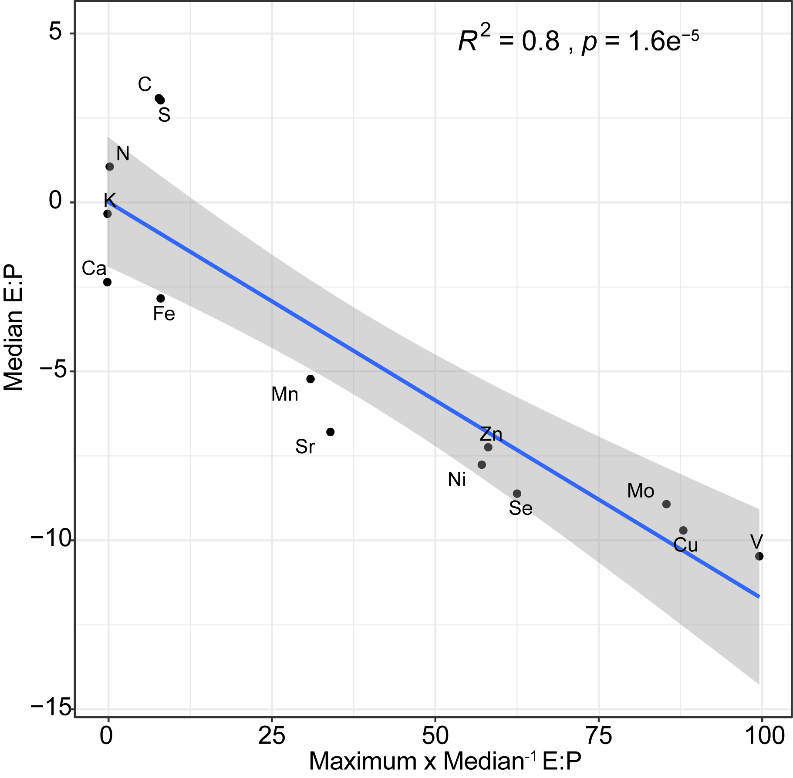


**SI Fig. 3** **Variability in (maximum x minimum^-1^) in element normalised to phosphorous (E:P) as a function of total contribution to cellular biomass (median E:P).** Data is in mol:mol for macronutrients (N, C, S, K and Ca) and mmol:mol for micronutrients (V, Mn, Fe, Ni, Cu, Zn, Se, S, Mo). Grey shading represents the 95% confidence interval.


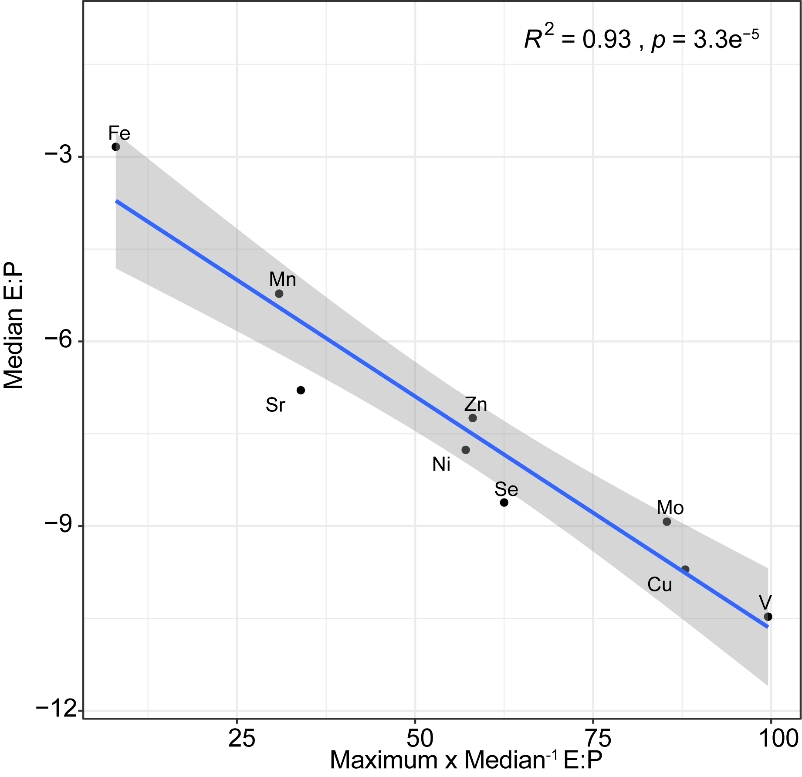


**SI Fig. 4 Variability in (maximum x minimum^-1^) element normalised to phosphorous (E:P) as a function of total contribution to cellular biomass (median E:P).** Data is in mmol:mol for micronutrients (V, Mn, Fe, Ni, Cu, Zn, Se, S, Mo). Grey shading represents the 95% confidence interval.


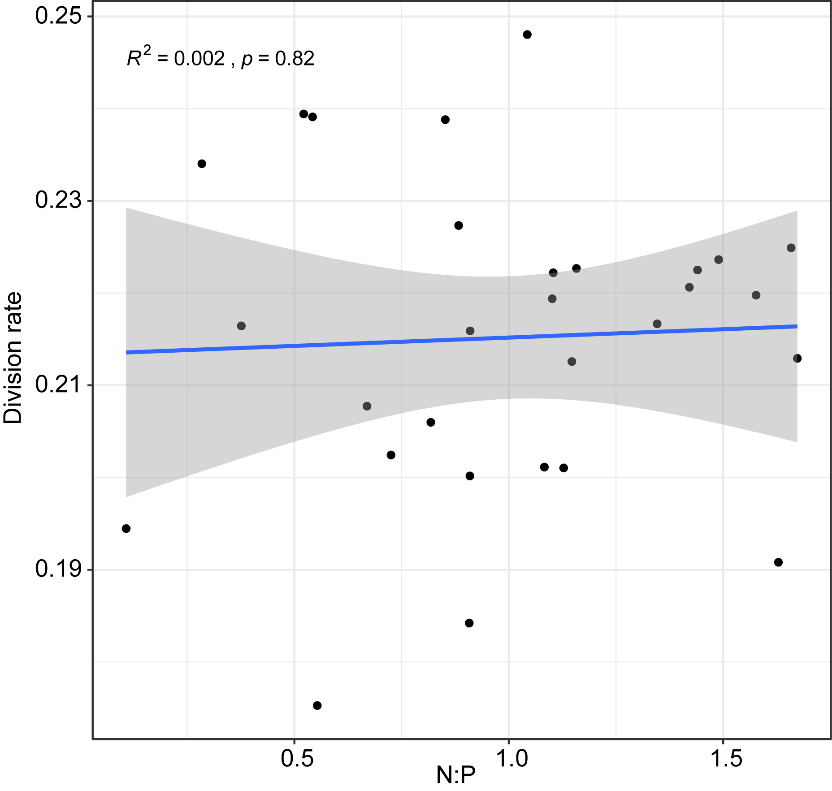


**SI Fig. 5** **Correlation of N:P log transformed with division rate for the ten Symbiodiniaceae isolates at 27.4°C**. Three replicates per isolate.


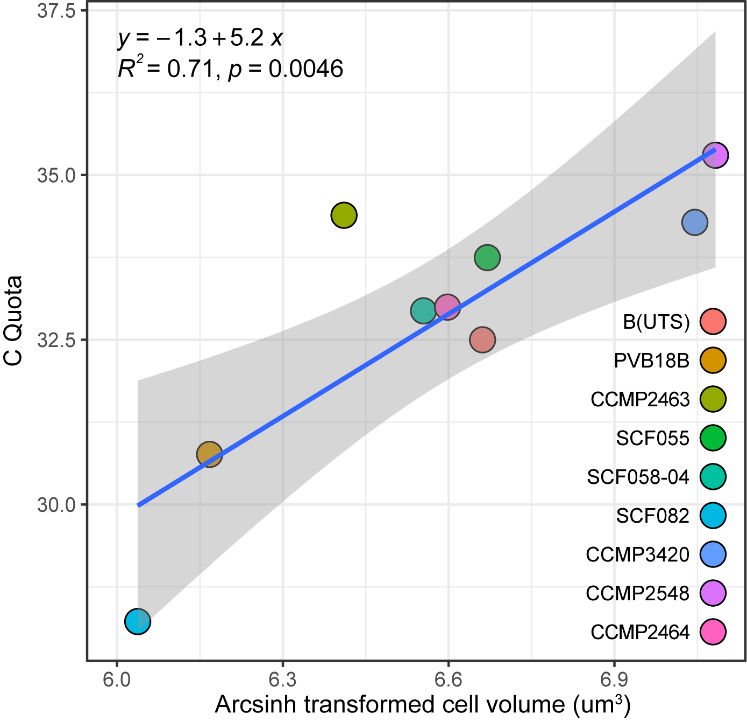


**SI Fig. 6 Correlation of absolute cellular C content with arcsine transformed cell volume (data is mean, *n*= 3) for the nine Symbiodiniaceae isolates (SCF055-06 was removed) at 27.4°C**. Blue line is the linear relationship, and grey area the 95% confidence band.

**SI Fig. 7 Within species comparisons of Symbiodiniaceae elementomes for five species across two studies.** Metal data is normalised to phosphorus (mmol:mol). Upper and lower hinges of each box correspond to the first and third quartiles (respectively) whereas whisker length corresponds to 1.5*inter-quartile range


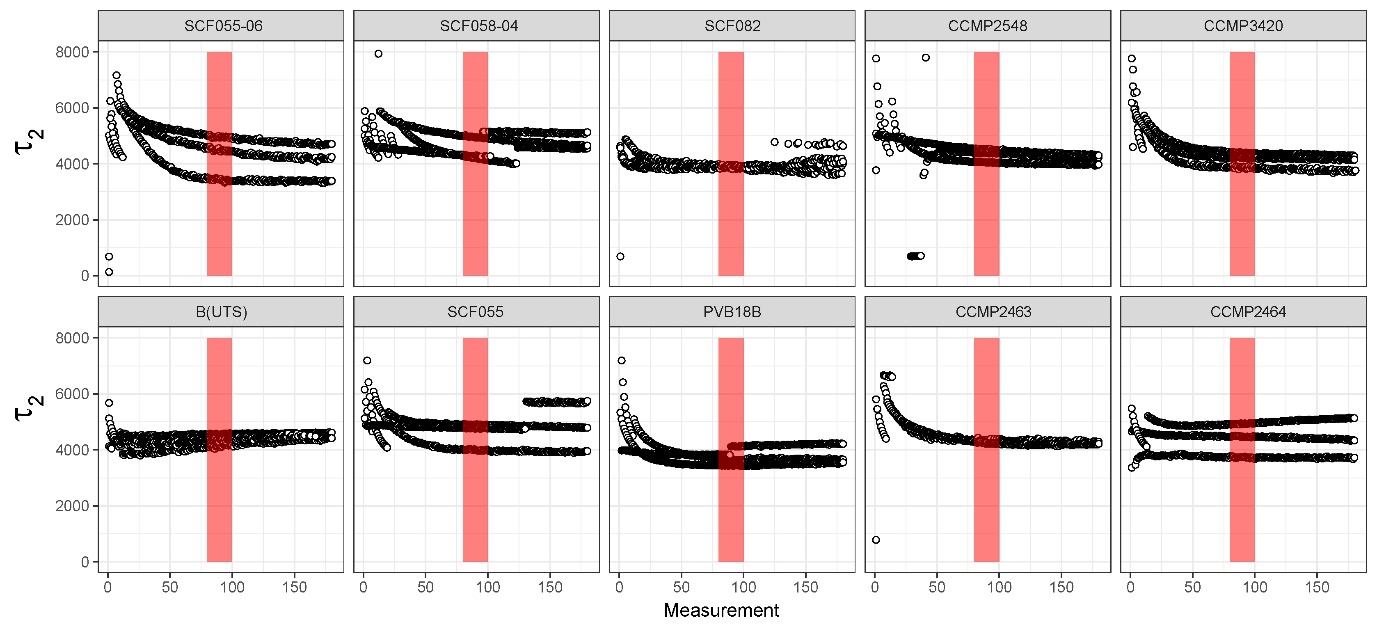


**SI Fig. 8** Example (τ_2_ measured under darkness) of how isolates vary in the duration required to reach steady state. Red area indicates the 20 acquisitions which were averaged for further analysis. This window is the same for all parameters analysed.

**Tables**

**SI Table 1** **Differences in traits (cell volume, growth rate, σ_LHCII_, τ_1_, τ_2_, τ_2_/PQ_OX_, 1-C, 1-Q) between Symbiodiniaceae isolates.** Differences were assessed by Analysis of Variance (ANOVA) with post hoc Tukey test, or Kruskal-Wallis test with post hoc Dunn, depending on whether parametric test assumptions were fulfilled. Levene’s test was applied to assess for equal variance, and Shapiro-Wilk test for normality combined with manual inspections of QQ-plots of the model residuals. For ANOVA, DFn is the degrees of freedom numerator, Dfd is the degrees of freedom denominator and ges is the generalised eta squared that is a useful metric for evaluating the size of an effect (>.26 considered large; Bakeman, 2005). For Kruskal-Wallis, df is degrees of freedom. Ns *p* > 0.05, * *p* ≤ 0.05 ** *p* ≤ 0.01 *** *p* ≤ 0.001.

1. ANOVA and Kruskal-Wallis

| Division Rate (ANOVA) | |  |  |  |  |  |
| --- | --- | --- | --- | --- | --- | --- |
| Effect | DFn | DFd | *F* | *p* | *p* <.05 | ges |
| Isolate ID | 9 | 20 | 3.669 | 0.007 | * | 0.623 |
| Cell Volume (ANOVA) | |  |  |  |  |  |
| Effect | DFn | DFd | *F* | *p* | *p* <.05 | ges |
| Isolate ID | 9 | 20 | 23.272 | 1.04E-08 | * | 0.913 |
| σLHCII (ANOVA) | |  |  |  |  |  |
| Effect | DFn | DFd | *F* | *p* | *p* <.05 | ges |
| Isolate ID | 9 | 20 | 10.631 | 7.19E-06 | * | 0.827 |
| τ_1_ (ANOVA) | |  |  |  |  |  |
| Effect | DFn | DFd | *F* | *p* | *p* <.05 | ges |
| Isolate ID | 9 | 20 | 1.735 | 0.146 |  | 0.438 |
| τ_2_ (ANOVA) | |  |  |  |  |  |
| Effect | DFn | DFd | *F* | *p* | *p* <.05 | ges |
| Isolate ID | 9 | 20 | 1.133 | 0.386 |  | 0.338 |
| τ_2/_PQox (ANOVA) | |  |  |  |  |  |
| Effect | DFn | DFd | *F* | *p* | *p* <.05 | ges |
| Isolate ID | 9 | 20 | 7.449 | 9.91E-05 | * | 0.77 |
| 1-C (Kruskal Wallis) | |  |  |  |  |  |
| .y. | n | statistic | df | p | method |  |
| 1-C | 30 | 16.06237 | 9 | 0.0656 | Kruskal-Wallis | |
| 1-Q (ANOVA) | |  |  |  |  |  |
| Effect | DFn | DFd | *F* | *p* | *p* <.05 | ges |
| Isolate ID | 9 | 20 | 10.81 | 6.31E-06 | * | 0.829 |
| *Fv/Fm* |  |  |  |  |  |  |
| Effect | DFn | DFd | *F* | *p* | *p* <.05 | ges |
| Isolate ID | 9 | 20 | 4.88 | 0.002 | * | 0.687 |

1. Post hoc Tukey test or Dunn

| Division rate |  |  |  |
| --- | --- | --- | --- |
| group1 | **group2** | ***p*.adj** | ***p*.adj.signif** |
| B(UTS) | PVB18B | 0.992 | ns |
| B(UTS) | CCMP2463 | 0.815 | ns |
| B(UTS) | SCF055 | 0.763 | ns |
| B(UTS) | SCF05506 | 0.833 | ns |
| B(UTS) | SCF05804 | 0.665 | ns |
| B(UTS) | SCF082 | 0.108 | ns |
| B(UTS) | CCMP3420 | 0.959 | ns |
| B(UTS) | CCMP2548 | 0.977 | ns |
| B(UTS) | CCMP2464 | 1 | ns |
| PVB18B | CCMP2463 | 0.999 | ns |
| PVB18B | SCF055 | 0.998 | ns |
| PVB18B | SCF05506 | 0.303 | ns |
| PVB18B | SCF05804 | 0.991 | ns |
| PVB18B | SCF082 | 0.486 | ns |
| PVB18B | CCMP3420 | 1 | ns |
| PVB18B | CCMP2548 | 1 | ns |
| PVB18B | CCMP2464 | 0.89 | ns |
| CCMP2463 | SCF055 | 1 | ns |
| CCMP2463 | SCF05506 | 0.091 | ns |
| CCMP2463 | SCF05804 | 1 | ns |
| CCMP2463 | SCF082 | 0.873 | ns |
| CCMP2463 | CCMP3420 | 1 | ns |
| CCMP2463 | CCMP2548 | 1 | ns |
| CCMP2463 | CCMP2464 | 0.511 | ns |
| SCF055 | SCF05506 | 0.0751 | ns |
| SCF055 | SCF05804 | 1 | ns |
| SCF055 | SCF082 | 0.91 | ns |
| SCF055 | CCMP3420 | 1 | ns |
| SCF055 | CCMP2548 | 1 | ns |
| SCF055 | CCMP2464 | 0.453 | ns |
| SCF05506 | SCF05804 | 0.0535 | ns |
| SCF05506 | SCF082 | 0.0038 | ** |
| SCF05506 | CCMP3420 | 0.193 | ns |
| SCF05506 | CCMP2548 | 0.233 | ns |
| SCF05506 | CCMP2464 | 0.982 | ns |
| SCF05804 | SCF082 | 0.956 | ns |
| SCF05804 | CCMP3420 | 0.999 | ns |
| SCF05804 | CCMP2548 | 0.998 | ns |
| SCF05804 | CCMP2464 | 0.36 | ns |
| SCF082 | CCMP3420 | 0.657 | ns |
| SCF082 | CCMP2548 | 0.587 | ns |
| SCF082 | CCMP2464 | 0.0385 | * |
| CCMP3420 | CCMP2548 | 1 | ns |
| CCMP3420 | CCMP2464 | 0.757 | ns |
| CCMP2548 | CCMP2464 | 0.817 | ns |
| Cell volume |  |  |  |
| group1 | **group2** | ***p*.adj** | ***p*.adj.signif** |
| B(UTS) | PVB18B | 0.00284 | ** |
| B(UTS) | CCMP2463 | 0.336 | ns |
| B(UTS) | SCF055 | 1 | ns |
| B(UTS) | SCF05506 | 0.376 | ns |
| B(UTS) | SCF05804 | 0.984 | ns |
| B(UTS) | SCF082 | 1.76E-04 | *** |
| B(UTS) | CCMP3420 | 0.0296 | * |
| B(UTS) | CCMP2548 | 0.0135 | * |
| B(UTS) | CCMP2464 | 1 | ns |
| PVB18B | CCMP2463 | 0.373 | ns |
| PVB18B | SCF055 | 0.00235 | ** |
| PVB18B | SCF05506 | 1.77E-05 | **** |
| PVB18B | SCF05804 | 0.0278 | * |
| PVB18B | SCF082 | 0.946 | ns |
| PVB18B | CCMP3420 | 1.23E-06 | **** |
| PVB18B | CCMP2548 | 6.32E-07 | **** |
| PVB18B | CCMP2464 | 0.0111 | * |
| CCMP2463 | SCF055 | 0.296 | ns |
| CCMP2463 | SCF05506 | 0.00287 | ** |
| CCMP2463 | SCF05804 | 0.906 | ns |
| CCMP2463 | SCF082 | 0.0369 | * |
| CCMP2463 | CCMP3420 | 1.40E-04 | *** |
| CCMP2463 | CCMP2548 | 6.48E-05 | **** |
| CCMP2463 | CCMP2464 | 0.698 | ns |
| SCF055 | SCF05506 | 0.423 | ns |
| SCF055 | SCF05804 | 0.974 | ns |
| SCF055 | SCF082 | 1.46E-04 | *** |
| SCF055 | CCMP3420 | 0.0354 | * |
| SCF055 | CCMP2548 | 0.0162 | * |
| SCF055 | CCMP2464 | 0.999 | ns |
| SCF05506 | SCF05804 | 0.0596 | ns |
| SCF05506 | SCF082 | 1.51E-06 | **** |
| SCF05506 | CCMP3420 | 0.914 | ns |
| SCF05506 | CCMP2548 | 0.747 | ns |
| SCF05506 | CCMP2464 | 0.136 | ns |
| SCF05804 | SCF082 | 0.00171 | ** |
| SCF05804 | CCMP3420 | 0.00303 | ** |
| SCF05804 | CCMP2548 | 0.00135 | ** |
| SCF05804 | CCMP2464 | 1 | ns |
| SCF082 | CCMP3420 | 1.30E-07 | **** |
| SCF082 | CCMP2548 | 7.06E-08 | **** |
| SCF082 | CCMP2464 | 6.72E-04 | *** |
| CCMP3420 | CCMP2548 | 1 | ns |
| CCMP3420 | CCMP2464 | 0.00774 | ** |
| CCMP2548 | CCMP2464 | 0.00346 | ** |
| 1-C |  |  |  |
| group1 | **group2** | ***p*.adj** | ***p*.adj.signif** |
| B(UTS) | PVB18B | 1 | ns |
| B(UTS) | CCMP2463 | 0.473215 | ns |
| B(UTS) | SCF055 | 1 | ns |
| B(UTS) | SCF05506 | 1 | ns |
| B(UTS) | SCF05804 | 1 | ns |
| B(UTS) | SCF082 | 1 | ns |
| B(UTS) | CCMP3420 | 0.321837 | ns |
| B(UTS) | CCMP2548 | 1 | ns |
| B(UTS) | CCMP2464 | 1 | ns |
| PVB18B | CCMP2463 | 1 | ns |
| PVB18B | SCF055 | 1 | ns |
| PVB18B | SCF05506 | 1 | ns |
| PVB18B | SCF05804 | 1 | ns |
| PVB18B | SCF082 | 1 | ns |
| PVB18B | CCMP3420 | 1 | ns |
| PVB18B | CCMP2548 | 1 | ns |
| PVB18B | CCMP2464 | 1 | ns |
| CCMP2463 | SCF055 | 1 | ns |
| CCMP2463 | SCF05506 | 1 | ns |
| CCMP2463 | SCF05804 | 1 | ns |
| CCMP2463 | SCF082 | 1 | ns |
| CCMP2463 | CCMP3420 | 1 | ns |
| CCMP2463 | CCMP2548 | 1 | ns |
| CCMP2463 | CCMP2464 | 1 | ns |
| SCF055 | SCF05506 | 1 | ns |
| SCF055 | SCF05804 | 1 | ns |
| SCF055 | SCF082 | 1 | ns |
| SCF055 | CCMP3420 | 1 | ns |
| SCF055 | CCMP2548 | 1 | ns |
| SCF055 | CCMP2464 | 1 | ns |
| SCF05506 | SCF05804 | 1 | ns |
| SCF05506 | SCF082 | 1 | ns |
| SCF05506 | CCMP3420 | 0.857288 | ns |
| SCF05506 | CCMP2548 | 1 | ns |
| SCF05506 | CCMP2464 | 1 | ns |
| SCF05804 | SCF082 | 1 | ns |
| SCF05804 | CCMP3420 | 0.77515 | ns |
| SCF05804 | CCMP2548 | 1 | ns |
| SCF05804 | CCMP2464 | 1 | ns |
| SCF082 | CCMP3420 | 1 | ns |
| SCF082 | CCMP2548 | 1 | ns |
| SCF082 | CCMP2464 | 1 | ns |
| CCMP3420 | CCMP2548 | 1 | ns |
| CCMP3420 | CCMP2464 | 1 | ns |
| CCMP2548 | CCMP2464 | 1 | ns |
| 1-Q |  |  |  |
| group1 | **group2** | ***p*.adj** | ***p*.adj.signif** |
| B(UTS) | PVB18B | 0.718 | ns |
| B(UTS) | CCMP2463 | 1.06E-04 | *** |
| B(UTS) | SCF055 | 0.137 | ns |
| B(UTS) | SCF05506 | 0.67 | ns |
| B(UTS) | SCF05804 | 0.35 | ns |
| B(UTS) | SCF082 | 1 | ns |
| B(UTS) | CCMP3420 | 4.21E-04 | *** |
| B(UTS) | CCMP2548 | 0.00156 | ** |
| B(UTS) | CCMP2464 | 0.00887 | ** |
| PVB18B | CCMP2463 | 0.00541 | ** |
| PVB18B | SCF055 | 0.964 | ns |
| PVB18B | SCF05506 | 1 | ns |
| PVB18B | SCF05804 | 1 | ns |
| PVB18B | SCF082 | 0.518 | ns |
| PVB18B | CCMP3420 | 0.0219 | * |
| PVB18B | CCMP2548 | 0.0752 | ns |
| PVB18B | CCMP2464 | 0.305 | ns |
| CCMP2463 | SCF055 | 0.0683 | ns |
| CCMP2463 | SCF05506 | 0.00647 | ** |
| CCMP2463 | SCF05804 | 0.0212 | * |
| CCMP2463 | SCF082 | 5.35E-05 | **** |
| CCMP2463 | CCMP3420 | 1 | ns |
| CCMP2463 | CCMP2548 | 0.954 | ns |
| CCMP2463 | CCMP2464 | 0.581 | ns |
| SCF055 | SCF05506 | 0.977 | ns |
| SCF055 | SCF05804 | 1 | ns |
| SCF055 | SCF082 | 0.0734 | ns |
| SCF055 | CCMP3420 | 0.223 | ns |
| SCF055 | CCMP2548 | 0.526 | ns |
| SCF055 | CCMP2464 | 0.931 | ns |
| SCF05506 | SCF05804 | 1 | ns |
| SCF05506 | SCF082 | 0.47 | ns |
| SCF05506 | CCMP3420 | 0.026 | * |
| SCF05506 | CCMP2548 | 0.0882 | ns |
| SCF05506 | CCMP2464 | 0.344 | ns |
| SCF05804 | SCF082 | 0.209 | ns |
| SCF05804 | CCMP3420 | 0.0796 | ns |
| SCF05804 | CCMP2548 | 0.236 | ns |
| SCF05804 | CCMP2464 | 0.663 | ns |
| SCF082 | CCMP3420 | 2.07E-04 | *** |
| SCF082 | CCMP2548 | 7.59E-04 | *** |
| SCF082 | CCMP2464 | 0.00431 | ** |
| CCMP3420 | CCMP2548 | 1 | ns |
| CCMP3420 | CCMP2464 | 0.914 | ns |
| CCMP2548 | CCMP2464 | 0.998 | ns |
| σLHCII |  |  |  |
| group1 | **group2** | ***p*.adj** | ***p*.adj.signif** |
| CCMP2548 | CCMP3420 | 0.0553 | ns |
| CCMP2548 | B(UTS) | 0.00125 | ** |
| CCMP2548 | SCF055 | 1 | ns |
| CCMP2548 | PVB18B | 0.307 | ns |
| CCMP2548 | CCMP2463 | 1 | ns |
| CCMP2548 | CCMP2464 | 1 | ns |
| CCMP2548 | SCF05506 | 1 | ns |
| CCMP2548 | SCF05804 | 1 | ns |
| CCMP2548 | SCF082 | 0.734 | ns |
| CCMP3420 | B(UTS) | 1.08E-06 | **** |
| CCMP3420 | SCF055 | 0.182 | ns |
| CCMP3420 | PVB18B | 2.35E-04 | *** |
| CCMP3420 | CCMP2463 | 0.073 | ns |
| CCMP3420 | CCMP2464 | 0.0161 | * |
| CCMP3420 | SCF05506 | 0.0723 | ns |
| CCMP3420 | SCF05804 | 0.161 | ns |
| CCMP3420 | SCF082 | 0.00118 | ** |
| B(UTS) | SCF055 | 3.17E-04 | *** |
| B(UTS) | PVB18B | 0.231 | ns |
| B(UTS) | CCMP2463 | 9.20E-04 | *** |
| B(UTS) | CCMP2464 | 0.00455 | ** |
| B(UTS) | SCF05506 | 0.00093 | *** |
| B(UTS) | SCF05804 | 3.69E-04 | *** |
| B(UTS) | SCF082 | 0.0582 | ns |
| SCF055 | PVB18B | 0.104 | ns |
| SCF055 | CCMP2463 | 1 | ns |
| SCF055 | CCMP2464 | 0.96 | ns |
| SCF055 | SCF05506 | 1 | ns |
| SCF055 | SCF05804 | 1 | ns |
| SCF055 | SCF082 | 0.364 | ns |
| PVB18B | CCMP2463 | 0.247 | ns |
| PVB18B | CCMP2464 | 0.641 | ns |
| PVB18B | SCF05506 | 0.249 | ns |
| PVB18B | SCF05804 | 0.118 | ns |
| PVB18B | SCF082 | 0.999 | ns |
| CCMP2463 | CCMP2464 | 0.999 | ns |
| CCMP2463 | SCF05506 | 1 | ns |
| CCMP2463 | SCF05804 | 1 | ns |
| CCMP2463 | SCF082 | 0.651 | ns |
| CCMP2464 | SCF05506 | 0.999 | ns |
| CCMP2464 | SCF05804 | 0.972 | ns |
| CCMP2464 | SCF082 | 0.964 | ns |
| SCF05506 | SCF05804 | 1 | ns |
| SCF05506 | SCF082 | 0.655 | ns |
| SCF05804 | SCF082 | 0.401 | ns |
| τ_1_ |  |  |  |
| group1 | **group2** | ***p*.adj** | ***p*.adj.signif** |
| CCMP2548 | CCMP3420 | 0.989 | ns |
| CCMP2548 | B(UTS) | 0.801 | ns |
| CCMP2548 | SCF055 | 1 | ns |
| CCMP2548 | PVB18B | 0.94 | ns |
| CCMP2548 | CCMP2463 | 0.944 | ns |
| CCMP2548 | CCMP2464 | 1 | ns |
| CCMP2548 | SCF05506 | 0.928 | ns |
| CCMP2548 | SCF05804 | 1 | ns |
| CCMP2548 | SCF082 | 1 | ns |
| CCMP3420 | B(UTS) | 0.251 | ns |
| CCMP3420 | SCF055 | 0.994 | ns |
| CCMP3420 | PVB18B | 1 | ns |
| CCMP3420 | CCMP2463 | 1 | ns |
| CCMP3420 | CCMP2464 | 0.964 | ns |
| CCMP3420 | SCF05506 | 1 | ns |
| CCMP3420 | SCF05804 | 1 | ns |
| CCMP3420 | SCF082 | 0.998 | ns |
| B(UTS) | SCF055 | 0.754 | ns |
| B(UTS) | PVB18B | 0.148 | ns |
| B(UTS) | CCMP2463 | 0.152 | ns |
| B(UTS) | CCMP2464 | 0.89 | ns |
| B(UTS) | SCF05506 | 0.137 | ns |
| B(UTS) | SCF05804 | 0.553 | ns |
| B(UTS) | SCF082 | 0.668 | ns |
| SCF055 | PVB18B | 0.96 | ns |
| SCF055 | CCMP2463 | 0.963 | ns |
| SCF055 | CCMP2464 | 1 | ns |
| SCF055 | SCF05506 | 0.951 | ns |
| SCF055 | SCF05804 | 1 | ns |
| SCF055 | SCF082 | 1 | ns |
| PVB18B | CCMP2463 | 1 | ns |
| PVB18B | CCMP2464 | 0.873 | ns |
| PVB18B | SCF05506 | 1 | ns |
| PVB18B | SCF05804 | 0.995 | ns |
| PVB18B | SCF082 | 0.982 | ns |
| CCMP2463 | CCMP2464 | 0.88 | ns |
| CCMP2463 | SCF05506 | 1 | ns |
| CCMP2463 | SCF05804 | 0.996 | ns |
| CCMP2463 | SCF082 | 0.984 | ns |
| CCMP2464 | SCF05506 | 0.855 | ns |
| CCMP2464 | SCF05804 | 1 | ns |
| CCMP2464 | SCF082 | 1 | ns |
| SCF05506 | SCF05804 | 0.993 | ns |
| SCF05506 | SCF082 | 0.977 | ns |
| SCF05804 | SCF082 | 1 | ns |
| τ_2_ |  |  |  |
| group1 | **group2** | ***p*.adj** | ***p*.adj.signif** |
| CCMP2548 | CCMP3420 | 1 | ns |
| CCMP2548 | B(UTS) | 1 | ns |
| CCMP2548 | SCF055 | 1 | ns |
| CCMP2548 | PVB18B | 0.756 | ns |
| CCMP2548 | CCMP2463 | 1 | ns |
| CCMP2548 | CCMP2464 | 1 | ns |
| CCMP2548 | SCF05506 | 1 | ns |
| CCMP2548 | SCF05804 | 1 | ns |
| CCMP2548 | SCF082 | 0.945 | ns |
| CCMP3420 | B(UTS) | 1 | ns |
| CCMP3420 | SCF055 | 0.995 | ns |
| CCMP3420 | PVB18B | 0.957 | ns |
| CCMP3420 | CCMP2463 | 0.971 | ns |
| CCMP3420 | CCMP2464 | 1 | ns |
| CCMP3420 | SCF05506 | 1 | ns |
| CCMP3420 | SCF05804 | 0.997 | ns |
| CCMP3420 | SCF082 | 0.998 | ns |
| B(UTS) | SCF055 | 1 | ns |
| B(UTS) | PVB18B | 0.749 | ns |
| B(UTS) | CCMP2463 | 1 | ns |
| B(UTS) | CCMP2464 | 1 | ns |
| B(UTS) | SCF05506 | 1 | ns |
| B(UTS) | SCF05804 | 1 | ns |
| B(UTS) | SCF082 | 0.942 | ns |
| SCF055 | PVB18B | 0.536 | ns |
| SCF055 | CCMP2463 | 1 | ns |
| SCF055 | CCMP2464 | 1 | ns |
| SCF055 | SCF05506 | 1 | ns |
| SCF055 | SCF05804 | 1 | ns |
| SCF055 | SCF082 | 0.807 | ns |
| PVB18B | CCMP2463 | 0.377 | ns |
| PVB18B | CCMP2464 | 0.78 | ns |
| PVB18B | SCF05506 | 0.859 | ns |
| PVB18B | SCF05804 | 0.556 | ns |
| PVB18B | SCF082 | 1 | ns |
| CCMP2463 | CCMP2464 | 0.999 | ns |
| CCMP2463 | SCF05506 | 0.996 | ns |
| CCMP2463 | SCF05804 | 1 | ns |
| CCMP2463 | SCF082 | 0.649 | ns |
| CCMP2464 | SCF05506 | 1 | ns |
| CCMP2464 | SCF05804 | 1 | ns |
| CCMP2464 | SCF082 | 0.955 | ns |
| SCF05506 | SCF05804 | 1 | ns |
| SCF05506 | SCF082 | 0.981 | ns |
| SCF05804 | SCF082 | 0.823 | ns |
| τ_2_/PQ_OX_ |  |  |  |
| group1 | **group2** | ***p*.adj** | ***p*.adj.signif** |
| CCMP2548 | CCMP3420 | 0.438 | ns |
| CCMP2548 | B(UTS) | 0.0303 | * |
| CCMP2548 | SCF055 | 1 | ns |
| CCMP2548 | PVB18B | 0.668 | ns |
| CCMP2548 | CCMP2463 | 0.866 | ns |
| CCMP2548 | CCMP2464 | 1 | ns |
| CCMP2548 | SCF05506 | 0.42 | ns |
| CCMP2548 | SCF05804 | 0.999 | ns |
| CCMP2548 | SCF082 | 0.929 | ns |
| CCMP3420 | B(UTS) | 2.16E-04 | *** |
| CCMP3420 | SCF055 | 0.653 | ns |
| CCMP3420 | PVB18B | 0.0129 | * |
| CCMP3420 | CCMP2463 | 0.999 | ns |
| CCMP3420 | CCMP2464 | 0.21 | ns |
| CCMP3420 | SCF05506 | 1 | ns |
| CCMP3420 | SCF05804 | 0.842 | ns |
| CCMP3420 | SCF082 | 0.0414 | * |
| B(UTS) | SCF055 | 0.0142 | * |
| B(UTS) | PVB18B | 0.68 | ns |
| B(UTS) | CCMP2463 | 0.00111 | ** |
| B(UTS) | CCMP2464 | 0.082 | ns |
| B(UTS) | SCF05506 | 2.02E-04 | *** |
| B(UTS) | SCF05804 | 0.0067 | ** |
| B(UTS) | SCF082 | 0.357 | ns |
| SCF055 | PVB18B | 0.453 | ns |
| SCF055 | CCMP2463 | 0.969 | ns |
| SCF055 | CCMP2464 | 0.996 | ns |
| SCF055 | SCF05506 | 0.633 | ns |
| SCF055 | SCF05804 | 1 | ns |
| SCF055 | SCF082 | 0.78 | ns |
| PVB18B | CCMP2463 | 0.0629 | ns |
| PVB18B | CCMP2464 | 0.911 | ns |
| PVB18B | SCF05506 | 0.012 | * |
| PVB18B | SCF05804 | 0.277 | ns |
| PVB18B | SCF082 | 1 | ns |
| CCMP2463 | CCMP2464 | 0.597 | ns |
| CCMP2463 | SCF05506 | 0.998 | ns |
| CCMP2463 | SCF05804 | 0.997 | ns |
| CCMP2463 | SCF082 | 0.177 | ns |
| CCMP2464 | SCF05506 | 0.199 | ns |
| CCMP2464 | SCF05804 | 0.965 | ns |
| CCMP2464 | SCF082 | 0.996 | ns |
| SCF05506 | SCF05804 | 0.827 | ns |
| SCF05506 | SCF082 | 0.0387 | * |
| SCF05804 | SCF082 | 0.576 | ns |
| *Fv/fm* |  |  |  |
| group1 | **group2** | ***p*.adj** | ***p*.adj.signif** |
| CCMP2548 | CCMP3420 | 0.657 | ns |
| CCMP2548 | CladeB | 0.442 | ns |
| CCMP2548 | HeteroM | 0.188 | ns |
| CCMP2548 | PVB18B | 1 | ns |
| CCMP2548 | RT12 | 0.996 | ns |
| CCMP2548 | RT61 | 1 | ns |
| CCMP2548 | SCF05506 | 0.532 | ns |
| CCMP2548 | SCF05804 | 0.589 | ns |
| CCMP2548 | UTSD | 0.511 | ns |
| CCMP3420 | CladeB | 0.0126 | * |
| CCMP3420 | HeteroM | 0.994 | ns |
| CCMP3420 | PVB18B | 0.53 | ns |
| CCMP3420 | RT12 | 0.983 | ns |
| CCMP3420 | RT61 | 0.712 | ns |
| CCMP3420 | SCF05506 | 1 | ns |
| CCMP3420 | SCF05804 | 1 | ns |
| CCMP3420 | UTSD | 1 | ns |
| CladeB | HeteroM | 0.00168 | ** |
| CladeB | PVB18B | 0.566 | ns |
| CladeB | RT12 | 0.111 | ns |
| CladeB | RT61 | 0.391 | ns |
| CladeB | SCF05506 | 0.00805 | ** |
| CladeB | SCF05804 | 0.00986 | ** |
| CladeB | UTSD | 0.00747 | ** |
| HeteroM | PVB18B | 0.129 | ns |
| HeteroM | RT12 | 0.616 | ns |
| HeteroM | RT61 | 0.219 | ns |
| HeteroM | SCF05506 | 0.999 | ns |
| HeteroM | SCF05804 | 0.998 | ns |
| HeteroM | UTSD | 0.999 | ns |
| PVB18B | RT12 | 0.983 | ns |
| PVB18B | RT61 | 1 | ns |
| PVB18B | SCF05506 | 0.411 | ns |
| PVB18B | SCF05804 | 0.463 | ns |
| PVB18B | UTSD | 0.392 | ns |
| RT12 | RT61 | 0.998 | ns |
| RT12 | SCF05506 | 0.949 | ns |
| RT12 | SCF05804 | 0.968 | ns |
| RT12 | UTSD | 0.941 | ns |
| RT61 | SCF05506 | 0.588 | ns |
| RT61 | SCF05804 | 0.645 | ns |
| RT61 | UTSD | 0.567 | ns |
| SCF05506 | SCF05804 | 1 | ns |
| SCF05506 | UTSD | 1 | ns |
| SCF05804 | UTSD | 1 | ns |

**SI Table 2 Assessment of differences in coordinates for Principal Component 1 (PC1) and 2 (PC2) for the Principal Component Analysis (PCA) of the 10 Symbiodiniaceae isolates at 27.4°C.** Differences were assessed via Analysis of Variance (ANOVA) with post hoc Tukey test. Levene’s test was applied to assess for equal variance, and Shapiro-Wilk for normality combined with manual inspections of QQ-plots of the model residuals. DFn is the degrees of freedom numerator, Dfd is the degrees of freedom denominator and ges is the generalised eta squared that is a useful metric for evaluating the size of an effect (>.26 considered large; Bakeman, 2005). Ns p > 0.05, * p ≤ 0.05 ** p ≤ 0.01 *** p ≤ 0.001.

ANOVA

| PC1 |  |  |  |  |  |  |
| --- | --- | --- | --- | --- | --- | --- |
| Effect | DFn | DFd | F | p | p <.05 | ges |
| culture_id | 9 | 19 | 9.485 | 2.36E-05 | * | 0.818 |
| PC2 |  |  |  |  |  |  |
| Effect | DFn | DFd | F | p | p <.05 | ges |
| culture_id | 9 | 19 | 3.706 | 0.008 | * | 0.637 |

Tukey post hoc test PC1

| group1 | group2 | p.adj | p.adj.signif |
| --- | --- | --- | --- |
| B(UTS) | PVB18B | 0.19 | ns |
| B(UTS) | CCMP2463 | 0.832 | ns |
| B(UTS) | SCF055 | 0.0513 | ns |
| B(UTS) | SCF05506 | 0.632 | ns |
| B(UTS) | SCF05804 | 0.00534 | ** |
| B(UTS) | SCF082 | 0.738 | ns |
| B(UTS) | CCMP3420 | 1 | ns |
| B(UTS) | CCMP2548 | 0.998 | ns |
| B(UTS) | CCMP2464 | 0.688 | ns |
| PVB18B | CCMP2463 | 0.00804 | ** |
| PVB18B | SCF055 | 0.999 | ns |
| PVB18B | SCF05506 | 1 | ns |
| PVB18B | SCF05804 | 0.731 | ns |
| PVB18B | SCF082 | 0.983 | ns |
| PVB18B | CCMP3420 | 0.0804 | ns |
| PVB18B | CCMP2548 | 0.587 | ns |
| PVB18B | CCMP2464 | 0.00457 | ** |
| CCMP2463 | SCF055 | 0.00179 | ** |
| CCMP2463 | SCF05506 | 0.0693 | ns |
| CCMP2463 | SCF05804 | 1.85E-04 | *** |
| CCMP2463 | SCF082 | 0.0708 | ns |
| CCMP2463 | CCMP3420 | 0.975 | ns |
| CCMP2463 | CCMP2548 | 0.372 | ns |
| CCMP2463 | CCMP2464 | 1 | ns |
| SCF055 | SCF05506 | 0.961 | ns |
| SCF055 | SCF05804 | 0.98 | ns |
| SCF055 | SCF082 | 0.747 | ns |
| SCF055 | CCMP3420 | 0.0194 | * |
| SCF055 | CCMP2548 | 0.225 | ns |
| SCF055 | CCMP2464 | 0.00102 | ** |
| SCF05506 | SCF05804 | 0.497 | ns |
| SCF05506 | SCF082 | 1 | ns |
| SCF05506 | CCMP3420 | 0.383 | ns |
| SCF05506 | CCMP2548 | 0.956 | ns |
| SCF05506 | CCMP2464 | 0.0433 | * |
| SCF05804 | SCF082 | 0.186 | ns |
| SCF05804 | CCMP3420 | 0.00194 | ** |
| SCF05804 | CCMP2548 | 0.0291 | * |
| SCF05804 | CCMP2464 | 1.08E-04 | *** |
| SCF082 | CCMP3420 | 0.455 | ns |
| SCF082 | CCMP2548 | 0.99 | ns |
| SCF082 | CCMP2464 | 0.0419 | * |
| CCMP3420 | CCMP2548 | 0.946 | ns |
| CCMP3420 | CCMP2464 | 0.915 | ns |
| CCMP2548 | CCMP2464 | 0.25 | ns |

1. Tukey post hoc test PC2

| group1 | group2 | *p*.adj | *p*.adj.signif |
| --- | --- | --- | --- |
| B(UTS) | PVB18B | 1 | ns |
| B(UTS) | CCMP2463 | 1 | ns |
| B(UTS) | SCF055 | 0.98 | ns |
| B(UTS) | SCF05506 | 0.999 | ns |
| B(UTS) | SCF05804 | 0.65 | ns |
| B(UTS) | SCF082 | 1 | ns |
| B(UTS) | CCMP3420 | 0.999 | ns |
| B(UTS) | CCMP2548 | 0.999 | ns |
| B(UTS) | CCMP2464 | 0.016 | * |
| PVB18B | CCMP2463 | 0.998 | ns |
| PVB18B | SCF055 | 0.95 | ns |
| PVB18B | SCF05506 | 0.995 | ns |
| PVB18B | SCF05804 | 0.544 | ns |
| PVB18B | SCF082 | 1 | ns |
| PVB18B | CCMP3420 | 0.997 | ns |
| PVB18B | CCMP2548 | 1 | ns |
| PVB18B | CCMP2464 | 0.0111 | * |
| CCMP2463 | SCF055 | 1 | ns |
| CCMP2463 | SCF05506 | 1 | ns |
| CCMP2463 | SCF05804 | 0.941 | ns |
| CCMP2463 | SCF082 | 1 | ns |
| CCMP2463 | CCMP3420 | 1 | ns |
| CCMP2463 | CCMP2548 | 0.931 | ns |
| CCMP2463 | CCMP2464 | 0.0575 | ns |
| SCF055 | SCF05506 | 1 | ns |
| SCF055 | SCF05804 | 0.997 | ns |
| SCF055 | SCF082 | 0.994 | ns |
| SCF055 | CCMP3420 | 1 | ns |
| SCF055 | CCMP2548 | 0.727 | ns |
| SCF055 | CCMP2464 | 0.137 | ns |
| SCF05506 | SCF05804 | 0.989 | ns |
| SCF05506 | SCF082 | 1 | ns |
| SCF05506 | CCMP3420 | 1 | ns |
| SCF05506 | CCMP2548 | 0.923 | ns |
| SCF05506 | CCMP2464 | 0.157 | ns |
| SCF05804 | SCF082 | 0.751 | ns |
| SCF05804 | CCMP3420 | 0.949 | ns |
| SCF05804 | CCMP2548 | 0.263 | ns |
| SCF05804 | CCMP2464 | 0.496 | ns |
| SCF082 | CCMP3420 | 1 | ns |
| SCF082 | CCMP2548 | 0.995 | ns |
| SCF082 | CCMP2464 | 0.0229 | * |
| CCMP3420 | CCMP2548 | 0.921 | ns |
| CCMP3420 | CCMP2464 | 0.0613 | ns |
| CCMP2548 | CCMP2464 | 0.00353 | ** |

**SI Table 3 Loadings of E:P in the first (PC1) and second (PC2) principal Components of the Principal Component Analysis (PCA) of the 10 Symbiodiniaceae isolates at 27.4°C.**

| Elements | PC1 | PC2 |
| --- | --- | --- |
| S | 1.024039 | 0.254777 |
| Ni | 0.866939 | 0.575654 |
| Sr | 0.829235 | 0.544862 |
| Fe | 0.805062 | 0.601519 |
| S | 0.794935 | 0.489301 |
| N | 0.720931 | 0.105985 |
| Cu | 0.690705 | 0.010227 |
| Ca | 0.484456 | 0.852685 |
| C | 0.470875 | 0.155156 |
| Mn | 0.190947 | 0.759222 |
| K | 0.18119 | 0.361512 |
| Zn | 0.12036 | 0.727056 |
| Mo | 0.094166 | 0.313109 |
| V | 0.087333 | 0.864925 |

**SI Table 4 Assessment of differences in E:P ratios for the ten Symbiodiniaceae isolates.** Differences were assessed via Analysis of Variance (ANOVA) with post hoc Tukey test. Levene’s test was applied to assess for equal variance, and Shapiro-Wilk for normality combined with manual inspections of QQ-plots of the model residuals. DFn is the degrees of freedom numerator, Dfd is the degrees of freedom denominator and ges is the generalised eta squared that is a useful metric for evaluating the size of an effect (>.26 considered large; Bakeman, 2005). Ns *p* > 0.05, * *p* ≤ 0.05 ** *p* ≤ 0.01 *** *p* ≤ 0.001.

1. ANOVA

| Elements | DFn | DFd | *F* | *p* | *P <.05* | ges |
| --- | --- | --- | --- | --- | --- | --- |
| C:P | 9 | 19 | 2.32 | 0.059 | ns | 0.524 |
| Ca:P | 9 | 19 | 8.771 | 4.13E-05 | * | 0.806 |
| Cu:P | 9 | 19 | 2.47 | 0.047 | * | 0.539 |
| Fe:P | 9 | 19 | 4.547 | 0.003 | * | 0.683 |
| K:P | 9 | 19 | 1.658 | 0.169 | ns | 0.44 |
| Mn:P | 9 | 19 | 2.109 | 0.082 | ns | 0.5 |
| Mo:P | 9 | 19 | 1.02 | 0.459 | ns | 0.326 |
| N:P | 9 | 19 | 2.449 | 0.048 | * | 0.537 |
| Ni:P | 9 | 19 | 4.98 | 0.002 | * | 0.702 |
| S:P | 9 | 19 | 26.696 | 6.13E-09 | * | 0.927 |
| Se:P | 9 | 19 | 4.876 | 0.002 | * | 0.698 |
| Sr:P | 9 | 19 | 6.537 | 3.01E-04 | * | 0.756 |
| V:P | 9 | 19 | 4.016 | 0.005 | * | 0.655 |
| Zn:P | 9 | 19 | 1.426 | 0.245 | ns | 0.403 |

1. Tukey post hoc test

| Elements | group1 | group2 | *p*.adj | *p*.adj.signif |
| --- | --- | --- | --- | --- |
| C | CCMP2548 | CCMP3420 | 1 | ns |
| C | CCMP2548 | B(UTS) | 0.992 | ns |
| C | CCMP2548 | SCF055 | 1 | ns |
| C | CCMP2548 | PVB18B | 1 | ns |
| C | CCMP2548 | CCMP2463 | 0.448 | ns |
| C | CCMP2548 | CCMP2464 | 0.932 | ns |
| C | CCMP2548 | SCF05506 | 0.159 | ns |
| C | CCMP2548 | SCF05804 | 0.822 | ns |
| C | CCMP2548 | SCF082 | 0.995 | ns |
| C | CCMP3420 | B(UTS) | 1 | ns |
| C | CCMP3420 | SCF055 | 0.97 | ns |
| C | CCMP3420 | PVB18B | 0.999 | ns |
| C | CCMP3420 | CCMP2463 | 0.803 | ns |
| C | CCMP3420 | CCMP2464 | 0.999 | ns |
| C | CCMP3420 | SCF05506 | 0.377 | ns |
| C | CCMP3420 | SCF05804 | 0.988 | ns |
| C | CCMP3420 | SCF082 | 1 | ns |
| C | B(UTS) | SCF055 | 0.876 | ns |
| C | B(UTS) | PVB18B | 0.982 | ns |
| C | B(UTS) | CCMP2463 | 0.936 | ns |
| C | B(UTS) | CCMP2464 | 1 | ns |
| C | B(UTS) | SCF05506 | 0.549 | ns |
| C | B(UTS) | SCF05804 | 0.999 | ns |
| C | B(UTS) | SCF082 | 1 | ns |
| C | SCF055 | PVB18B | 1 | ns |
| C | SCF055 | CCMP2463 | 0.196 | ns |
| C | SCF055 | CCMP2464 | 0.675 | ns |
| C | SCF055 | SCF05506 | 0.0635 | ns |
| C | SCF055 | SCF05804 | 0.502 | ns |
| C | SCF055 | SCF082 | 0.897 | ns |
| C | PVB18B | CCMP2463 | 0.381 | ns |
| C | PVB18B | CCMP2464 | 0.892 | ns |
| C | PVB18B | SCF05506 | 0.131 | ns |
| C | PVB18B | SCF05804 | 0.759 | ns |
| C | PVB18B | SCF082 | 0.988 | ns |
| C | CCMP2463 | CCMP2464 | 0.993 | ns |
| C | CCMP2463 | SCF05506 | 0.994 | ns |
| C | CCMP2463 | SCF05804 | 1 | ns |
| C | CCMP2463 | SCF082 | 0.919 | ns |
| C | CCMP2464 | SCF05506 | 0.756 | ns |
| C | CCMP2464 | SCF05804 | 1 | ns |
| C | CCMP2464 | SCF082 | 1 | ns |
| C | SCF05506 | SCF05804 | 0.879 | ns |
| C | SCF05506 | SCF082 | 0.519 | ns |
| C | SCF05804 | SCF082 | 0.999 | ns |
| Ca | CCMP2548 | CCMP3420 | 0.137 | ns |
| Ca | CCMP2548 | B(UTS) | 1 | ns |
| Ca | CCMP2548 | SCF055 | 0.958 | ns |
| Ca | CCMP2548 | PVB18B | 0.988 | ns |
| Ca | CCMP2548 | CCMP2463 | 1 | ns |
| Ca | CCMP2548 | CCMP2464 | 0.00116 | ** |
| Ca | CCMP2548 | SCF05506 | 0.989 | ns |
| Ca | CCMP2548 | SCF05804 | 0.976 | ns |
| Ca | CCMP2548 | SCF082 | 1 | ns |
| Ca | CCMP3420 | B(UTS) | 0.103 | ns |
| Ca | CCMP3420 | SCF055 | 0.0118 | * |
| Ca | CCMP3420 | PVB18B | 0.0186 | * |
| Ca | CCMP3420 | CCMP2463 | 0.0674 | ns |
| Ca | CCMP3420 | CCMP2464 | 0.419 | ns |
| Ca | CCMP3420 | SCF05506 | 0.0376 | * |
| Ca | CCMP3420 | SCF05804 | 0.66 | ns |
| Ca | CCMP3420 | SCF082 | 0.0723 | ns |
| Ca | B(UTS) | SCF055 | 0.981 | ns |
| Ca | B(UTS) | PVB18B | 0.996 | ns |
| Ca | B(UTS) | CCMP2463 | 1 | ns |
| Ca | B(UTS) | CCMP2464 | 8.42E-04 | *** |
| Ca | B(UTS) | SCF05506 | 0.996 | ns |
| Ca | B(UTS) | SCF05804 | 0.949 | ns |
| Ca | B(UTS) | SCF082 | 1 | ns |
| Ca | SCF055 | PVB18B | 1 | ns |
| Ca | SCF055 | CCMP2463 | 0.996 | ns |
| Ca | SCF055 | CCMP2464 | 9.21E-05 | **** |
| Ca | SCF055 | SCF05506 | 1 | ns |
| Ca | SCF055 | SCF05804 | 0.405 | ns |
| Ca | SCF055 | SCF082 | 0.995 | ns |
| Ca | PVB18B | CCMP2463 | 1 | ns |
| Ca | PVB18B | CCMP2464 | 1.42E-04 | *** |
| Ca | PVB18B | SCF05506 | 1 | ns |
| Ca | PVB18B | SCF05804 | 0.529 | ns |
| Ca | PVB18B | SCF082 | 0.999 | ns |
| Ca | CCMP2463 | CCMP2464 | 5.27E-04 | *** |
| Ca | CCMP2463 | SCF05506 | 1 | ns |
| Ca | CCMP2463 | SCF05804 | 0.878 | ns |
| Ca | CCMP2463 | SCF082 | 1 | ns |
| Ca | CCMP2464 | SCF05506 | 4.76E-04 | *** |
| Ca | CCMP2464 | SCF05804 | 0.0125 | * |
| Ca | CCMP2464 | SCF082 | 5.69E-04 | *** |
| Ca | SCF05506 | SCF05804 | 0.614 | ns |
| Ca | SCF05506 | SCF082 | 0.999 | ns |
| Ca | SCF05804 | SCF082 | 0.892 | ns |
| Cu | CCMP2548 | CCMP3420 | 1 | ns |
| Cu | CCMP2548 | B(UTS) | 1 | ns |
| Cu | CCMP2548 | SCF055 | 1 | ns |
| Cu | CCMP2548 | PVB18B | 1 | ns |
| Cu | CCMP2548 | CCMP2463 | 0.943 | ns |
| Cu | CCMP2548 | CCMP2464 | 1 | ns |
| Cu | CCMP2548 | SCF05506 | 1 | ns |
| Cu | CCMP2548 | SCF05804 | 0.175 | ns |
| Cu | CCMP2548 | SCF082 | 1 | ns |
| Cu | CCMP3420 | B(UTS) | 1 | ns |
| Cu | CCMP3420 | SCF055 | 1 | ns |
| Cu | CCMP3420 | PVB18B | 1 | ns |
| Cu | CCMP3420 | CCMP2463 | 0.951 | ns |
| Cu | CCMP3420 | CCMP2464 | 1 | ns |
| Cu | CCMP3420 | SCF05506 | 1 | ns |
| Cu | CCMP3420 | SCF05804 | 0.166 | ns |
| Cu | CCMP3420 | SCF082 | 0.999 | ns |
| Cu | B(UTS) | SCF055 | 1 | ns |
| Cu | B(UTS) | PVB18B | 1 | ns |
| Cu | B(UTS) | CCMP2463 | 0.988 | ns |
| Cu | B(UTS) | CCMP2464 | 1 | ns |
| Cu | B(UTS) | SCF05506 | 1 | ns |
| Cu | B(UTS) | SCF05804 | 0.106 | ns |
| Cu | B(UTS) | SCF082 | 0.993 | ns |
| Cu | SCF055 | PVB18B | 0.993 | ns |
| Cu | SCF055 | CCMP2463 | 0.999 | ns |
| Cu | SCF055 | CCMP2464 | 1 | ns |
| Cu | SCF055 | SCF05506 | 1 | ns |
| Cu | SCF055 | SCF05804 | 0.0592 | ns |
| Cu | SCF055 | SCF082 | 0.957 | ns |
| Cu | PVB18B | CCMP2463 | 0.811 | ns |
| Cu | PVB18B | CCMP2464 | 0.997 | ns |
| Cu | PVB18B | SCF05506 | 1 | ns |
| Cu | PVB18B | SCF05804 | 0.309 | ns |
| Cu | PVB18B | SCF082 | 1 | ns |
| Cu | CCMP2463 | CCMP2464 | 0.997 | ns |
| Cu | CCMP2463 | SCF05506 | 0.986 | ns |
| Cu | CCMP2463 | SCF05804 | 0.0139 | * |
| Cu | CCMP2463 | SCF082 | 0.646 | ns |
| Cu | CCMP2464 | SCF05506 | 1 | ns |
| Cu | CCMP2464 | SCF05804 | 0.0723 | ns |
| Cu | CCMP2464 | SCF082 | 0.975 | ns |
| Cu | SCF05506 | SCF05804 | 0.235 | ns |
| Cu | SCF05506 | SCF082 | 0.999 | ns |
| Cu | SCF05804 | SCF082 | 0.462 | ns |
| Fe | CCMP2548 | CCMP3420 | 0.997 | ns |
| Fe | CCMP2548 | B(UTS) | 0.553 | ns |
| Fe | CCMP2548 | SCF055 | 0.987 | ns |
| Fe | CCMP2548 | PVB18B | 0.997 | ns |
| Fe | CCMP2548 | CCMP2463 | 0.456 | ns |
| Fe | CCMP2548 | CCMP2464 | 1 | ns |
| Fe | CCMP2548 | SCF05506 | 1 | ns |
| Fe | CCMP2548 | SCF05804 | 0.184 | ns |
| Fe | CCMP2548 | SCF082 | 1 | ns |
| Fe | CCMP3420 | B(UTS) | 0.949 | ns |
| Fe | CCMP3420 | SCF055 | 0.706 | ns |
| Fe | CCMP3420 | PVB18B | 0.803 | ns |
| Fe | CCMP3420 | CCMP2463 | 0.9 | ns |
| Fe | CCMP3420 | CCMP2464 | 0.916 | ns |
| Fe | CCMP3420 | SCF05506 | 0.994 | ns |
| Fe | CCMP3420 | SCF05804 | 0.0392 | * |
| Fe | CCMP3420 | SCF082 | 1 | ns |
| Fe | B(UTS) | SCF055 | 0.119 | ns |
| Fe | B(UTS) | PVB18B | 0.163 | ns |
| Fe | B(UTS) | CCMP2463 | 1 | ns |
| Fe | B(UTS) | CCMP2464 | 0.256 | ns |
| Fe | B(UTS) | SCF05506 | 0.581 | ns |
| Fe | B(UTS) | SCF05804 | 0.00272 | ** |
| Fe | B(UTS) | SCF082 | 0.659 | ns |
| Fe | SCF055 | PVB18B | 1 | ns |
| Fe | SCF055 | CCMP2463 | 0.0875 | ns |
| Fe | SCF055 | CCMP2464 | 1 | ns |
| Fe | SCF055 | SCF05506 | 0.999 | ns |
| Fe | SCF055 | SCF05804 | 0.701 | ns |
| Fe | SCF055 | SCF082 | 0.965 | ns |
| Fe | PVB18B | CCMP2463 | 0.122 | ns |
| Fe | PVB18B | CCMP2464 | 1 | ns |
| Fe | PVB18B | SCF05506 | 1 | ns |
| Fe | PVB18B | SCF05804 | 0.595 | ns |
| Fe | PVB18B | SCF082 | 0.987 | ns |
| Fe | CCMP2463 | CCMP2464 | 0.196 | ns |
| Fe | CCMP2463 | SCF05506 | 0.493 | ns |
| Fe | CCMP2463 | SCF05804 | 0.00192 | ** |
| Fe | CCMP2463 | SCF082 | 0.559 | ns |
| Fe | CCMP2464 | SCF05506 | 1 | ns |
| Fe | CCMP2464 | SCF05804 | 0.434 | ns |
| Fe | CCMP2464 | SCF082 | 0.999 | ns |
| Fe | SCF05506 | SCF05804 | 0.384 | ns |
| Fe | SCF05506 | SCF082 | 1 | ns |
| Fe | SCF05804 | SCF082 | 0.135 | ns |
| K | CCMP2548 | CCMP3420 | 1 | ns |
| K | CCMP2548 | B(UTS) | 0.457 | ns |
| K | CCMP2548 | SCF055 | 0.968 | ns |
| K | CCMP2548 | PVB18B | 1 | ns |
| K | CCMP2548 | CCMP2463 | 0.803 | ns |
| K | CCMP2548 | CCMP2464 | 0.962 | ns |
| K | CCMP2548 | SCF05506 | 1 | ns |
| K | CCMP2548 | SCF05804 | 0.997 | ns |
| K | CCMP2548 | SCF082 | 1 | ns |
| K | CCMP3420 | B(UTS) | 0.359 | ns |
| K | CCMP3420 | SCF055 | 0.928 | ns |
| K | CCMP3420 | PVB18B | 1 | ns |
| K | CCMP3420 | CCMP2463 | 0.704 | ns |
| K | CCMP3420 | CCMP2464 | 0.916 | ns |
| K | CCMP3420 | SCF05506 | 1 | ns |
| K | CCMP3420 | SCF05804 | 0.987 | ns |
| K | CCMP3420 | SCF082 | 1 | ns |
| K | B(UTS) | SCF055 | 0.981 | ns |
| K | B(UTS) | PVB18B | 0.412 | ns |
| K | B(UTS) | CCMP2463 | 1 | ns |
| K | B(UTS) | CCMP2464 | 0.985 | ns |
| K | B(UTS) | SCF05506 | 0.705 | ns |
| K | B(UTS) | SCF05804 | 0.907 | ns |
| K | B(UTS) | SCF082 | 0.199 | ns |
| K | SCF055 | PVB18B | 0.953 | ns |
| K | SCF055 | CCMP2463 | 1 | ns |
| K | SCF055 | CCMP2464 | 1 | ns |
| K | SCF055 | SCF05506 | 0.996 | ns |
| K | SCF055 | SCF05804 | 1 | ns |
| K | SCF055 | SCF082 | 0.766 | ns |
| K | PVB18B | CCMP2463 | 0.761 | ns |
| K | PVB18B | CCMP2464 | 0.945 | ns |
| K | PVB18B | SCF05506 | 1 | ns |
| K | PVB18B | SCF05804 | 0.994 | ns |
| K | PVB18B | SCF082 | 1 | ns |
| K | CCMP2463 | CCMP2464 | 1 | ns |
| K | CCMP2463 | SCF05506 | 0.94 | ns |
| K | CCMP2463 | SCF05804 | 0.997 | ns |
| K | CCMP2463 | SCF082 | 0.476 | ns |
| K | CCMP2464 | SCF05506 | 0.994 | ns |
| K | CCMP2464 | SCF05804 | 1 | ns |
| K | CCMP2464 | SCF082 | 0.745 | ns |
| K | SCF05506 | SCF05804 | 1 | ns |
| K | SCF05506 | SCF082 | 0.999 | ns |
| K | SCF05804 | SCF082 | 0.913 | ns |
| Mn | CCMP2548 | CCMP3420 | 1 | ns |
| Mn | CCMP2548 | B(UTS) | 0.951 | ns |
| Mn | CCMP2548 | SCF055 | 1 | ns |
| Mn | CCMP2548 | PVB18B | 1 | ns |
| Mn | CCMP2548 | CCMP2463 | 0.894 | ns |
| Mn | CCMP2548 | CCMP2464 | 0.237 | ns |
| Mn | CCMP2548 | SCF05506 | 1 | ns |
| Mn | CCMP2548 | SCF05804 | 0.999 | ns |
| Mn | CCMP2548 | SCF082 | 0.954 | ns |
| Mn | CCMP3420 | B(UTS) | 0.738 | ns |
| Mn | CCMP3420 | SCF055 | 1 | ns |
| Mn | CCMP3420 | PVB18B | 1 | ns |
| Mn | CCMP3420 | CCMP2463 | 0.993 | ns |
| Mn | CCMP3420 | CCMP2464 | 0.494 | ns |
| Mn | CCMP3420 | SCF05506 | 1 | ns |
| Mn | CCMP3420 | SCF05804 | 1 | ns |
| Mn | CCMP3420 | SCF082 | 0.999 | ns |
| Mn | B(UTS) | SCF055 | 0.787 | ns |
| Mn | B(UTS) | PVB18B | 0.834 | ns |
| Mn | B(UTS) | CCMP2463 | 0.235 | ns |
| Mn | B(UTS) | CCMP2464 | 0.0217 | * |
| Mn | B(UTS) | SCF05506 | 0.807 | ns |
| Mn | B(UTS) | SCF05804 | 0.6 | ns |
| Mn | B(UTS) | SCF082 | 0.323 | ns |
| Mn | SCF055 | PVB18B | 1 | ns |
| Mn | SCF055 | CCMP2463 | 0.987 | ns |
| Mn | SCF055 | CCMP2464 | 0.443 | ns |
| Mn | SCF055 | SCF05506 | 1 | ns |
| Mn | SCF055 | SCF05804 | 1 | ns |
| Mn | SCF055 | SCF082 | 0.997 | ns |
| Mn | PVB18B | CCMP2463 | 0.976 | ns |
| Mn | PVB18B | CCMP2464 | 0.391 | ns |
| Mn | PVB18B | SCF05506 | 1 | ns |
| Mn | PVB18B | SCF05804 | 1 | ns |
| Mn | PVB18B | SCF082 | 0.994 | ns |
| Mn | CCMP2463 | CCMP2464 | 0.952 | ns |
| Mn | CCMP2463 | SCF05506 | 0.998 | ns |
| Mn | CCMP2463 | SCF05804 | 0.999 | ns |
| Mn | CCMP2463 | SCF082 | 1 | ns |
| Mn | CCMP2464 | SCF05506 | 0.669 | ns |
| Mn | CCMP2464 | SCF05804 | 0.635 | ns |
| Mn | CCMP2464 | SCF082 | 0.891 | ns |
| Mn | SCF05506 | SCF05804 | 1 | ns |
| Mn | SCF05506 | SCF082 | 1 | ns |
| Mn | SCF05804 | SCF082 | 1 | ns |
| Mo | CCMP2548 | CCMP3420 | 1 | ns |
| Mo | CCMP2548 | B(UTS) | 1 | ns |
| Mo | CCMP2548 | SCF055 | 1 | ns |
| Mo | CCMP2548 | PVB18B | 1 | ns |
| Mo | CCMP2548 | CCMP2463 | 1 | ns |
| Mo | CCMP2548 | CCMP2464 | 1 | ns |
| Mo | CCMP2548 | SCF05506 | 1 | ns |
| Mo | CCMP2548 | SCF05804 | 0.505 | ns |
| Mo | CCMP2548 | SCF082 | 1 | ns |
| Mo | CCMP3420 | B(UTS) | 1 | ns |
| Mo | CCMP3420 | SCF055 | 1 | ns |
| Mo | CCMP3420 | PVB18B | 1 | ns |
| Mo | CCMP3420 | CCMP2463 | 1 | ns |
| Mo | CCMP3420 | CCMP2464 | 1 | ns |
| Mo | CCMP3420 | SCF05506 | 1 | ns |
| Mo | CCMP3420 | SCF05804 | 0.625 | ns |
| Mo | CCMP3420 | SCF082 | 1 | ns |
| Mo | B(UTS) | SCF055 | 1 | ns |
| Mo | B(UTS) | PVB18B | 1 | ns |
| Mo | B(UTS) | CCMP2463 | 1 | ns |
| Mo | B(UTS) | CCMP2464 | 1 | ns |
| Mo | B(UTS) | SCF05506 | 1 | ns |
| Mo | B(UTS) | SCF05804 | 0.439 | ns |
| Mo | B(UTS) | SCF082 | 1 | ns |
| Mo | SCF055 | PVB18B | 1 | ns |
| Mo | SCF055 | CCMP2463 | 1 | ns |
| Mo | SCF055 | CCMP2464 | 1 | ns |
| Mo | SCF055 | SCF05506 | 1 | ns |
| Mo | SCF055 | SCF05804 | 0.394 | ns |
| Mo | SCF055 | SCF082 | 1 | ns |
| Mo | PVB18B | CCMP2463 | 1 | ns |
| Mo | PVB18B | CCMP2464 | 1 | ns |
| Mo | PVB18B | SCF05506 | 1 | ns |
| Mo | PVB18B | SCF05804 | 0.653 | ns |
| Mo | PVB18B | SCF082 | 1 | ns |
| Mo | CCMP2463 | CCMP2464 | 0.998 | ns |
| Mo | CCMP2463 | SCF05506 | 1 | ns |
| Mo | CCMP2463 | SCF05804 | 0.755 | ns |
| Mo | CCMP2463 | SCF082 | 1 | ns |
| Mo | CCMP2464 | SCF05506 | 1 | ns |
| Mo | CCMP2464 | SCF05804 | 0.321 | ns |
| Mo | CCMP2464 | SCF082 | 0.999 | ns |
| Mo | SCF05506 | SCF05804 | 0.551 | ns |
| Mo | SCF05506 | SCF082 | 1 | ns |
| Mo | SCF05804 | SCF082 | 0.717 | ns |
| N | CCMP2548 | CCMP3420 | 1 | ns |
| N | CCMP2548 | B(UTS) | 0.997 | ns |
| N | CCMP2548 | SCF055 | 0.874 | ns |
| N | CCMP2548 | PVB18B | 1 | ns |
| N | CCMP2548 | CCMP2463 | 0.541 | ns |
| N | CCMP2548 | CCMP2464 | 0.654 | ns |
| N | CCMP2548 | SCF05506 | 0.945 | ns |
| N | CCMP2548 | SCF05804 | 0.977 | ns |
| N | CCMP2548 | SCF082 | 0.965 | ns |
| N | CCMP3420 | B(UTS) | 1 | ns |
| N | CCMP3420 | SCF055 | 0.712 | ns |
| N | CCMP3420 | PVB18B | 0.999 | ns |
| N | CCMP3420 | CCMP2463 | 0.734 | ns |
| N | CCMP3420 | CCMP2464 | 0.831 | ns |
| N | CCMP3420 | SCF05506 | 0.989 | ns |
| N | CCMP3420 | SCF05804 | 0.998 | ns |
| N | CCMP3420 | SCF082 | 0.996 | ns |
| N | B(UTS) | SCF055 | 0.418 | ns |
| N | B(UTS) | PVB18B | 0.956 | ns |
| N | B(UTS) | CCMP2463 | 0.943 | ns |
| N | B(UTS) | CCMP2464 | 0.978 | ns |
| N | B(UTS) | SCF05506 | 1 | ns |
| N | B(UTS) | SCF05804 | 1 | ns |
| N | B(UTS) | SCF082 | 1 | ns |
| N | SCF055 | PVB18B | 0.98 | ns |
| N | SCF055 | CCMP2463 | 0.0449 | * |
| N | SCF055 | CCMP2464 | 0.0647 | ns |
| N | SCF055 | SCF05506 | 0.267 | ns |
| N | SCF055 | SCF05804 | 0.275 | ns |
| N | SCF055 | SCF082 | 0.243 | ns |
| N | PVB18B | CCMP2463 | 0.314 | ns |
| N | PVB18B | CCMP2464 | 0.407 | ns |
| N | PVB18B | SCF05506 | 0.802 | ns |
| N | PVB18B | SCF05804 | 0.865 | ns |
| N | PVB18B | SCF082 | 0.829 | ns |
| N | CCMP2463 | CCMP2464 | 1 | ns |
| N | CCMP2463 | SCF05506 | 1 | ns |
| N | CCMP2463 | SCF05804 | 0.989 | ns |
| N | CCMP2463 | SCF082 | 0.994 | ns |
| N | CCMP2464 | SCF05506 | 1 | ns |
| N | CCMP2464 | SCF05804 | 0.997 | ns |
| N | CCMP2464 | SCF082 | 0.999 | ns |
| N | SCF05506 | SCF05804 | 1 | ns |
| N | SCF05506 | SCF082 | 1 | ns |
| N | SCF05804 | SCF082 | 1 | ns |
| Ni | CCMP2548 | CCMP3420 | 1 | ns |
| Ni | CCMP2548 | B(UTS) | 1 | ns |
| Ni | CCMP2548 | SCF055 | 0.0832 | ns |
| Ni | CCMP2548 | PVB18B | 0.86 | ns |
| Ni | CCMP2548 | CCMP2463 | 1 | ns |
| Ni | CCMP2548 | CCMP2464 | 0.807 | ns |
| Ni | CCMP2548 | SCF05506 | 0.131 | ns |
| Ni | CCMP2548 | SCF05804 | 0.0159 | * |
| Ni | CCMP2548 | SCF082 | 0.986 | ns |
| Ni | CCMP3420 | B(UTS) | 0.999 | ns |
| Ni | CCMP3420 | SCF055 | 0.228 | ns |
| Ni | CCMP3420 | PVB18B | 0.991 | ns |
| Ni | CCMP3420 | CCMP2463 | 0.989 | ns |
| Ni | CCMP3420 | CCMP2464 | 0.98 | ns |
| Ni | CCMP3420 | SCF05506 | 0.305 | ns |
| Ni | CCMP3420 | SCF05804 | 0.0508 | ns |
| Ni | CCMP3420 | SCF082 | 1 | ns |
| Ni | B(UTS) | SCF055 | 0.0589 | ns |
| Ni | B(UTS) | PVB18B | 0.773 | ns |
| Ni | B(UTS) | CCMP2463 | 1 | ns |
| Ni | B(UTS) | CCMP2464 | 0.71 | ns |
| Ni | B(UTS) | SCF05506 | 0.0978 | ns |
| Ni | B(UTS) | SCF05804 | 0.0109 | * |
| Ni | B(UTS) | SCF082 | 0.961 | ns |
| Ni | SCF055 | PVB18B | 0.75 | ns |
| Ni | SCF055 | CCMP2463 | 0.0359 | * |
| Ni | SCF055 | CCMP2464 | 0.809 | ns |
| Ni | SCF055 | SCF05506 | 1 | ns |
| Ni | SCF055 | SCF05804 | 0.997 | ns |
| Ni | SCF055 | SCF082 | 0.451 | ns |
| Ni | PVB18B | CCMP2463 | 0.63 | ns |
| Ni | PVB18B | CCMP2464 | 1 | ns |
| Ni | PVB18B | SCF05506 | 0.794 | ns |
| Ni | PVB18B | SCF05804 | 0.29 | ns |
| Ni | PVB18B | SCF082 | 1 | ns |
| Ni | CCMP2463 | CCMP2464 | 0.561 | ns |
| Ni | CCMP2463 | SCF05506 | 0.0641 | ns |
| Ni | CCMP2463 | SCF05804 | 0.00648 | ** |
| Ni | CCMP2463 | SCF082 | 0.892 | ns |
| Ni | CCMP2464 | SCF05506 | 0.843 | ns |
| Ni | CCMP2464 | SCF05804 | 0.342 | ns |
| Ni | CCMP2464 | SCF082 | 1 | ns |
| Ni | SCF05506 | SCF05804 | 1 | ns |
| Ni | SCF05506 | SCF082 | 0.53 | ns |
| Ni | SCF05804 | SCF082 | 0.124 | ns |
| S | CCMP2548 | CCMP3420 | 0.879 | ns |
| S | CCMP2548 | B(UTS) | 1 | ns |
| S | CCMP2548 | SCF055 | 0.00248 | ** |
| S | CCMP2548 | PVB18B | 0.00119 | ** |
| S | CCMP2548 | CCMP2463 | 0.0305 | * |
| S | CCMP2548 | CCMP2464 | 0.00805 | ** |
| S | CCMP2548 | SCF05506 | 0.193 | ns |
| S | CCMP2548 | SCF05804 | 0.0335 | * |
| S | CCMP2548 | SCF082 | 0.00454 | ** |
| S | CCMP3420 | B(UTS) | 0.874 | ns |
| S | CCMP3420 | SCF055 | 1.11E-04 | *** |
| S | CCMP3420 | PVB18B | 5.57E-05 | **** |
| S | CCMP3420 | CCMP2463 | 0.424 | ns |
| S | CCMP3420 | CCMP2464 | 0.158 | ns |
| S | CCMP3420 | SCF05506 | 0.0144 | * |
| S | CCMP3420 | SCF05804 | 0.00144 | ** |
| S | CCMP3420 | SCF082 | 1.98E-04 | *** |
| S | B(UTS) | SCF055 | 0.00255 | ** |
| S | B(UTS) | PVB18B | 0.00122 | ** |
| S | B(UTS) | CCMP2463 | 0.0297 | * |
| S | B(UTS) | CCMP2464 | 0.00785 | ** |
| S | B(UTS) | SCF05506 | 0.197 | ns |
| S | B(UTS) | SCF05804 | 0.0343 | * |
| S | B(UTS) | SCF082 | 0.00465 | ** |
| S | SCF055 | PVB18B | 1 | ns |
| S | SCF055 | CCMP2463 | 1.46E-06 | **** |
| S | SCF055 | CCMP2464 | 4.94E-07 | **** |
| S | SCF055 | SCF05506 | 0.816 | ns |
| S | SCF055 | SCF05804 | 0.956 | ns |
| S | SCF055 | SCF082 | 1 | ns |
| S | PVB18B | CCMP2463 | 8.02E-07 | **** |
| S | PVB18B | CCMP2464 | 2.79E-07 | **** |
| S | PVB18B | SCF05506 | 0.641 | ns |
| S | PVB18B | SCF05804 | 0.84 | ns |
| S | PVB18B | SCF082 | 1 | ns |
| S | CCMP2463 | CCMP2464 | 1 | ns |
| S | CCMP2463 | SCF05506 | 1.85E-04 | *** |
| S | CCMP2463 | SCF05804 | 1.36E-05 | **** |
| S | CCMP2463 | SCF082 | 2.39E-06 | **** |
| S | CCMP2464 | SCF05506 | 5.90E-05 | **** |
| S | CCMP2464 | SCF05804 | 4.21E-06 | **** |
| S | CCMP2464 | SCF082 | 7.94E-07 | **** |
| S | SCF05506 | SCF05804 | 1 | ns |
| S | SCF05506 | SCF082 | 0.918 | ns |
| S | SCF05804 | SCF082 | 0.992 | ns |
| Se | CCMP2548 | CCMP3420 | 1 | ns |
| Se | CCMP2548 | B(UTS) | 0.904 | ns |
| Se | CCMP2548 | SCF055 | 0.328 | ns |
| Se | CCMP2548 | PVB18B | 0.974 | ns |
| Se | CCMP2548 | CCMP2463 | 0.872 | ns |
| Se | CCMP2548 | CCMP2464 | 0.967 | ns |
| Se | CCMP2548 | SCF05506 | 0.101 | ns |
| Se | CCMP2548 | SCF05804 | 8.90E-04 | *** |
| Se | CCMP2548 | SCF082 | 0.832 | ns |
| Se | CCMP3420 | B(UTS) | 0.997 | ns |
| Se | CCMP3420 | SCF055 | 0.659 | ns |
| Se | CCMP3420 | PVB18B | 1 | ns |
| Se | CCMP3420 | CCMP2463 | 0.993 | ns |
| Se | CCMP3420 | CCMP2464 | 1 | ns |
| Se | CCMP3420 | SCF05506 | 0.25 | ns |
| Se | CCMP3420 | SCF05804 | 0.00306 | ** |
| Se | CCMP3420 | SCF082 | 0.987 | ns |
| Se | B(UTS) | SCF055 | 0.982 | ns |
| Se | B(UTS) | PVB18B | 1 | ns |
| Se | B(UTS) | CCMP2463 | 1 | ns |
| Se | B(UTS) | CCMP2464 | 1 | ns |
| Se | B(UTS) | SCF05506 | 0.656 | ns |
| Se | B(UTS) | SCF05804 | 0.0182 | * |
| Se | B(UTS) | SCF082 | 1 | ns |
| Se | SCF055 | PVB18B | 0.925 | ns |
| Se | SCF055 | CCMP2463 | 0.99 | ns |
| Se | SCF055 | CCMP2464 | 0.937 | ns |
| Se | SCF055 | SCF05506 | 0.991 | ns |
| Se | SCF055 | SCF05804 | 0.149 | ns |
| Se | SCF055 | SCF082 | 0.995 | ns |
| Se | PVB18B | CCMP2463 | 1 | ns |
| Se | PVB18B | CCMP2464 | 1 | ns |
| Se | PVB18B | SCF05506 | 0.501 | ns |
| Se | PVB18B | SCF05804 | 0.00996 | ** |
| Se | PVB18B | SCF082 | 1 | ns |
| Se | CCMP2463 | CCMP2464 | 1 | ns |
| Se | CCMP2463 | SCF05506 | 0.701 | ns |
| Se | CCMP2463 | SCF05804 | 0.0217 | * |
| Se | CCMP2463 | SCF082 | 1 | ns |
| Se | CCMP2464 | SCF05506 | 0.523 | ns |
| Se | CCMP2464 | SCF05804 | 0.0109 | * |
| Se | CCMP2464 | SCF082 | 1 | ns |
| Se | SCF05506 | SCF05804 | 0.779 | ns |
| Se | SCF05506 | SCF082 | 0.747 | ns |
| Se | SCF05804 | SCF082 | 0.0261 | * |
| Sr | CCMP2548 | CCMP3420 | 0.319 | ns |
| Sr | CCMP2548 | B(UTS) | 1 | ns |
| Sr | CCMP2548 | SCF055 | 0.998 | ns |
| Sr | CCMP2548 | PVB18B | 0.999 | ns |
| Sr | CCMP2548 | CCMP2463 | 1 | ns |
| Sr | CCMP2548 | CCMP2464 | 0.0228 | * |
| Sr | CCMP2548 | SCF05506 | 0.981 | ns |
| Sr | CCMP2548 | SCF05804 | 0.425 | ns |
| Sr | CCMP2548 | SCF082 | 1 | ns |
| Sr | CCMP3420 | B(UTS) | 0.229 | ns |
| Sr | CCMP3420 | SCF055 | 0.0858 | ns |
| Sr | CCMP3420 | PVB18B | 0.101 | ns |
| Sr | CCMP3420 | CCMP2463 | 0.277 | ns |
| Sr | CCMP3420 | CCMP2464 | 0.902 | ns |
| Sr | CCMP3420 | SCF05506 | 0.0782 | ns |
| Sr | CCMP3420 | SCF05804 | 0.00356 | ** |
| Sr | CCMP3420 | SCF082 | 0.155 | ns |
| Sr | B(UTS) | SCF055 | 1 | ns |
| Sr | B(UTS) | PVB18B | 1 | ns |
| Sr | B(UTS) | CCMP2463 | 1 | ns |
| Sr | B(UTS) | CCMP2464 | 0.0145 | * |
| Sr | B(UTS) | SCF05506 | 0.995 | ns |
| Sr | B(UTS) | SCF05804 | 0.55 | ns |
| Sr | B(UTS) | SCF082 | 1 | ns |
| Sr | SCF055 | PVB18B | 1 | ns |
| Sr | SCF055 | CCMP2463 | 0.999 | ns |
| Sr | SCF055 | CCMP2464 | 0.00451 | ** |
| Sr | SCF055 | SCF05506 | 1 | ns |
| Sr | SCF055 | SCF05804 | 0.858 | ns |
| Sr | SCF055 | SCF082 | 1 | ns |
| Sr | PVB18B | CCMP2463 | 1 | ns |
| Sr | PVB18B | CCMP2464 | 0.00543 | ** |
| Sr | PVB18B | SCF05506 | 1 | ns |
| Sr | PVB18B | SCF05804 | 0.818 | ns |
| Sr | PVB18B | SCF082 | 1 | ns |
| Sr | CCMP2463 | CCMP2464 | 0.0188 | * |
| Sr | CCMP2463 | SCF05506 | 0.989 | ns |
| Sr | CCMP2463 | SCF05804 | 0.478 | ns |
| Sr | CCMP2463 | SCF082 | 1 | ns |
| Sr | CCMP2464 | SCF05506 | 0.00562 | ** |
| Sr | CCMP2464 | SCF05804 | 1.78E-04 | *** |
| Sr | CCMP2464 | SCF082 | 0.00895 | ** |
| Sr | SCF05506 | SCF05804 | 0.99 | ns |
| Sr | SCF05506 | SCF082 | 0.999 | ns |
| Sr | SCF05804 | SCF082 | 0.69 | ns |
| V | CCMP2548 | CCMP3420 | 0.998 | ns |
| V | CCMP2548 | B(UTS) | 0.183 | ns |
| V | CCMP2548 | SCF055 | 0.484 | ns |
| V | CCMP2548 | PVB18B | 0.895 | ns |
| V | CCMP2548 | CCMP2463 | 0.336 | ns |
| V | CCMP2548 | CCMP2464 | 0.00116 | ** |
| V | CCMP2548 | SCF05506 | 0.435 | ns |
| V | CCMP2548 | SCF05804 | 0.279 | ns |
| V | CCMP2548 | SCF082 | 0.505 | ns |
| V | CCMP3420 | B(UTS) | 0.572 | ns |
| V | CCMP3420 | SCF055 | 0.912 | ns |
| V | CCMP3420 | PVB18B | 1 | ns |
| V | CCMP3420 | CCMP2463 | 0.794 | ns |
| V | CCMP3420 | CCMP2464 | 0.00642 | ** |
| V | CCMP3420 | SCF05506 | 0.845 | ns |
| V | CCMP3420 | SCF05804 | 0.727 | ns |
| V | CCMP3420 | SCF082 | 0.923 | ns |
| V | B(UTS) | SCF055 | 0.999 | ns |
| V | B(UTS) | PVB18B | 0.908 | ns |
| V | B(UTS) | CCMP2463 | 1 | ns |
| V | B(UTS) | CCMP2464 | 0.332 | ns |
| V | B(UTS) | SCF05506 | 1 | ns |
| V | B(UTS) | SCF05804 | 1 | ns |
| V | B(UTS) | SCF082 | 0.999 | ns |
| V | SCF055 | PVB18B | 0.998 | ns |
| V | SCF055 | CCMP2463 | 1 | ns |
| V | SCF055 | CCMP2464 | 0.111 | ns |
| V | SCF055 | SCF05506 | 1 | ns |
| V | SCF055 | SCF05804 | 1 | ns |
| V | SCF055 | SCF082 | 1 | ns |
| V | PVB18B | CCMP2463 | 0.986 | ns |
| V | PVB18B | CCMP2464 | 0.0249 | * |
| V | PVB18B | SCF05506 | 0.989 | ns |
| V | PVB18B | SCF05804 | 0.971 | ns |
| V | PVB18B | SCF082 | 0.999 | ns |
| V | CCMP2463 | CCMP2464 | 0.181 | ns |
| V | CCMP2463 | SCF05506 | 1 | ns |
| V | CCMP2463 | SCF05804 | 1 | ns |
| V | CCMP2463 | SCF082 | 1 | ns |
| V | CCMP2464 | SCF05506 | 0.328 | ns |
| V | CCMP2464 | SCF05804 | 0.222 | ns |
| V | CCMP2464 | SCF082 | 0.104 | ns |
| V | SCF05506 | SCF05804 | 1 | ns |
| V | SCF05506 | SCF082 | 1 | ns |
| V | SCF05804 | SCF082 | 1 | ns |
| Zn | CCMP2548 | CCMP3420 | 0.999 | ns |
| Zn | CCMP2548 | B(UTS) | 0.938 | ns |
| Zn | CCMP2548 | SCF055 | 0.868 | ns |
| Zn | CCMP2548 | PVB18B | 1 | ns |
| Zn | CCMP2548 | CCMP2463 | 0.495 | ns |
| Zn | CCMP2548 | CCMP2464 | 0.317 | ns |
| Zn | CCMP2548 | SCF05506 | 0.944 | ns |
| Zn | CCMP2548 | SCF05804 | 0.628 | ns |
| Zn | CCMP2548 | SCF082 | 0.999 | ns |
| Zn | CCMP3420 | B(UTS) | 1 | ns |
| Zn | CCMP3420 | SCF055 | 0.998 | ns |
| Zn | CCMP3420 | PVB18B | 1 | ns |
| Zn | CCMP3420 | CCMP2463 | 0.887 | ns |
| Zn | CCMP3420 | CCMP2464 | 0.724 | ns |
| Zn | CCMP3420 | SCF05506 | 1 | ns |
| Zn | CCMP3420 | SCF05804 | 0.953 | ns |
| Zn | CCMP3420 | SCF082 | 1 | ns |
| Zn | B(UTS) | SCF055 | 1 | ns |
| Zn | B(UTS) | PVB18B | 0.976 | ns |
| Zn | B(UTS) | CCMP2463 | 0.996 | ns |
| Zn | B(UTS) | CCMP2464 | 0.962 | ns |
| Zn | B(UTS) | SCF05506 | 1 | ns |
| Zn | B(UTS) | SCF05804 | 1 | ns |
| Zn | B(UTS) | SCF082 | 1 | ns |
| Zn | SCF055 | PVB18B | 0.934 | ns |
| Zn | SCF055 | CCMP2463 | 0.999 | ns |
| Zn | SCF055 | CCMP2464 | 0.989 | ns |
| Zn | SCF055 | SCF05506 | 1 | ns |
| Zn | SCF055 | SCF05804 | 1 | ns |
| Zn | SCF055 | SCF082 | 0.997 | ns |
| Zn | PVB18B | CCMP2463 | 0.61 | ns |
| Zn | PVB18B | CCMP2464 | 0.413 | ns |
| Zn | PVB18B | SCF05506 | 0.976 | ns |
| Zn | PVB18B | SCF05804 | 0.74 | ns |
| Zn | PVB18B | SCF082 | 1 | ns |
| Zn | CCMP2463 | CCMP2464 | 1 | ns |
| Zn | CCMP2463 | SCF05506 | 0.999 | ns |
| Zn | CCMP2463 | SCF05804 | 1 | ns |
| Zn | CCMP2463 | SCF082 | 0.872 | ns |
| Zn | CCMP2464 | SCF05506 | 0.991 | ns |
| Zn | CCMP2464 | SCF05804 | 1 | ns |
| Zn | CCMP2464 | SCF082 | 0.702 | ns |
| Zn | SCF05506 | SCF05804 | 1 | ns |
| Zn | SCF05506 | SCF082 | 0.999 | ns |
| Zn | SCF05804 | SCF082 | 0.944 | ns |

**SI Table 5** **Linear regression of mean absolute cellular elemental content with mean cell volume at 27.4°C for all ten Symbiodiniaceae isolates.** *N*= 3 replicates per isolate.

| Elements | r.squared | adj.r.squared | sigma | statistic | p.value | df | logLik | AIC | BIC | deviance | df.residual | nobs |
| --- | --- | --- | --- | --- | --- | --- | --- | --- | --- | --- | --- | --- |
| C | 0.002 | -0.123 | 0.366 | 0.013 | 0.913 | 1 | -3.017 | 12.033 | 12.941 | 1.070 | 8 | 0.002 |
| Ca | 0.028 | -0.094 | 0.361 | 0.229 | 0.645 | 1 | -2.883 | 11.767 | 12.675 | 1.042 | 8 | 0.028 |
| Cu | 0.027 | -0.095 | 0.361 | 0.218 | 0.653 | 1 | -2.890 | 11.781 | 12.688 | 1.044 | 8 | 0.027 |
| Fe | 0.016 | -0.107 | 0.363 | 0.129 | 0.729 | 1 | -2.945 | 11.890 | 12.798 | 1.055 | 8 | 0.016 |
| K | 0.037 | -0.084 | 0.359 | 0.306 | 0.595 | 1 | -2.837 | 11.674 | 12.582 | 1.033 | 8 | 0.037 |
| Mn | 0.155 | 0.049 | 0.336 | 1.469 | 0.260 | 1 | -2.182 | 10.364 | 11.272 | 0.906 | 8 | 0.155 |
| Mo | 0.109 | -0.002 | 0.346 | 0.979 | 0.351 | 1 | -2.447 | 10.895 | 11.802 | 0.955 | 8 | 0.109 |
| N | 0.019 | -0.103 | 0.363 | 0.156 | 0.703 | 1 | -2.928 | 11.856 | 12.764 | 1.052 | 8 | 0.019 |
| Ni | 0.025 | -0.096 | 0.361 | 0.208 | 0.660 | 1 | -2.896 | 11.792 | 12.700 | 1.045 | 8 | 0.025 |
| P | 0.051 | -0.068 | 0.357 | 0.427 | 0.532 | 1 | -2.765 | 11.530 | 12.438 | 1.018 | 8 | 0.051 |
| S | 0.001 | -0.124 | 0.366 | 0.009 | 0.927 | 1 | -3.019 | 12.038 | 12.946 | 1.071 | 8 | 0.001 |
| Se | 0.004 | -0.120 | 0.365 | 0.032 | 0.862 | 1 | -3.004 | 12.009 | 12.917 | 1.068 | 8 | 0.004 |
| Sr | 0.032 | -0.089 | 0.360 | 0.266 | 0.620 | 1 | -2.861 | 11.723 | 12.630 | 1.038 | 8 | 0.032 |
| V | 0.000 | -0.125 | 0.366 | 0.000 | 0.988 | 1 | -3.025 | 12.049 | 12.957 | 1.072 | 8 | 0.000 |
| Zn | 0.001 | -0.124 | 0.366 | 0.005 | 0.944 | 1 | -3.021 | 12.043 | 12.951 | 1.071 | 8 | 0.001 |

**SI Table 6 Linear regression of mean absolute cellular elemental content with mean division rates at 27.4°C for all ten Symbiodiniaceae isolates.** *N*= 3 replicates per isolate.

| Elements | *r*.squared | adj.*r.*squared | sigma | statistic | *p.*value | df | logLik | AIC | BIC | deviance | df.residual |
| --- | --- | --- | --- | --- | --- | --- | --- | --- | --- | --- | --- |
| C | 0.298 | 0.211 | 0.013 | 3.401 | 0.102 | 1 | 30.544 | -55.087 | -54.179 | 0.001 | 8 |
| Ca | 0.125 | 0.015 | 0.014 | 1.138 | 0.317 | 1 | 29.438 | -52.875 | -51.967 | 0.002 | 8 |
| Cu | 0.010 | -0.114 | 0.015 | 0.078 | 0.787 | 1 | 28.821 | -51.642 | -50.734 | 0.002 | 8 |
| Fe | 0.061 | -0.056 | 0.015 | 0.522 | 0.491 | 1 | 29.088 | -52.176 | -51.269 | 0.002 | 8 |
| K | 0.098 | -0.015 | 0.014 | 0.870 | 0.378 | 1 | 29.289 | -52.577 | -51.670 | 0.002 | 8 |
| Mn | 0.270 | 0.179 | 0.013 | 2.961 | 0.124 | 1 | 30.347 | -54.694 | -53.786 | 0.001 | 8 |
| Mo | 0.709 | 0.673 | 0.008 | 19.505 | 0.002* | 1 | 34.947 | -63.894 | -62.986 | 0.001 | 8 |
| N | 0.419 | 0.347 | 0.012 | 5.779 | 0.043* | 1 | 31.491 | -56.982 | -56.074 | 0.001 | 8 |
| Ni | 0.140 | 0.032 | 0.014 | 1.300 | 0.287 | 1 | 29.525 | -53.051 | -52.143 | 0.002 | 8 |
| P | 0.208 | 0.109 | 0.014 | 2.105 | 0.185 | 1 | 29.940 | -53.881 | -52.973 | 0.001 | 8 |
| S | 0.088 | -0.027 | 0.015 | 0.767 | 0.407 | 1 | 29.230 | -52.461 | -51.553 | 0.002 | 8 |
| Se | 0.034 | -0.087 | 0.015 | 0.282 | 0.610 | 1 | 28.945 | -51.891 | -50.983 | 0.002 | 8 |
| Sr | 0.125 | 0.016 | 0.014 | 1.147 | 0.315 | 1 | 29.443 | -52.885 | -51.977 | 0.002 | 8 |
| V | 0.276 | 0.185 | 0.013 | 3.046 | 0.119 | 1 | 30.386 | -54.771 | -53.863 | 0.001 | 8 |
| Zn | 0.043 | -0.077 | 0.015 | 0.358 | 0.566 | 1 | 28.992 | -51.983 | -51.075 | 0.002 | 8 |

**SI Table 7** **Differences in element normalised to phosphorous (E:P) for the two putative 1-C to 1-Q functional Symbiodiniaceae clusters (see clustering in Fig. 2).** Differences between groups was assessed by Analysis of Variance (ANOVA) or Kruskal-Wallis test depending on whether parametric test assumptions were fulfilled. Levene’s test was applied to assess for equal variance, and Shapiro-Wilk for normality combined with manual inspections of QQ-plots of the model residuals. DFn denotes degrees of freedom, and * *p* ≤ 0.05.

| C (ANOVA) | | | |  | |  | |  |
| --- | --- | --- | --- | --- | --- | --- | --- | --- |
|  | | DFn | | *F* | | *p* | | *p* <.05 |
| Group | | 1 | | 0.061 | | 0.806 | |  |
| N (ANOVA) | | | |  | |  | |  |
|  | | DFn | | *F* | | *p* | | *p* <.05 |
| Group | | 1 | | 0.009 | | 0.927 | |  |
| S (ANOVA) | | | |  | |  | |  |
|  | | DFn | | *F* | | *p* | | *p* <.05 |
| Group | | 1 | | 59.44 | | 2.13E-08 | | * |
| K (ANOVA) | | | |  | |  | |  |
|  | | DFn | | *F* | | *p* | | *p* <.05 |
| Group | | 1 | | 0.057 | | 0.814 | |  |
| Ca (Kruskal-Wallis) | | | |  | |  | |  |
|  | | DFn | | *F* | | *p* | | *p* <.05 |
| Group | | 1 | | 6.308 | | 0.0181 | | * |
| V (ANOVA) | | | |  | |  | |  |
|  | | DFn | | *F* | | *p* | | *p* <.05 |
| Group | | 1 | | 1.532 | | 0.226 | |  |
|  |  | |  | |  | |  |  |
| Mn (ANOVA) | | DFn | | F | | *F* | | *p* |
|  | | 1 | | 1.399 | | 0.247 | |  |
| Fe (ANOVA) | | | |  | |  | |  |
|  | | DFn | | *F* | | *p* | | *p* <.05 |
| Group | | 1 | | 2.305 | | 0.14 | |  |
| Ni (ANOVA) | | | |  | |  | |  |
|  | | DFn | | *F* | | *p* | | *p* <.05 |
| Group | | 1 | | 6.977 | | 0.0134 | | * |
| Cu (ANOVA) | | | |  | |  | |  |
|  | | DFn | | *F* | | *p* | | *p* <.05 |
| Group | | 1 | | 3.268 | | 0.0814 | |  |
| Zn (ANOVA) | | | |  | |  | |  |
|  | | DFn | | *F* | | *p* | | *p* <.05 |
| Group | | 1 | | 1.284 | | 0.267 | |  |
| Se (ANOVA) | | | |  | |  | |  |
|  | | DFn | | *F* | | *p* | | *p* <.05 |
| Group | | 1 | | 5.423 | | 0.0273 | | * |
| Sr (Kruskal-Wallis) | | | |  | |  | |  |
|  | | DFn | | *F* | | *p* | | *p* <.05 |
| Group | | 1 | | 10.081 | | 0.001 | | * |
| Mo (ANOVA) | | | |  | |  | |  |
|  | | DFn | | *F* | | *p* | | *p* <.05 |
| Group | | 1 | | 0.262 | | 0.613 | |  |

**SI Table 8 Comparison of PSII maximum photochemical efficiency (*F_v_/F_m_*) for Symbiodiniaceae isolates SCF082, CCMP3420 and CCMP2464 at 27.4ºC and at 30.7ºC**. Differences were assessed by Kruskal-Wallis test with post hoc Dunn. df is degrees of freedom. Ns *p* > 0.05, * *p* ≤ 0.05 ** *p* ≤ 0.01 *** *p* ≤ 0.001.

| Isolate ID | n | statistic | df | *p* |
| --- | --- | --- | --- | --- |
| SCF082 | 6 | 3.857143 | 1 | 0.0495 |
| CCMP3420 | 6 | 3.857143 | 1 | 0.0495 |
| CCMP2464 | 6 | 2.333333 | 1 | 0.127 |

**SI Table 9 Comparison of cell volume and division rates of Symbiodiniaceae isolates SCF082, CCMP3420 and CCMP2464 at 27.4ºC and at 30.7ºC**. Differences were assessed by Kruskal-Wallis test with post hoc Dunn. df is degrees of freedom. Ns *p* > 0.05, * *p* ≤ 0.05 ** *p* ≤ 0.01 *** *p* ≤ 0.001.

| Cell volume | | | |  | |  |
| --- | --- | --- | --- | --- | --- | --- |
| Isolate ID | **n** | **statistic** | **df** | | ***p*** | |
| SCF082 | 6 | 0.428571 | 1 | | 0.513 | |
| CCMP3420 | 6 | 2.333333 | 1 | | 0.127 | |
| CCMP2464 | 6 | 0.047619 | 1 | | 0.827 | |
| Division rate | |  |  | |  | |
| Isolate ID | **n** | **statistic** | **df** | | ***p*** | |
| CCMP3420 | 6 | 0.428571 | 1 | | 0.513 | |
| CCMP2464 | 6 | 3.857143 | 1 | | 0.0495* | |
| SCF082 | 6 | 3.857143 | 1 | | 0.0495* | |

**SI Table 10 Assessment of differences in E:P ratios for the three Symbiodiniaceae isolates (SCF082, CCMP3420 and CCMP2464) at 27.4ºC and at 30.7ºC.** Differences were assessed via Analysis of Variance (ANOVA) with post hoc Tukey test. Levene’s test was applied to assess for equal variance, and Shapiro-Wilk for normality combined with manual inspections of QQ-plots of the model residuals. DFn is the degrees of freedom numerator, Dfd is the degrees of freedom denominator and ges is the generalised eta squared that is a useful metric for evaluating the size of an effect (>.26 considered large; Bakeman, 2005). Ns *p* > 0.05, * *p* ≤ 0.05 ** *p* ≤ 0.01 *** *p* ≤ 0.001.

A) ANOVA

| Elements | Effect | DFn | DFd | *F* | *p* | *p*<.05 | ges |
| --- | --- | --- | --- | --- | --- | --- | --- |
| C | Isolate_ID | 2 | 12 | 0.836 | 0.457 |  | 0.122 |
| C | Temperature | 1 | 12 | 3.408 | 0.09 |  | 0.221 |
| C | Isolate_ID:Temperature | 2 | 12 | 0.164 | 0.85 |  | 0.027 |
| Ca | Isolate_ID | 2 | 12 | 38.006 | 6.42E-06 | * | 0.864 |
| Ca | Temperature | 1 | 12 | 1.831 | 0.201 |  | 0.132 |
| Ca | Isolate_ID:Temperature | 2 | 12 | 0.3 | 0.746 |  | 0.048 |
| Cu | Isolate_ID | 2 | 12 | 6.089 | 0.015 | * | 0.504 |
| Cu | Temperature | 1 | 12 | 1.068 | 0.322 |  | 0.082 |
| Cu | Isolate_ID:Temperature | 2 | 12 | 0.041 | 0.96 |  | 0.007 |
| Fe | Isolate_ID | 2 | 12 | 0.273 | 0.765 |  | 0.044 |
| Fe | Temperature | 1 | 12 | 3.774 | 0.076 |  | 0.239 |
| Fe | Isolate_ID:Temperature | 2 | 12 | 0.24 | 0.79 |  | 0.038 |
| K | Isolate_ID | 2 | 12 | 2.198 | 0.154 |  | 0.268 |
| K | Temperature | 1 | 12 | 16.614 | 0.002 | * | 0.581 |
| K | Isolate_ID:Temperature | 2 | 12 | 2.108 | 0.164 |  | 0.26 |
| Mn | Isolate_ID | 2 | 12 | 0.078 | 0.926 |  | 0.013 |
| Mn | Temperature | 1 | 12 | 1.871 | 0.196 |  | 0.135 |
| Mn | Isolate_ID:Temperature | 2 | 12 | 2.52 | 0.122 |  | 0.296 |
| Mo | Isolate_ID | 2 | 12 | 12.211 | 0.001 | * | 0.671 |
| Mo | Temperature | 1 | 12 | 1.997 | 0.183 |  | 0.143 |
| Mo | Isolate_ID:Temperature | 2 | 12 | 2.03 | 0.174 |  | 0.253 |
| N | Isolate_ID | 2 | 12 | 1.744 | 0.216 |  | 0.225 |
| N | Temperature | 1 | 12 | 3.139 | 0.102 |  | 0.207 |
| N | Isolate_ID:Temperature | 2 | 12 | 0.693 | 0.519 |  | 0.104 |
| Ni | Isolate_ID | 2 | 12 | 0.018 | 0.982 |  | 0.003 |
| Ni | Temperature | 1 | 12 | 0.33 | 0.576 |  | 0.027 |
| Ni | Isolate_ID:Temperature | 2 | 12 | 1.741 | 0.217 |  | 0.225 |
| S | Isolate_ID | 2 | 12 | 39.282 | 5.41E-06 | * | 0.867 |
| S | Temperature | 1 | 12 | 79.37 | 1.23E-06 | * | 0.869 |
| S | Isolate_ID:Temperature | 2 | 12 | 10.426 | 0.002 | * | 0.635 |
| Se | Isolate_ID | 2 | 12 | 0.751 | 0.493 |  | 0.111 |
| Se | Temperature | 1 | 12 | 0.027 | 0.872 |  | 0.002 |
| Se | Isolate_ID:Temperature | 2 | 12 | 1.134 | 0.354 |  | 0.159 |
| Sr | Isolate_ID | 2 | 12 | 33.241 | 1.28E-05 | * | 0.847 |
| Sr | Temperature | 1 | 12 | 3.3 | 0.094 |  | 0.216 |
| Sr | Isolate_ID:Temperature | 2 | 12 | 0.249 | 0.783 |  | 0.04 |
| V | Isolate_ID | 2 | 12 | 5.029 | 0.026 | * | 0.456 |
| V | Temperature | 1 | 12 | 0.557 | 0.47 |  | 0.044 |
| V | Isolate_ID:Temperature | 2 | 12 | 4.479 | 0.035 | * | 0.427 |
| Zn | Isolate_ID | 2 | 12 | 1.919 | 0.189 |  | 0.242 |
| Zn | Temperature | 1 | 12 | 0.039 | 0.848 |  | 0.003 |
| Zn | Isolate_ID:Temperature | 2 | 12 | 1.264 | 0.318 |  | 0.174 |

1. Tukey post hoc test

| Elements | Term | group1 | group2 | *p*.adj |
| --- | --- | --- | --- | --- |
| C | Isolate_ID | CCMP3420 | CCMP2464 | 0.543 |
| C | Isolate_ID | CCMP3420 | SCF082 | 0.997 |
| C | Isolate_ID | CCMP2464 | SCF082 | 0.5 |
| C | Temperature | 27 | 31 | 0.0897 |
| C | Isolate_ID:Temperature | CCMP3420:27 | CCMP2464:27 | 0.971 |
| C | Isolate_ID:Temperature | CCMP3420:27 | SCF082:27 | 1 |
| C | Isolate_ID:Temperature | CCMP3420:27 | CCMP3420:31 | 0.953 |
| C | Isolate_ID:Temperature | CCMP3420:27 | CCMP2464:31 | 1 |
| C | Isolate_ID:Temperature | CCMP3420:27 | SCF082:31 | 0.809 |
| C | Isolate_ID:Temperature | CCMP2464:27 | SCF082:27 | 0.997 |
| C | Isolate_ID:Temperature | CCMP2464:27 | CCMP3420:31 | 0.616 |
| C | Isolate_ID:Temperature | CCMP2464:27 | CCMP2464:31 | 0.958 |
| C | Isolate_ID:Temperature | CCMP2464:27 | SCF082:31 | 0.399 |
| C | Isolate_ID:Temperature | SCF082:27 | CCMP3420:31 | 0.857 |
| C | Isolate_ID:Temperature | SCF082:27 | CCMP2464:31 | 0.999 |
| C | Isolate_ID:Temperature | SCF082:27 | SCF082:31 | 0.652 |
| C | Isolate_ID:Temperature | CCMP3420:31 | CCMP2464:31 | 0.966 |
| C | Isolate_ID:Temperature | CCMP3420:31 | SCF082:31 | 0.998 |
| C | Isolate_ID:Temperature | CCMP2464:31 | SCF082:31 | 0.841 |
| Ca | Isolate_ID | CCMP3420 | CCMP2464 | 0.00346 |
| Ca | Isolate_ID | CCMP3420 | SCF082 | 0.00178 |
| Ca | Isolate_ID | CCMP2464 | SCF082 | 4.31E-06 |
| Ca | Temperature | 27 | 31 | 0.201 |
| Ca | Isolate_ID:Temperature | CCMP3420:27 | CCMP2464:27 | 0.231 |
| Ca | Isolate_ID:Temperature | CCMP3420:27 | SCF082:27 | 0.0406 |
| Ca | Isolate_ID:Temperature | CCMP3420:27 | CCMP3420:31 | 1 |
| Ca | Isolate_ID:Temperature | CCMP3420:27 | CCMP2464:31 | 0.0266 |
| Ca | Isolate_ID:Temperature | CCMP3420:27 | SCF082:31 | 0.143 |
| Ca | Isolate_ID:Temperature | CCMP2464:27 | SCF082:27 | 0.000816 |
| Ca | Isolate_ID:Temperature | CCMP2464:27 | CCMP3420:31 | 0.322 |
| Ca | Isolate_ID:Temperature | CCMP2464:27 | CCMP2464:31 | 0.762 |
| Ca | Isolate_ID:Temperature | CCMP2464:27 | SCF082:31 | 0.00271 |
| Ca | Isolate_ID:Temperature | SCF082:27 | CCMP3420:31 | 0.0271 |
| Ca | Isolate_ID:Temperature | SCF082:27 | CCMP2464:31 | 0.000121 |
| Ca | Isolate_ID:Temperature | SCF082:27 | SCF082:31 | 0.968 |
| Ca | Isolate_ID:Temperature | CCMP3420:31 | CCMP2464:31 | 0.0399 |
| Ca | Isolate_ID:Temperature | CCMP3420:31 | SCF082:31 | 0.098 |
| Ca | Isolate_ID:Temperature | CCMP2464:31 | SCF082:31 | 0.000354 |
| Cu | Isolate_ID | CCMP3420 | CCMP2464 | 0.277 |
| Cu | Isolate_ID | CCMP3420 | SCF082 | 0.19 |
| Cu | Isolate_ID | CCMP2464 | SCF082 | 0.0116 |
| Cu | Temperature | 27 | 31 | 0.322 |
| Cu | Isolate_ID:Temperature | CCMP3420:27 | CCMP2464:27 | 0.903 |
| Cu | Isolate_ID:Temperature | CCMP3420:27 | SCF082:27 | 0.657 |
| Cu | Isolate_ID:Temperature | CCMP3420:27 | CCMP3420:31 | 0.999 |
| Cu | Isolate_ID:Temperature | CCMP3420:27 | CCMP2464:31 | 0.586 |
| Cu | Isolate_ID:Temperature | CCMP3420:27 | SCF082:31 | 0.972 |
| Cu | Isolate_ID:Temperature | CCMP2464:27 | SCF082:27 | 0.187 |
| Cu | Isolate_ID:Temperature | CCMP2464:27 | CCMP3420:31 | 0.985 |
| Cu | Isolate_ID:Temperature | CCMP2464:27 | CCMP2464:31 | 0.986 |
| Cu | Isolate_ID:Temperature | CCMP2464:27 | SCF082:31 | 0.522 |
| Cu | Isolate_ID:Temperature | SCF082:27 | CCMP3420:31 | 0.445 |
| Cu | Isolate_ID:Temperature | SCF082:27 | CCMP2464:31 | 0.0684 |
| Cu | Isolate_ID:Temperature | SCF082:27 | SCF082:31 | 0.966 |
| Cu | Isolate_ID:Temperature | CCMP3420:31 | CCMP2464:31 | 0.796 |
| Cu | Isolate_ID:Temperature | CCMP3420:31 | SCF082:31 | 0.864 |
| Cu | Isolate_ID:Temperature | CCMP2464:31 | SCF082:31 | 0.233 |
| Fe | Isolate_ID | CCMP3420 | CCMP2464 | 0.746 |
| Fe | Isolate_ID | CCMP3420 | SCF082 | 0.938 |
| Fe | Isolate_ID | CCMP2464 | SCF082 | 0.917 |
| Fe | Temperature | 27 | 31 | 0.0759 |
| Fe | Isolate_ID:Temperature | CCMP3420:27 | CCMP2464:27 | 0.905 |
| Fe | Isolate_ID:Temperature | CCMP3420:27 | SCF082:27 | 0.996 |
| Fe | Isolate_ID:Temperature | CCMP3420:27 | CCMP3420:31 | 0.613 |
| Fe | Isolate_ID:Temperature | CCMP3420:27 | CCMP2464:31 | 0.594 |
| Fe | Isolate_ID:Temperature | CCMP3420:27 | SCF082:31 | 0.606 |
| Fe | Isolate_ID:Temperature | CCMP2464:27 | SCF082:27 | 0.993 |
| Fe | Isolate_ID:Temperature | CCMP2464:27 | CCMP3420:31 | 0.99 |
| Fe | Isolate_ID:Temperature | CCMP2464:27 | CCMP2464:31 | 0.987 |
| Fe | Isolate_ID:Temperature | CCMP2464:27 | SCF082:31 | 0.989 |
| Fe | Isolate_ID:Temperature | SCF082:27 | CCMP3420:31 | 0.86 |
| Fe | Isolate_ID:Temperature | SCF082:27 | CCMP2464:31 | 0.846 |
| Fe | Isolate_ID:Temperature | SCF082:27 | SCF082:31 | 0.854 |
| Fe | Isolate_ID:Temperature | CCMP3420:31 | CCMP2464:31 | 1 |
| Fe | Isolate_ID:Temperature | CCMP3420:31 | SCF082:31 | 1 |
| Fe | Isolate_ID:Temperature | CCMP2464:31 | SCF082:31 | 1 |
| K | Isolate_ID | CCMP3420 | CCMP2464 | 0.133 |
| K | Isolate_ID | CCMP3420 | SCF082 | 0.612 |
| K | Isolate_ID | CCMP2464 | SCF082 | 0.515 |
| K | Temperature | 27 | 31 | 0.00154 |
| K | Isolate_ID:Temperature | CCMP3420:27 | CCMP2464:27 | 0.566 |
| K | Isolate_ID:Temperature | CCMP3420:27 | SCF082:27 | 0.997 |
| K | Isolate_ID:Temperature | CCMP3420:27 | CCMP3420:31 | 0.0934 |
| K | Isolate_ID:Temperature | CCMP3420:27 | CCMP2464:31 | 0.555 |
| K | Isolate_ID:Temperature | CCMP3420:27 | SCF082:31 | 0.849 |
| K | Isolate_ID:Temperature | CCMP2464:27 | SCF082:27 | 0.328 |
| K | Isolate_ID:Temperature | CCMP2464:27 | CCMP3420:31 | 0.00559 |
| K | Isolate_ID:Temperature | CCMP2464:27 | CCMP2464:31 | 0.0478 |
| K | Isolate_ID:Temperature | CCMP2464:27 | SCF082:31 | 0.118 |
| K | Isolate_ID:Temperature | SCF082:27 | CCMP3420:31 | 0.193 |
| K | Isolate_ID:Temperature | SCF082:27 | CCMP2464:31 | 0.81 |
| K | Isolate_ID:Temperature | SCF082:27 | SCF082:31 | 0.979 |
| K | Isolate_ID:Temperature | CCMP3420:31 | CCMP2464:31 | 0.791 |
| K | Isolate_ID:Temperature | CCMP3420:31 | SCF082:31 | 0.486 |
| K | Isolate_ID:Temperature | CCMP2464:31 | SCF082:31 | 0.993 |
| Mn | Isolate_ID | CCMP3420 | CCMP2464 | 0.993 |
| Mn | Isolate_ID | CCMP3420 | SCF082 | 0.922 |
| Mn | Isolate_ID | CCMP2464 | SCF082 | 0.961 |
| Mn | Temperature | 27 | 31 | 0.196 |
| Mn | Isolate_ID:Temperature | CCMP3420:27 | CCMP2464:27 | 0.635 |
| Mn | Isolate_ID:Temperature | CCMP3420:27 | SCF082:27 | 0.994 |
| Mn | Isolate_ID:Temperature | CCMP3420:27 | CCMP3420:31 | 0.999 |
| Mn | Isolate_ID:Temperature | CCMP3420:27 | CCMP2464:31 | 0.896 |
| Mn | Isolate_ID:Temperature | CCMP3420:27 | SCF082:31 | 0.999 |
| Mn | Isolate_ID:Temperature | CCMP2464:27 | SCF082:27 | 0.894 |
| Mn | Isolate_ID:Temperature | CCMP2464:27 | CCMP3420:31 | 0.829 |
| Mn | Isolate_ID:Temperature | CCMP2464:27 | CCMP2464:31 | 0.171 |
| Mn | Isolate_ID:Temperature | CCMP2464:27 | SCF082:31 | 0.84 |
| Mn | Isolate_ID:Temperature | SCF082:27 | CCMP3420:31 | 1 |
| Mn | Isolate_ID:Temperature | SCF082:27 | CCMP2464:31 | 0.638 |
| Mn | Isolate_ID:Temperature | SCF082:27 | SCF082:31 | 1 |
| Mn | Isolate_ID:Temperature | CCMP3420:31 | CCMP2464:31 | 0.726 |
| Mn | Isolate_ID:Temperature | CCMP3420:31 | SCF082:31 | 1 |
| Mn | Isolate_ID:Temperature | CCMP2464:31 | SCF082:31 | 0.713 |
| Mo | Isolate_ID | CCMP3420 | CCMP2464 | 0.0767 |
| Mo | Isolate_ID | CCMP3420 | SCF082 | 0.0647 |
| Mo | Isolate_ID | CCMP2464 | SCF082 | 0.000919 |
| Mo | Temperature | 27 | 31 | 0.183 |
| Mo | Isolate_ID:Temperature | CCMP3420:27 | CCMP2464:27 | 0.0742 |
| Mo | Isolate_ID:Temperature | CCMP3420:27 | SCF082:27 | 0.941 |
| Mo | Isolate_ID:Temperature | CCMP3420:27 | CCMP3420:31 | 0.248 |
| Mo | Isolate_ID:Temperature | CCMP3420:27 | CCMP2464:31 | 0.158 |
| Mo | Isolate_ID:Temperature | CCMP3420:27 | SCF082:31 | 0.999 |
| Mo | Isolate_ID:Temperature | CCMP2464:27 | SCF082:27 | 0.0165 |
| Mo | Isolate_ID:Temperature | CCMP2464:27 | CCMP3420:31 | 0.967 |
| Mo | Isolate_ID:Temperature | CCMP2464:27 | CCMP2464:31 | 0.996 |
| Mo | Isolate_ID:Temperature | CCMP2464:27 | SCF082:31 | 0.0434 |
| Mo | Isolate_ID:Temperature | SCF082:27 | CCMP3420:31 | 0.0609 |
| Mo | Isolate_ID:Temperature | SCF082:27 | CCMP2464:31 | 0.0366 |
| Mo | Isolate_ID:Temperature | SCF082:27 | SCF082:31 | 0.991 |
| Mo | Isolate_ID:Temperature | CCMP3420:31 | CCMP2464:31 | 1 |
| Mo | Isolate_ID:Temperature | CCMP3420:31 | SCF082:31 | 0.153 |
| Mo | Isolate_ID:Temperature | CCMP2464:31 | SCF082:31 | 0.0946 |
| N | Isolate_ID | CCMP3420 | CCMP2464 | 0.22 |
| N | Isolate_ID | CCMP3420 | SCF082 | 0.926 |
| N | Isolate_ID | CCMP2464 | SCF082 | 0.373 |
| N | Temperature | 27 | 31 | 0.102 |
| N | Isolate_ID:Temperature | CCMP3420:27 | CCMP2464:27 | 0.44 |
| N | Isolate_ID:Temperature | CCMP3420:27 | SCF082:27 | 0.897 |
| N | Isolate_ID:Temperature | CCMP3420:27 | CCMP3420:31 | 1 |
| N | Isolate_ID:Temperature | CCMP3420:27 | CCMP2464:31 | 0.994 |
| N | Isolate_ID:Temperature | CCMP3420:27 | SCF082:31 | 0.991 |
| N | Isolate_ID:Temperature | CCMP2464:27 | SCF082:27 | 0.945 |
| N | Isolate_ID:Temperature | CCMP2464:27 | CCMP3420:31 | 0.404 |
| N | Isolate_ID:Temperature | CCMP2464:27 | CCMP2464:31 | 0.733 |
| N | Isolate_ID:Temperature | CCMP2464:27 | SCF082:31 | 0.202 |
| N | Isolate_ID:Temperature | SCF082:27 | CCMP3420:31 | 0.87 |
| N | Isolate_ID:Temperature | SCF082:27 | CCMP2464:31 | 0.995 |
| N | Isolate_ID:Temperature | SCF082:27 | SCF082:31 | 0.608 |
| N | Isolate_ID:Temperature | CCMP3420:31 | CCMP2464:31 | 0.99 |
| N | Isolate_ID:Temperature | CCMP3420:31 | SCF082:31 | 0.995 |
| N | Isolate_ID:Temperature | CCMP2464:31 | SCF082:31 | 0.873 |
| Ni | Isolate_ID | CCMP3420 | CCMP2464 | 0.991 |
| Ni | Isolate_ID | CCMP3420 | SCF082 | 0.981 |
| Ni | Isolate_ID | CCMP2464 | SCF082 | 0.998 |
| Ni | Temperature | 27 | 31 | 0.576 |
| Ni | Isolate_ID:Temperature | CCMP3420:27 | CCMP2464:27 | 0.816 |
| Ni | Isolate_ID:Temperature | CCMP3420:27 | SCF082:27 | 0.994 |
| Ni | Isolate_ID:Temperature | CCMP3420:27 | CCMP3420:31 | 0.583 |
| Ni | Isolate_ID:Temperature | CCMP3420:27 | CCMP2464:31 | 1 |
| Ni | Isolate_ID:Temperature | CCMP3420:27 | SCF082:31 | 0.95 |
| Ni | Isolate_ID:Temperature | CCMP2464:27 | SCF082:27 | 0.979 |
| Ni | Isolate_ID:Temperature | CCMP2464:27 | CCMP3420:31 | 0.998 |
| Ni | Isolate_ID:Temperature | CCMP2464:27 | CCMP2464:31 | 0.914 |
| Ni | Isolate_ID:Temperature | CCMP2464:27 | SCF082:31 | 0.999 |
| Ni | Isolate_ID:Temperature | SCF082:27 | CCMP3420:31 | 0.863 |
| Ni | Isolate_ID:Temperature | SCF082:27 | CCMP2464:31 | 1 |
| Ni | Isolate_ID:Temperature | SCF082:27 | SCF082:31 | 0.999 |
| Ni | Isolate_ID:Temperature | CCMP3420:31 | CCMP2464:31 | 0.72 |
| Ni | Isolate_ID:Temperature | CCMP3420:31 | SCF082:31 | 0.963 |
| Ni | Isolate_ID:Temperature | CCMP2464:31 | SCF082:31 | 0.988 |
| S | Isolate_ID | CCMP3420 | CCMP2464 | 0.39 |
| S | Isolate_ID | CCMP3420 | SCF082 | 4.53E-05 |
| S | Isolate_ID | CCMP2464 | SCF082 | 7.46E-06 |
| S | Temperature | 27 | 31 | 1.23E-06 |
| S | Isolate_ID:Temperature | CCMP3420:27 | CCMP2464:27 | 0.104 |
| S | Isolate_ID:Temperature | CCMP3420:27 | SCF082:27 | 0.000559 |
| S | Isolate_ID:Temperature | CCMP3420:27 | CCMP3420:31 | 0.00548 |
| S | Isolate_ID:Temperature | CCMP3420:27 | CCMP2464:31 | 0.00113 |
| S | Isolate_ID:Temperature | CCMP3420:27 | SCF082:31 | 2.95E-05 |
| S | Isolate_ID:Temperature | CCMP2464:27 | SCF082:27 | 1.22E-05 |
| S | Isolate_ID:Temperature | CCMP2464:27 | CCMP3420:31 | 0.000073 |
| S | Isolate_ID:Temperature | CCMP2464:27 | CCMP2464:31 | 2.11E-05 |
| S | Isolate_ID:Temperature | CCMP2464:27 | SCF082:31 | 1.21E-06 |
| S | Isolate_ID:Temperature | SCF082:27 | CCMP3420:31 | 0.695 |
| S | Isolate_ID:Temperature | SCF082:27 | CCMP2464:31 | 0.996 |
| S | Isolate_ID:Temperature | SCF082:27 | SCF082:31 | 0.316 |
| S | Isolate_ID:Temperature | CCMP3420:31 | CCMP2464:31 | 0.913 |
| S | Isolate_ID:Temperature | CCMP3420:31 | SCF082:31 | 0.0317 |
| S | Isolate_ID:Temperature | CCMP2464:31 | SCF082:31 | 0.16 |
| Se | Isolate_ID | CCMP3420 | CCMP2464 | 0.961 |
| Se | Isolate_ID | CCMP3420 | SCF082 | 0.649 |
| Se | Isolate_ID | CCMP2464 | SCF082 | 0.492 |
| Se | Temperature | 27 | 31 | 0.872 |
| Se | Isolate_ID:Temperature | CCMP3420:27 | CCMP2464:27 | 0.956 |
| Se | Isolate_ID:Temperature | CCMP3420:27 | SCF082:27 | 0.719 |
| Se | Isolate_ID:Temperature | CCMP3420:27 | CCMP3420:31 | 0.871 |
| Se | Isolate_ID:Temperature | CCMP3420:27 | CCMP2464:31 | 1 |
| Se | Isolate_ID:Temperature | CCMP3420:27 | SCF082:31 | 0.921 |
| Se | Isolate_ID:Temperature | CCMP2464:27 | SCF082:27 | 0.99 |
| Se | Isolate_ID:Temperature | CCMP2464:27 | CCMP3420:31 | 1 |
| Se | Isolate_ID:Temperature | CCMP2464:27 | CCMP2464:31 | 0.929 |
| Se | Isolate_ID:Temperature | CCMP2464:27 | SCF082:31 | 1 |
| Se | Isolate_ID:Temperature | SCF082:27 | CCMP3420:31 | 0.999 |
| Se | Isolate_ID:Temperature | SCF082:27 | CCMP2464:31 | 0.659 |
| Se | Isolate_ID:Temperature | SCF082:27 | SCF082:31 | 0.997 |
| Se | Isolate_ID:Temperature | CCMP3420:31 | CCMP2464:31 | 0.825 |
| Se | Isolate_ID:Temperature | CCMP3420:31 | SCF082:31 | 1 |
| Se | Isolate_ID:Temperature | CCMP2464:31 | SCF082:31 | 0.884 |
| Sr | Isolate_ID | CCMP3420 | CCMP2464 | 0.0174 |
| Sr | Isolate_ID | CCMP3420 | SCF082 | 0.00108 |
| Sr | Isolate_ID | CCMP2464 | SCF082 | 9.16E-06 |
| Sr | Temperature | 27 | 31 | 0.0943 |
| Sr | Isolate_ID:Temperature | CCMP3420:27 | CCMP2464:27 | 0.492 |
| Sr | Isolate_ID:Temperature | CCMP3420:27 | SCF082:27 | 0.0256 |
| Sr | Isolate_ID:Temperature | CCMP3420:27 | CCMP3420:31 | 0.995 |
| Sr | Isolate_ID:Temperature | CCMP3420:27 | CCMP2464:31 | 0.0556 |
| Sr | Isolate_ID:Temperature | CCMP3420:27 | SCF082:31 | 0.173 |
| Sr | Isolate_ID:Temperature | CCMP2464:27 | SCF082:27 | 0.00131 |
| Sr | Isolate_ID:Temperature | CCMP2464:27 | CCMP3420:31 | 0.773 |
| Sr | Isolate_ID:Temperature | CCMP2464:27 | CCMP2464:31 | 0.682 |
| Sr | Isolate_ID:Temperature | CCMP2464:27 | SCF082:31 | 0.00856 |
| Sr | Isolate_ID:Temperature | SCF082:27 | CCMP3420:31 | 0.011 |
| Sr | Isolate_ID:Temperature | SCF082:27 | CCMP2464:31 | 0.000151 |
| Sr | Isolate_ID:Temperature | SCF082:27 | SCF082:31 | 0.845 |
| Sr | Isolate_ID:Temperature | CCMP3420:31 | CCMP2464:31 | 0.126 |
| Sr | Isolate_ID:Temperature | CCMP3420:31 | SCF082:31 | 0.0776 |
| Sr | Isolate_ID:Temperature | CCMP2464:31 | SCF082:31 | 0.000807 |
| V | Isolate_ID | CCMP3420 | CCMP2464 | 0.0319 |
| V | Isolate_ID | CCMP3420 | SCF082 | 0.919 |
| V | Isolate_ID | CCMP2464 | SCF082 | 0.0638 |
| V | Temperature | 27 | 31 | 0.47 |
| V | Isolate_ID:Temperature | CCMP3420:27 | CCMP2464:27 | 0.0125 |
| V | Isolate_ID:Temperature | CCMP3420:27 | SCF082:27 | 0.802 |
| V | Isolate_ID:Temperature | CCMP3420:27 | CCMP3420:31 | 0.201 |
| V | Isolate_ID:Temperature | CCMP3420:27 | CCMP2464:31 | 0.216 |
| V | Isolate_ID:Temperature | CCMP3420:27 | SCF082:31 | 0.504 |
| V | Isolate_ID:Temperature | CCMP2464:27 | SCF082:27 | 0.102 |
| V | Isolate_ID:Temperature | CCMP2464:27 | CCMP3420:31 | 0.563 |
| V | Isolate_ID:Temperature | CCMP2464:27 | CCMP2464:31 | 0.535 |
| V | Isolate_ID:Temperature | CCMP2464:27 | SCF082:31 | 0.234 |
| V | Isolate_ID:Temperature | SCF082:27 | CCMP3420:31 | 0.812 |
| V | Isolate_ID:Temperature | SCF082:27 | CCMP2464:31 | 0.834 |
| V | Isolate_ID:Temperature | SCF082:27 | SCF082:31 | 0.993 |
| V | Isolate_ID:Temperature | CCMP3420:31 | CCMP2464:31 | 1 |
| V | Isolate_ID:Temperature | CCMP3420:31 | SCF082:31 | 0.979 |
| V | Isolate_ID:Temperature | CCMP2464:31 | SCF082:31 | 0.984 |
| Zn | Isolate_ID | CCMP3420 | CCMP2464 | 0.569 |
| Zn | Isolate_ID | CCMP3420 | SCF082 | 0.638 |
| Zn | Isolate_ID | CCMP2464 | SCF082 | 0.165 |
| Zn | Temperature | 27 | 31 | 0.848 |
| Zn | Isolate_ID:Temperature | CCMP3420:27 | CCMP2464:27 | 0.47 |
| Zn | Isolate_ID:Temperature | CCMP3420:27 | SCF082:27 | 1 |
| Zn | Isolate_ID:Temperature | CCMP3420:27 | CCMP3420:31 | 0.795 |
| Zn | Isolate_ID:Temperature | CCMP3420:27 | CCMP2464:31 | 0.944 |
| Zn | Isolate_ID:Temperature | CCMP3420:27 | SCF082:31 | 1 |
| Zn | Isolate_ID:Temperature | CCMP2464:27 | SCF082:27 | 0.448 |
| Zn | Isolate_ID:Temperature | CCMP2464:27 | CCMP3420:31 | 0.99 |
| Zn | Isolate_ID:Temperature | CCMP2464:27 | CCMP2464:31 | 0.917 |
| Zn | Isolate_ID:Temperature | CCMP2464:27 | SCF082:31 | 0.473 |
| Zn | Isolate_ID:Temperature | SCF082:27 | CCMP3420:31 | 0.775 |
| Zn | Isolate_ID:Temperature | SCF082:27 | CCMP2464:31 | 0.934 |
| Zn | Isolate_ID:Temperature | SCF082:27 | SCF082:31 | 1 |
| Zn | Isolate_ID:Temperature | CCMP3420:31 | CCMP2464:31 | 0.999 |
| Zn | Isolate_ID:Temperature | CCMP3420:31 | SCF082:31 | 0.799 |
| Zn | Isolate_ID:Temperature | CCMP2464:31 | SCF082:31 | 0.946 |

**SI Table 11** **Assessment of differences in coordinates for Principal Component 1 (PC1) and 2 (PC2) for Symbiodiniaceae isolates SCF082, CCMP3420 and CCMP2464 at 27.4ºC and at 30.7ºC.** Differences were assessed via a two-way Analysis of Variance (ANOVA) with post hoc Tukey test. Levene’s test was applied to assess for equal variance, and Shapiro-Wilk for normality combined with manual inspections of QQ-plots of the model residuals. DFn is the degrees of freedom numerator, Dfd is the degrees of freedom denominator and ges is the generalised eta squared that is a useful metric for evaluating the size of an effect (>.26 considered large; Bakeman, 2005). Ns *p* > 0.05, * *p* ≤ 0.05 ** *p* ≤ 0.01 *** *p* ≤ 0.001.

1. ANOVA

| PC1 |  |  |  |  |  |  |
| --- | --- | --- | --- | --- | --- | --- |
| Effect | **DFn** | **DFd** | ***F*** | ***p*** | ***p* <.05** | **ges** |
| Isolate ID | 2 | 12 | 28.812 | 2.62E-05 | * | 0.828 |
| Temperature | 1 | 12 | 0.002 | 0.967 | ns | 1.47E-04 |
| Isolate ID:Temperature | 2 | 12 | 2.425 | 0.13 | ns | 0.288 |
| PC2 |  |  |  |  |  |  |
| Isolate ID | **DFn** | **DFd** | ***F*** | ***p*** | ***p* <.05** | **ges** |
| Temperature | 2 | 12 | 0.171 | 0.845 | ns | 0.028 |
| Isolate ID:Temperature | 1 | 12 | 5.854 | 0.032 | * | 0.328 |
| Isolate ID | 2 | 12 | 0.487 | 0.626 | ns | 0.075 |

1. Tukey post hoc test PC1

| Term | group1 | group2 | *p*.adj | *p*.adj.signif |
| --- | --- | --- | --- | --- |
| Isolate ID | CCMP3420 | CCMP2464 | 0.00346 | ** |
| Isolate ID | CCMP3420 | SCF082 | 0.0132 | * |
| Isolate ID | CCMP2464 | SCF082 | 1.80E-05 | **** |
| Temperature | 27 | 31 | 0.967 | ns |
| Isolate ID:Temperature | CCMP3420:27 | CCMP2464:27 | 0.0078 | ** |
| Isolate ID:Temperature | CCMP3420:27 | SCF082:27 | 0.416 | ns |
| Isolate ID:Temperature | CCMP3420:27 | CCMP3420:31 | 0.785 | ns |
| Isolate ID:Temperature | CCMP3420:27 | CCMP2464:31 | 0.142 | ns |
| Isolate ID:Temperature | CCMP3420:27 | SCF082:31 | 0.626 | ns |
| Isolate ID:Temperature | CCMP2464:27 | SCF082:27 | 3.66E-04 | *** |
| Isolate ID:Temperature | CCMP2464:27 | CCMP3420:31 | 0.0681 | ns |
| Isolate ID:Temperature | CCMP2464:27 | CCMP2464:31 | 0.531 | ns |
| Isolate ID:Temperature | CCMP2464:27 | SCF082:31 | 6.42E-04 | *** |
| Isolate ID:Temperature | SCF082:27 | CCMP3420:31 | 0.0601 | ns |
| Isolate ID:Temperature | SCF082:27 | CCMP2464:31 | 0.00547 | ** |
| Isolate ID:Temperature | SCF082:27 | SCF082:31 | 0.999 | ns |
| Isolate ID:Temperature | CCMP3420:31 | CCMP2464:31 | 0.711 | ns |
| Isolate ID:Temperature | CCMP3420:31 | SCF082:31 | 0.112 | ns |
| Isolate ID:Temperature | CCMP2464:31 | SCF082:31 | 0.0103 | * |

1. Tukey post hoc test PC2

| Term | group1 | group2 | *p*.adj | *p*.adj.signif |
| --- | --- | --- | --- | --- |
| Isolate ID | CCMP3420 | CCMP2464 | 0.918 | ns |
| Isolate ID | CCMP3420 | SCF082 | 0.983 | ns |
| Isolate ID | CCMP2464 | SCF082 | 0.837 | ns |
| Temperature | 27 | 31 | 0.0323 | * |
| Isolate ID:Temperature | CCMP3420:27 | CCMP2464:27 | 0.998 | ns |
| Isolate ID:Temperature | CCMP3420:27 | SCF082:27 | 0.991 | ns |
| Isolate ID:Temperature | CCMP3420:27 | CCMP3420:31 | 0.323 | ns |
| Isolate ID:Temperature | CCMP3420:27 | CCMP2464:31 | 0.832 | ns |
| Isolate ID:Temperature | CCMP3420:27 | SCF082:31 | 0.485 | ns |
| Isolate ID:Temperature | CCMP2464:27 | SCF082:27 | 1 | ns |
| Isolate ID:Temperature | CCMP2464:27 | CCMP3420:31 | 0.527 | ns |
| Isolate ID:Temperature | CCMP2464:27 | CCMP2464:31 | 0.965 | ns |
| Isolate ID:Temperature | CCMP2464:27 | SCF082:31 | 0.715 | ns |
| Isolate ID:Temperature | SCF082:27 | CCMP3420:31 | 0.627 | ns |
| Isolate ID:Temperature | SCF082:27 | CCMP2464:31 | 0.988 | ns |
| Isolate ID:Temperature | SCF082:27 | SCF082:31 | 0.807 | ns |
| Isolate ID:Temperature | CCMP3420:31 | CCMP2464:31 | 0.921 | ns |
| Isolate ID:Temperature | CCMP3420:31 | SCF082:31 | 0.999 | ns |
| Isolate ID:Temperature | CCMP2464:31 | SCF082:31 | 0.986 | ns |

**SI Table 12 Loadings of E:P in the first (PC1) and second (PC2) Principal Components of the Principle Component Analysis (PCA) for Symbiodiniaceae isolates SCF082, CCMP3420 and CCMP2464 at 27.4ºC and at 30.7ºC.**

| Elements | PC1 | PC2 |
| --- | --- | --- |
| S | 1.024039 | 0.254777 |
| Ni | 0.866939 | 0.575654 |
| Sr | 0.829235 | 0.544862 |
| Fe | 0.805062 | 0.601519 |
| Se | 0.794935 | 0.489301 |
| N | 0.720931 | 0.105985 |
| Cu | 0.690705 | 0.010227 |
| Ca | 0.484456 | 0.852685 |
| C | 0.470875 | 0.155156 |
| Mn | 0.190947 | 0.759222 |
| K | 0.18119 | 0.361512 |
| Zn | 0.12036 | 0.727056 |
| Mo | 0.094166 | 0.313109 |
| V | 0.087333 | 0.864925 |

**SI Table 13** **Elemental and vitamin content of the filtered artificial seawater enriched with Daigo’s IMK medium**. C and N were measured by dry combustion with a Trumac® CN analyser (Leco® Castle Hill, Australia). All other elements were measured by ICP-MS.

| Element/Vitamin | nM |
| --- | --- |
| C | 5609094.7 |
| N | 357583.1 |
| P | 73325.8 |
| S | 9823645.7 |
| Ca | 5893510.2 |
| V | 702.8 |
| Mn | 1627.3 |
| Fe | 29287.0 |
| Ni | 368.0 |
| Co | 196.8 |
| Cu | 807.3 |
| Zn | 856.5 |
| Se | 107.6 |
| Sr | 96066.0 |
| Mo | 209.5 |
| Cd | 39.1 |
| Sn | 8.5 |
| Thiamin | 753.7 |
| Biotin | 6.1 |
| B12 | 1.1 |

**SI Table 14** **Isotopes analysed via ICP-MS(/MS), the linearity of the calibration curve and the limits of detection (LOD).** The elements P and S were analysing using an oxygen-mass shift to bypass spectral interferences. All other elements were analysed on-mass.

| Element | R^2^ | LOD (ng g^-1^) |
| --- | --- | --- |
| ^31^P (analysed as ^31^P^16^O) | 0.9997 | 12.0 |
| ^32^S (analysed as ^32^S^16^O) | 0.9999 | 65.4 |
| ^39^K | 0.9991 | 43.1 |
| ^44^Ca | 0.9998 | 6.4 |
| ^55^Mn | 0.9991 | 9.9 |
| ^57^Fe | 0.9975 | 105.9 |
| ^63^Cu | 0.9978 | 6.9 |
| ^64^Zn | 0.9993 | 13.0 |
| ^78^Se | 0.9994 | 1.3 |
| ^86^Sr | 0.9999 | 4.3 |
| ^96^Mo | 0.9999 | 36.4 |
| ^51^V | 0.9996 | 1.56 |
| ^58^Ni | 0.9982 | 10.15 |

**SI Table 15** **Transformations of Symbiodiniaceae traits to homogenise variance based on the bestNormalise package in R (Peterson & Cavanaugh, 2019).** The package evaluates the normalisation efficacy of many candidate transformations, and selects the most effective using the Pearson P statistic.

| Trait | Transformation |
| --- | --- |
| Division rate | arcsinh_x |
| Cell volume | arcsinh_x |
| *F_v_/F_m_* | yeojohnson |
| σ | arcsinh_x |
| PQP_Size | sqrt_x |
| τ_1_ | arcsinh_x |
| τ_2_ | orderNorm |
| σLHCII | sqrt_x |
| τ_2_/PQ_OX_ | boxcox |
| 1-C | boxcox |
| 1-Q | orderNorm |

**References**

Bakeman, R. Recommended effect size statistics for repeated measures designs. Behavior research methods, 37(3), pp.379-384 (2005).

Kolber, Z. S., Prasil, O. & Falkowski, P. G. Measurements of variable chlorophyll fluorescence using fast repetition rate techniques: defining methodology and experimental protocols. Biochim. Biophys. Acta 1367, 88–106 (1998).

Osmond, B. et al. Relative functional and optical absorption cross-sections of PSII and other photosynthetic parameters monitored in situ, at a distance with a time resolution of a few seconds, using a prototype light induced fluorescence transient (LIFT) device. Funct. Plant Biol. 44, 985–1006 (2017).

Osmond, B., Chow, W. S., Pogson, B. J. & Robinson, S. A. Probing functional and optical cross-sections of PSII in leaves during state transitions using fast repetition rate light induced fluorescence transients. Funct. Plant Biol. 46, 567–583 (2019).

Suggett, D. J. et al. Functional diversity of photobiological traits within the genus Symbiodinium appears to be governed by the interaction of cell size with cladal designation. New Phytol. 208, 370–381 (2015).
